# Interventions in preconception and pregnant women at risk of gestational diabetes; a systematic review and meta-analysis of randomised controlled trials

**DOI:** 10.1186/s13098-023-01217-4

**Published:** 2024-01-04

**Authors:** Ola F. Quotah, Daria Andreeva, Katarzyna G. Nowak, Kathryn V. Dalrymple, Aljawharah Almubarak, Anjali Patel, Nirali Vyas, Gözde S. Cakir, Nicola Heslehurst, Zoe Bell, Lucilla Poston, Sara L. White, Angela C. Flynn

**Affiliations:** 1 Department of Women and Children’s Health, School of Life Course and Population Sciences, King’s College London, London, UK; 2 Department of Clinical Nutrition, Faculty of Applied Medical Science, King Abdulaziz University, Jeddah, Saudi Arabia; 3 Department of Nutrition and Dietetics, Cambridge University Hospitals NHS Foundation Trust, Cambridge, UK; 4 Department of Nutritional Sciences, School of Life Course Sciences and Population Sciences, King’s College London, London, UK; 5 Population Health Sciences Institute, Newcastle University, Newcastle Upon Tyne, UK; 6 School of Population Health, Royal College of Surgeons in Ireland, Dublin, Ireland

**Keywords:** Gestational diabetes, Intervention, Preconception, Pregnancy, Diet, Physical activity, Dietary supplement, Randomised controlled trials, Systematic review, Meta-analysis

## Abstract

**Background:**

Women at risk of gestational diabetes mellitus (GDM) need preventative interventions.

**Objective:**

To evaluate targeted interventions before and during pregnancy for women identified as being at risk of developing GDM.

**Methods:**

Systematic review and meta-analysis conducted following PRISMA guidelines. MEDLINE, EMBASE and the Cochrane Library in addition to reference and citation lists were searched to identify eligible randomised controlled trials (RCTs) utilising risk stratification during the preconception period or in the first/early second trimester. Screening and data extraction were carried out by the authors independently. Quality assessment was conducted based on the Cochrane risk-of-bias tool. Random effects meta-analysis and narrative synthesis were performed.

**Results:**

Eighty-four RCTs were included: two during preconception and 82 in pregnancy, with a pooled sample of 22,568 women. Interventions were behavioural (n = 54), dietary supplementation (n = 19) and pharmacological (n = 11). Predictive factors for risk assessment varied; only one study utilised a validated prediction model. Gestational diabetes was reduced in diet and physical activity interventions (risk difference − 0.03, 95% CI 0.06, − 0.01; I^2^ 58.69%), inositol (risk difference − 0.19, 95% CI 0.33, − 0.06; I^2^ 92.19%), and vitamin D supplements (risk difference − 0.16, 95% CI 0.25, − 0.06; I^2^ 32.27%). Subgroup analysis showed that diet and physical activity interventions were beneficial in women with ≥ 2 GDM risk factors (risk difference − 0.16, 95% CI 0.25, − 0.07; I^2^ 11.23%**)** while inositol supplementation was effective in women with overweight or obesity (risk difference − 0.17, 95% CI 0.22, − 0.11; I^2^ 0.01%). Effectiveness of all other interventions were not statistically significant.

**Conclusions:**

This review provides evidence that interventions targeted at women at risk of GDM may be an effective strategy for prevention. Further studies using validated prediction tools or multiple risk factors to target high-risk women for intervention before and during pregnancy are warranted.

**Supplementary Information:**

The online version contains supplementary material available at 10.1186/s13098-023-01217-4.

## Introduction

Gestational diabetes mellitus (GDM) is a common pregnancy related complication affecting ~ 14% of pregnancies worldwide, although prevalence varies by country, population and diagnostic criteria [1]. Women who develop GDM have a higher risk of gestational hypertension, pre-eclampsia, caesarean and preterm delivery than women who do not develop the condition [2–4]. Infants of mothers with GDM are at increased risk of stillbirth, macrosomia, and neonatal hypoglycaemia [3, 4]. In the longer term, GDM is associated with a greater risk of metabolic disease for both the mother and her offspring [5–7], highlighting the importance of early screening and prevention.

Although the aetiology of GDM is not completely understood, there are obstetric, socio-demographic, clinical and metabolic risk factors implicated [8, 9]. The oral glucose tolerance test (OGTT), usually carried out between 24 and 28 weeks of gestation, is used to detect GDM as part of routine antenatal care [10]. To date there is no consensus on the strategies to identify women at high-risk of developing GDM earlier in pregnancy.

Several antenatal trials have aimed to prevent GDM, suggesting that behavioural interventions (e.g. diet and physical activity (PA)), supplementation (e.g. myo-inositol and vitamin D), and pharmacological intervention using metformin have possible benefits in reducing risk in the general antenatal population [11–14]. Moreover, studies have not been able to establish an effect of diet or exercise alone, probiotics, and/or other vitamins and minerals on GDM risk [11, 15]. It is not yet clear whether targeting interventions to women with specific risk factors for GDM is an effective approach to GDM prevention.

Additionally, research on the effectiveness of preconception interventions in preventing GDM is lacking [11], and it is a priortiy area for intervention research [16]. One preconception nutritional intervention was not successful in reducing maternal glycaemia or GDM in a large multi-site trial; however, this study did not target higher risk women [17]. Hence, a more selective approach in women who are at risk and planning to conceive might be more effective.

The aim of this review was to evaluate the effect of interventions (behavioural, supplementation and pharmacological) during the preconception period and/or in pregnancy on reducing GDM in women identified at higher risk for developing the condition.

## Methods

This review was conducted in accordance with the relevant criteria of the PRISMA guidelines for reporting a systematic review and meta-analysis [18] and was registered in the PROSPERO database (CRD42020177976).

### Eligibility criteria

Inclusion and exclusion criteria were developed using the PICOS (population, intervention, comparison, outcomes and study design) framework, summarised in Table 1. For inclusion, studies had to meet the following criteria: (1) randomised controlled trials (RCTs) that evaluated interventions, in the preconception period and/or during pregnancy compared with no intervention, standard care or placebo; (2) women identified as higher risk of developing GDM using any risk stratification in the preconception period or in early pregnancy; (3) data reporting GDM as a primary or secondary pregnancy outcome. All diagnostic criteria for GDM were deemed acceptable. Studies meeting the following criteria were excluded: (1) non-randomised and observational studies; (2) abstracts, reviews, letters, commentaries and editorials; (3) women aged less than 18 years or older than 50 years; (4) studies designed to treat GDM; (5) interventions starting too late in pregnancy (> 28 weeks’ gestation); (6) studies not published in English.

**Table 1:** Summary of PICOS criteria for the inclusion of studies

Parameter	Description
Population	Preconception and/or pregnant women at higher risk of GDM, identified using risk factors including but not limited to overweight/obesity, raised lipids, elevated glucose concentration, insulin resistance, increased maternal age, high-risk ethnicity, previous macrosomic infant, previous GDM, PCOS, metabolic syndrome, hypertension, family history of GDM or diabetes, use of a risk tool
Intervention	Behavioural (diet/ PA/ diet and PA) and/or supplements and/or pharmacological intervention
Comparison	No intervention, standard care or placebo
Outcome	GDM as a primary or secondary outcome
Study design	Randomised controlled trials

*GDM* gestational diabetes mellitus; *PA* physical activity; *PCOS* polycystic ovary syndrome

### Database searches

Three electronic databases: MEDLINE, EMBASE and the Cochrane Central Register of Controlled Trials, were searched up to February 2023 with no date restriction. A comprehensive search strategy was developed (Additional file 1: Table A1, A2, A3) using search terms related to “pregnancy”, “adiposity” and “randomisation”. Reference lists from all included studies were examined for additional relevant articles to supplement the database searches as per PRISMA guideline recommendations [18]. Study authors were contacted when further information was required.

### Study selection

Records obtained from all databases were imported into the EndNote X9 reference management software to eliminate duplicate publications. Subsequently, studies were imported into the screening management software Rayyan [19] for screening. All title and abstracts were screened by OFQ and a second independent reviewer (either KGN, AA, GSC, AP or NV). Full-text screening was also carried out independently in duplicate, with disagreements discussed and resolved by consensus opinion among 4 reviewers.

### Data extraction and quality assessment

Data were extracted independently and in duplicate by two authors (OFQ, DA) using a standardised table created for this review. Data extraction included: title; authors; publication year; trial periods; study design; country; aim; sample size; population; inclusion/exclusion criteria; period of intervention (preconception and/or pregnancy); risk stratification; intervention characteristics and clinical outcomes. The Cochrane risk-of-bias tool for randomised trials (RoB 2) [20] was used to assess the validity and bias of each study included according to the Cochrane Handbook for Systematic Reviews of Interventions version 6.3 [21]. The domains used included randomisation bias (whether the allocation sequence was random and adequately concealed), deviations from the intended interventions (blinding of participants and trial personnel, adherence to intervention), bias due to missing outcome data (including biases introduced by procedures used to impute or otherwise account for missing outcome data), bias in measurements of the outcome (differential errors related to intervention assignment) and bias in selection of the reported results. The quality assessment was based on a series of signalling questions within each domain and was independently performed by two authors (OFQ, DA). Discrepancies were resolved by a third author (ACF). The overall risk was determined, and studies were classified as ‘low risk of bias’, ‘some concerns’ or ‘high risk of bias’.

### Analysis

The interventions and outcomes were evaluated to determine the appropriateness of data pooling in order to perform a meta-analysis. The analysis was built around different intervention types including: behavioural (diet only, PA only, combined diet and PA), dietary supplementation (inositols, vitamin D, fibre, probiotics), and pharmacological (metformin). Any intervention that could not contribute to the analysis or could not be pooled was excluded from the meta-analysis (e.g., studies that are not sufficiently homogeneous to be combined under the prespecified interventions, Table 1); and a narrative synthesis was performed to provide a brief summary of these studies and their findings [22]. Where appropriate, summaries of exposure effect for each intervention were provided using a risk difference, performed using Stata software, version 16.0 (StataCorp, College Station, TX, USA). A random-effects meta-analysis model was used to estimate the effects of each intervention on GDM and an I^2^ value greater than 50% was considered an indication of significant heterogeneity across studies [23]. Furthermore, separate analyses were performed limited to studies where increased body mass index (BMI ≥ 25 kg/m^2^ or ≥ 24 kg/m^2^ depending on classification used) was the only risk factor as criteria for intervention and studies that utilised more than one risk factor which may or may not have included BMI. Publication bias was investigated using Egger's test and funnel plots, if there were more than 10 RCTs per meta-analysis.

## Results

**Fig. 1:**
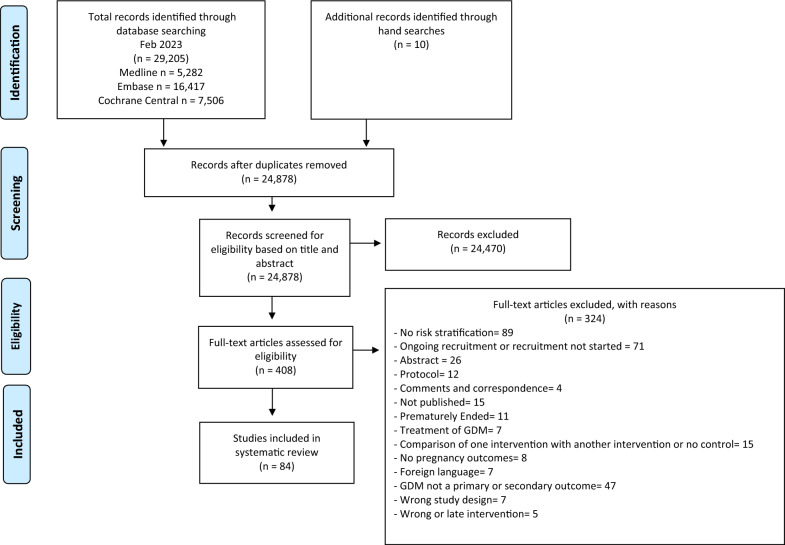
PRISMA flow diagram of screening, selection process and inclusion study

We identified 29,205 results through database searches and an additional 10 through citation searches and reference lists: 4,337 were removed as duplicates and of the remaining 24,878 articles, 24,470 were excluded during title and abstract screening. There were 408 full-texts screened against the eligibility criteria, and 84 met the inclusion criteria (Fig. 1). Major reasons for exclusion were: no risk stratification, ongoing RCTs and GDM not a primary/secondary outcome.

### Risk stratification for GDM

The included studies used different risk stratification strategies incorporating a variety of risk factors to identify women at high-risk of developing GDM. The number of variables ranged from one to sixteen (Additional file 1: Table A5). Fifty-four studies recruited women with increased BMI [24–77], with three of these using additional risk factors; polycystic ovary syndrome (PCOS) [41], previous history of GDM [41, 42, 56], family history of diabetes [41, 42], high-risk ethnicity [41] or history of unexplained intrauterine fetal death or macrosomic infant [41].

Two studies considered a family history of diabetes [78, 79], one utilised previous history of GDM [80] and one targeted women who had previously delivered an infant with macrosomia [81]. Three studies used elevated fasting blood glucose (FBG) [82–84] or/and haemoglobin A1c (HbA1c) [84]. Six studies used a history of PCOS [85–90] with Valdés et al. [90] additionally specifying pregestational insulin resistance (PIR) with at least one PIR clinical sign or the diagnosis of PCOS.

Fourteen studies required women to have at least one risk factor for the development of GDM including advanced maternal age [91–99], PCOS [93, 95, 98–100], BMI above a particular threshold (obesity/overweight) [91–97, 99–103], a family history of diabetes [91–96, 99–101, 104], signs of glucose intolerance [91, 92, 99], previous GDM [91–102, 104] or previous macrosomic infant [91–94, 96–101, 104]. Seven [94, 97–101, 103] of these specified other factors including high-risk ethnicity, chronic hypertension, twin pregnancies, abnormal lipid metabolism, glycosuria, previous pregnancy complications (e.g. gestational hypertension, pre-eclampsia, premature rupture of membranes, small for gestational age (SGA), large for gestational age (LGA), intrauterine growth restriction, low Apgar score, preterm deliveries, fetal anomaly, recurrent abortion, intrauterine fetal death and family history of either GDM or adverse obstetrical outcomes).

One study enrolled women with two or more GDM risk factors; including pre-pregnancy obesity, PCOS, high-risk ethnicity, previous GDM or macrosomic infant and family history of diabetes [105], while Mohsenzadeh-Ledari et al*.* [106] enrolled participants who had at least three components of the metabolic syndrome. One study used a validated prediction tool (simple scoring system) for identification of women at high-risk of developing GDM; this included history of GDM, maternal age, BMI, Asian descent, history of poor obstetric outcome and family history of diabetes [107].

### Characteristics of included studies

Characteristics of the studies are shown in Additional file 1: Table A4. The majority of the studies (n = 82) were RCTs targeting high-risk women in the antenatal period. Two studies in the preconception period were identified. Among the eighty-four studies included, fifteen were conducted in United States [29–31, 35, 42, 48, 49, 55, 56, 61, 62, 66, 71, 74, 84], eight in China [24, 25, 67, 73, 93, 95, 96, 99], eight in Italy [38, 39, 44, 59, 63, 78, 82, 83], eight in Australia [36, 47, 57, 58, 72, 75, 80, 107], seven in Iran [65, 68, 69, 88, 94, 98, 106], five in the United Kingdom [26, 40, 51, 100, 103], four in Finland [60, 91, 92, 102], four in the Republic of Ireland [27, 37, 79, 81], four in Denmark [33, 43, 46, 77], three in Norway [54, 85, 86], two in each of India [97, 101], Netherlands [41, 104], New Zealand [28, 64], Brazil [34, 50] and one in each of Canada [45], France [70], Chile [90], Belgium [53], Spain [32], Bangladesh [89] and the United Arab Emirates [105]. Three studies were multi-country; Norway, Sweden and Iceland [87]; United Kingdom, Republic of Ireland, Austria, Poland, Italy, Spain and Belgium [52, 76]; Netherlands, and Denmark [76].

The sample size of the studies ranged from 40 [86] to 2,122 [58] participants and the pooled sample size was 22568. Thirty studies included women of all BMI categories [78–107], while 54 included pregnant women living with overweight and/or obesity [24–77].

The intervention design varied between studies (Additional file 1: Table A5). The majority (n = 34) evaluated the effect of combined diet and PA [30–35, 37, 48, 51, 53, 55, 56, 58, 59, 61, 63, 65–67, 69–71, 74, 84, 91–93, 95, 96, 99, 102, 105–107]. Seven studies focused on modifying diet only [24, 29, 46, 49, 57, 81, 103], while ten aimed at modifying PA alone [25, 27, 45, 54, 62, 64, 72, 80, 97, 104]. A multidisciplinary approach (consisting of continuity of care, assessment of weight gain, a brief dietary intervention and psychological approach using solution-focused therapy) was used in one study [75]. Nine studies based their intervention on the supplement inositol (myo-inositol, d-chiro-inositol or combination of both) [38, 39, 44, 68, 78, 79, 82, 83, 100] while four used probiotic supplementation (Lactobacillus and Bifidobacterium species) [36, 60, 77, 98]. In three studies, the impact of vitamin D was assessed [52, 94, 101] and two evaluated the intake of soluble fibre [42, 73] of which one study additionally provided women with frozen blueberries [42]. Metformin was used as a pharmacological intervention in eleven RCTs [40, 41, 47, 50, 85–90, 108]. Three studies included more than two arms; Renault et al*.* [43] compared PA only and combined diet and PA with standard care, whilst Simmons et al*.* [76] compared the effect of diet alone, PA alone, combined diet and PA with standard care. Okesene-Gafa et al. [28] compared diet alone and probiotic to standard care and placebo arms.

### Risk of bias (quality) assessment

**Fig. 2:**
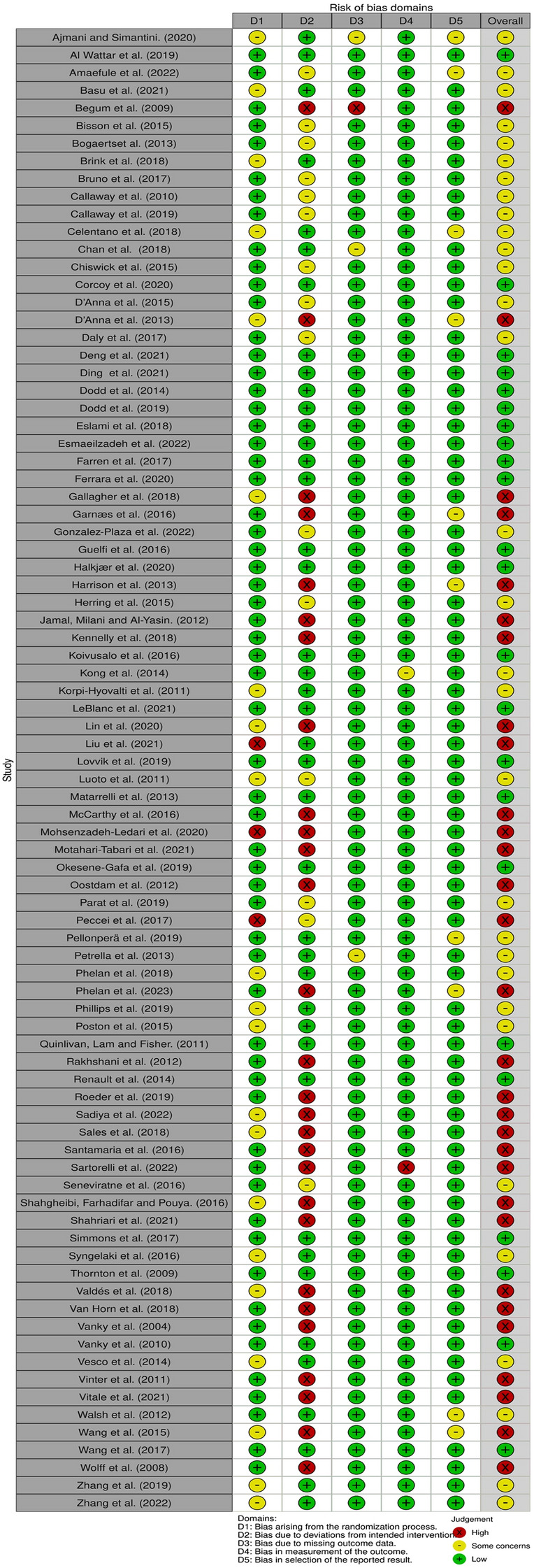
Quality assessment of intervention studies using Cochrane RoB 2- tool

The overall quality of the included studies varied and is summarised in Fig. 2. Twenty-three studies were assessed as ‘low risk of bias’ [25, 28, 31, 43, 47, 49, 52, 55, 58, 67–69, 75–77, 79, 80, 82, 85, 87, 99, 102, 103], 30 as ‘high risk of bias’ [30, 33–35, 37–39, 46, 50, 54, 56, 57, 61, 65, 66, 78, 84, 86, 88–90, 93–95, 97, 98, 104–107] and the remaining 31 studies as ‘some concerns’ [24, 26, 27, 29, 32, 36, 40–42, 44, 45, 48, 51, 53, 59, 60, 62–64, 70–74, 81, 83, 91, 92, 96, 100, 101]. The main source of bias across all studies was the non-adherence to the assigned intervention regimen.

### Behavioural intervention (diet only, PA only, combined diet and PA)

**Fig. 3:**
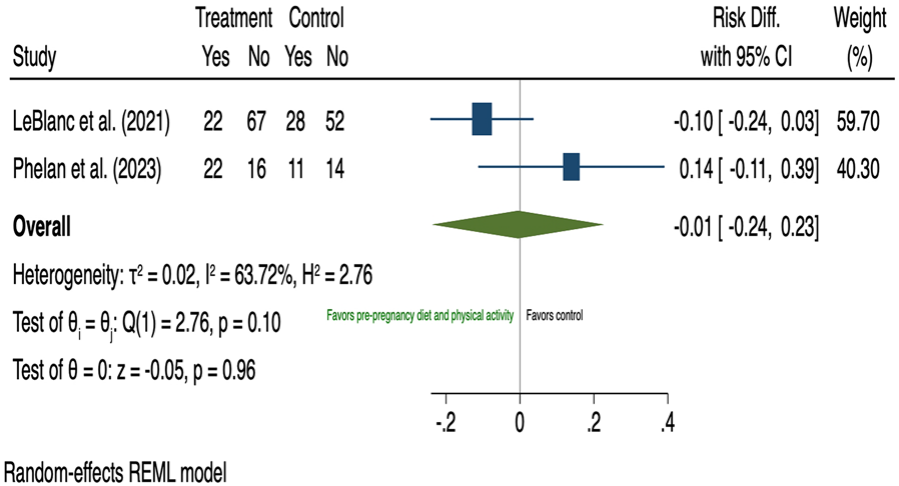
The effects of pre-pregnancy combined diet and physical activity intervention on GDM prevention

In preconception women, the two included studies [55, 56] assessed the impact of behavioural intervention (combined diet and PA) on GDM risk and were pooled in a meta-analysis (Additional file 1: Table A6). Diet and PA-based interventions prior to pregnancy did not reduce GDM development among those who were considered at higher risk prior to pregnancy (risk difference − 0.01, 95% CI 0.24 to 0.23; I^2^ 63.72%) (Fig. 3).

**Fig. 4:**
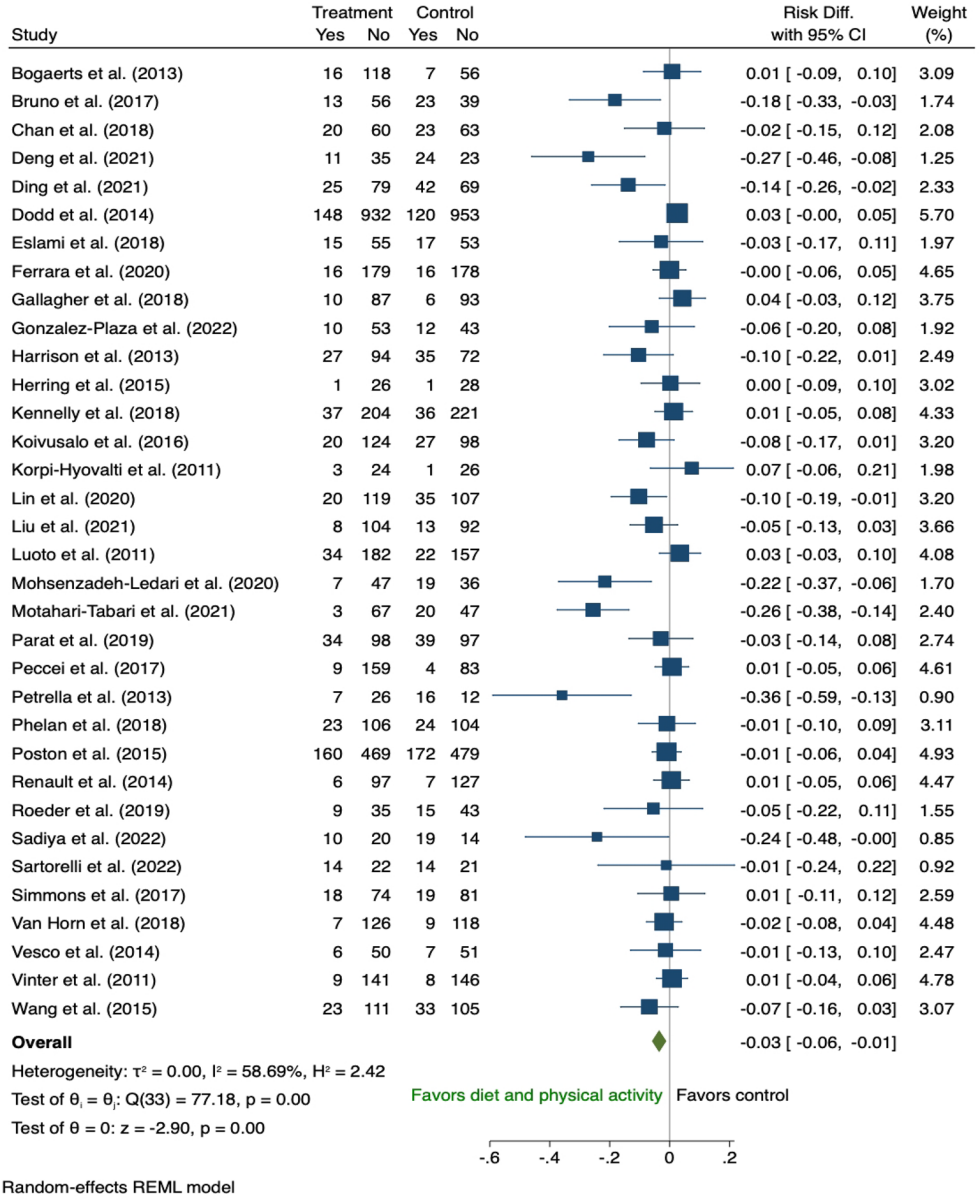
The effects of antenatal combined diet and physical activity intervention on GDM prevention

**Fig. 5:**
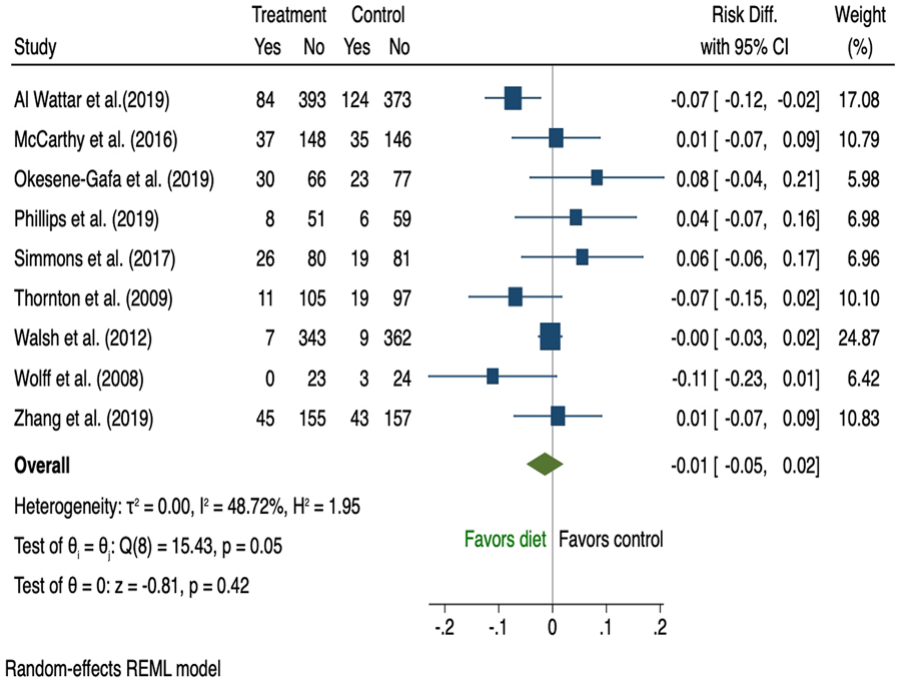
The effects of antenatal diet only intervention on GDM prevention

**Fig. 6:**
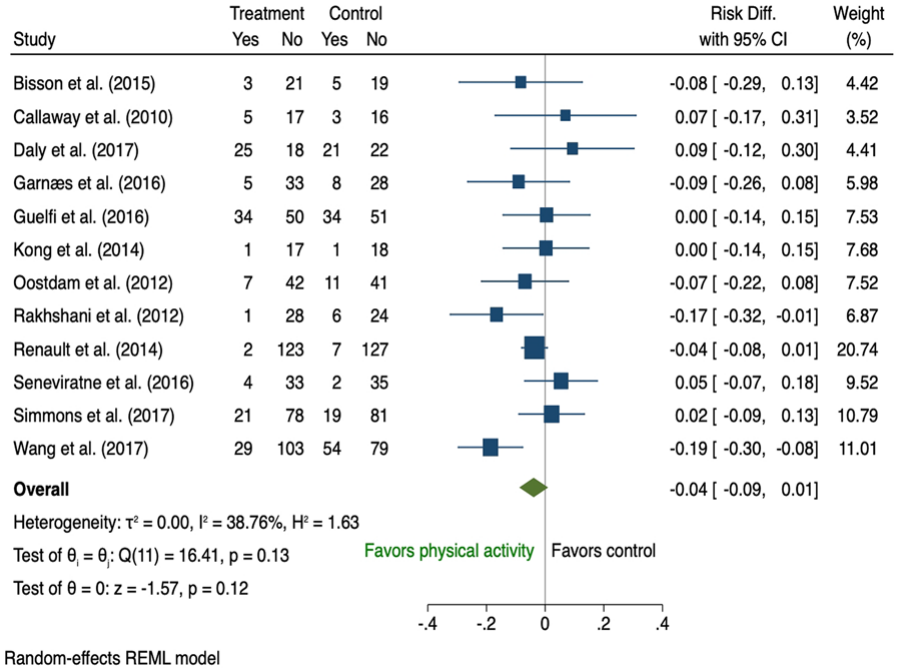
The effects of antenatal physical activity only intervention on GDM prevention

Fifty-three antenatal studies [24, 25, 27–35, 37, 43, 45, 46, 48, 49, 51, 53, 54, 57–59, 61–67, 69–72, 74–76, 80, 81, 84, 91–93, 95–97, 99, 102–107] reported the effect of a behavioural intervention during pregnancy on the development of GDM with 52 of those included in the meta-analysis (Additional file 1: Table A6). One study was considered insufficiently homogenous to be pooled for meta-analysis and was excluded from the analysis since only brief dietary intervention (5 min) was provided as part of the multidisciplinary approach [75]. In the 34 studies that examined the effect of combined diet and PA interventions on GDM risk [30–35, 37, 43, 48, 51, 53, 58, 59, 61, 63, 65–67, 69–71, 74, 76, 84, 91–93, 95, 96, 99, 102, 105–107], women who received the intervention were 3% less likely to develop GDM (risk difference − 0.03, 95% CI 0.06 to − 0.01; I^2^ 58.69%) with significant heterogeneity across studies (Fig. 4). Nine studies of diet only [24, 28, 29, 46, 49, 57, 76, 81, 103] (Fig. 5) and twelve of PA only [25, 27, 43, 45, 54, 62, 64, 72, 76, 80, 97, 104] interventions (Fig. 6), showed no significant difference in GDM risk (risk difference − 0.01, 95% CI 0.05 to 0.02; I^2^ 48.72 and − 0.04, 95% CI 0.09 to 0.01; I^2^ 38.76% respectively).

**Fig. 7:**
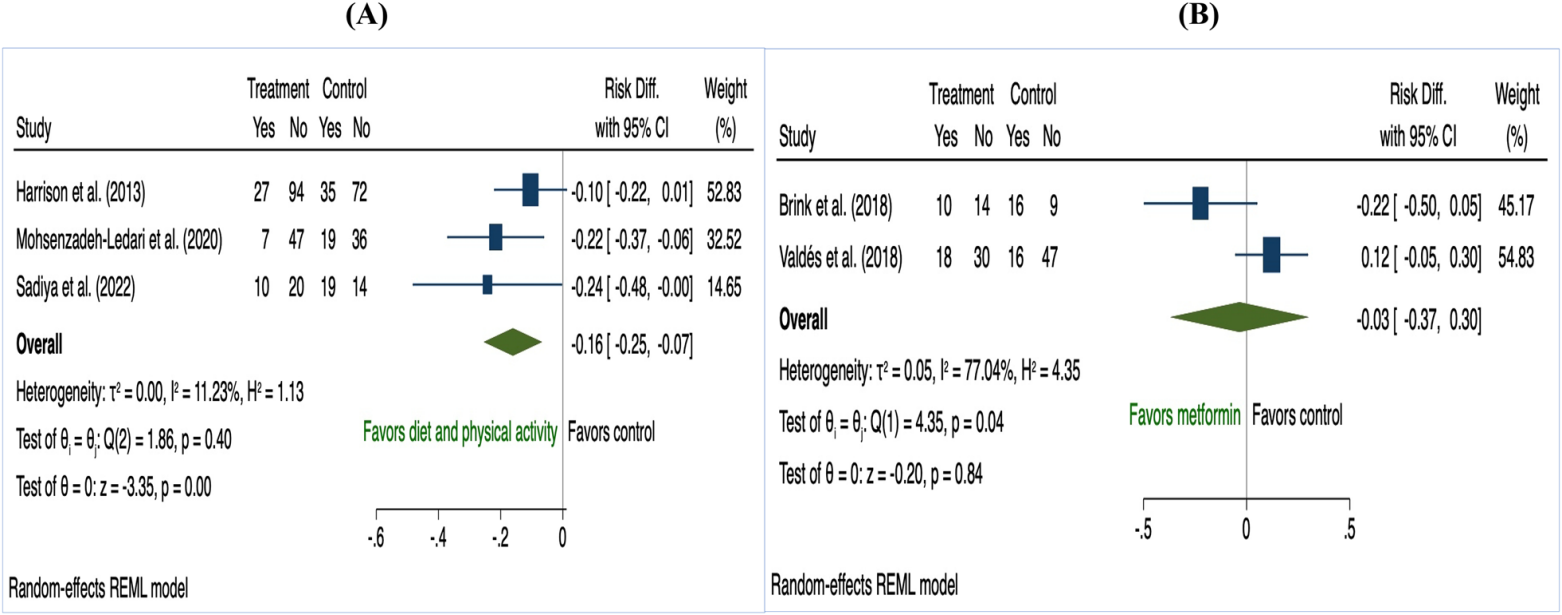
The effects of antenatal interventions **A** diet and physical activity **B** metformin on GDM prevention in women who had more than single risk factor

**Fig. 8:**
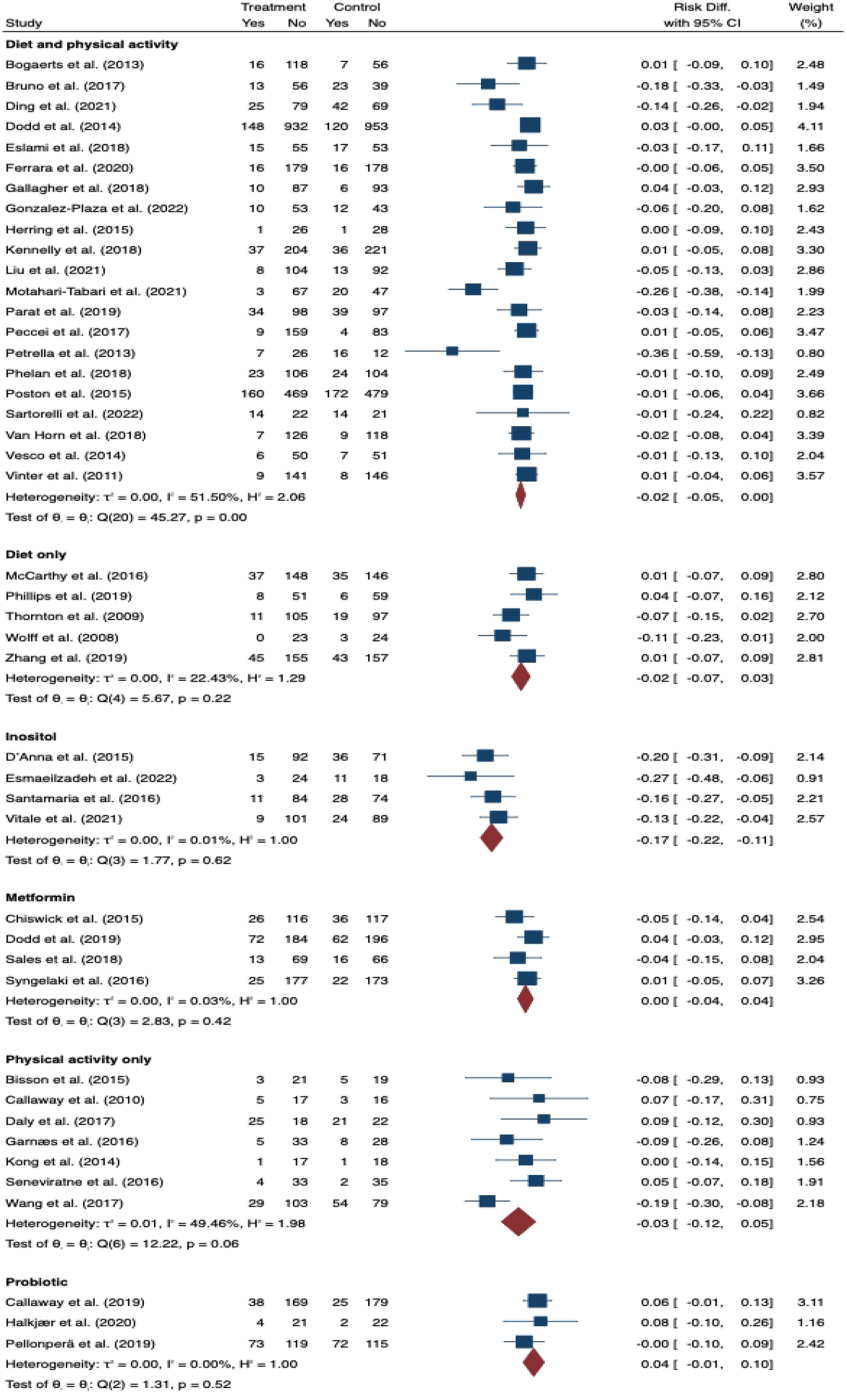
The effects of antenatal interventions including diet only, physical activity only, diet and physical activity, metformin, inositol and probiotic on GDM prevention when body mass index was considered as the only risk factor

In the subgroup analyses, three antenatal studies that used combined diet and PA interventions for women who had ≥ 2 risk factors reduced GDM risk (risk difference − 0.16, 95% CI 0.25 to − 0.07; I^2^ 11.23%; Fig. 7). There was no effect in sub-group analyses for BMI as the only risk factor (combined diet and PA: risk difference − 0.02, 95% CI − 0.05 to 0.00; I^2^ 51.50%; diet only: risk difference − 0.02, 95% CI − 0.07 to 0.03; I^2^ 22.43%; PA only: risk difference − 0.03, 95% CI − 0.12 to 0.05; I^2^ 49.46%; Fig. 8).

### Supplementation interventions (inositols, vitamin D, probiotics, fibre)

**Fig. 9:**
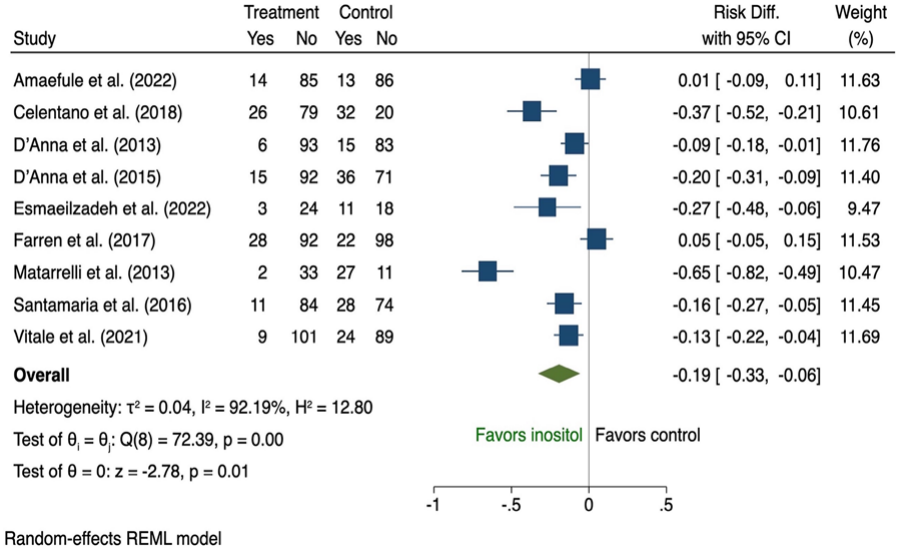
The effects of antenatal inositol supplementation on GDM prevention

**Fig. 10:**
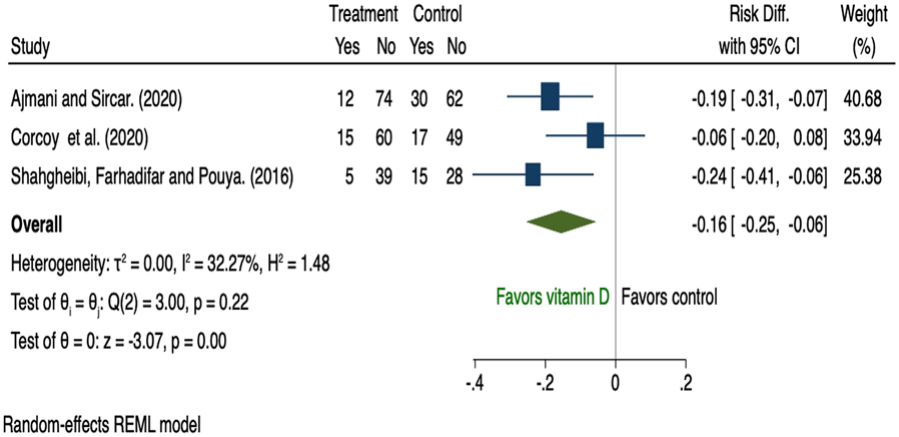
The effects of antenatal vitamin D supplementation on GDM prevention

**Fig. 11:**
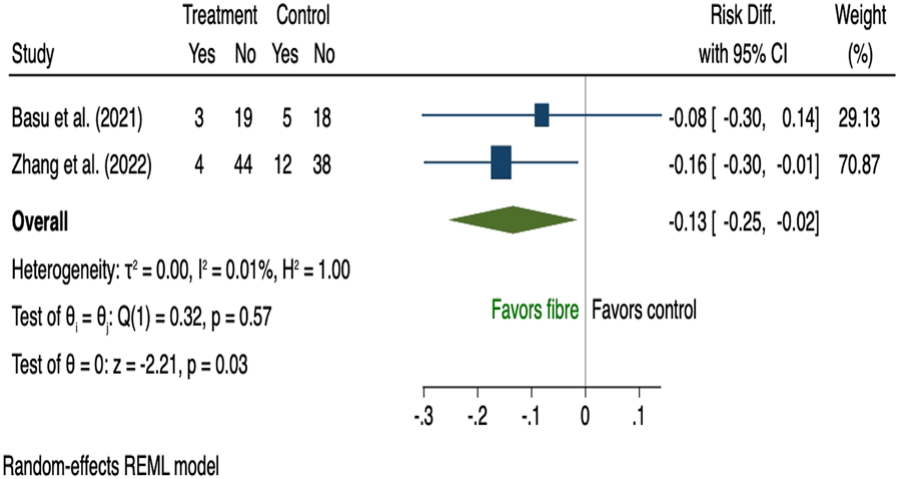
The effects of antenatal fibre supplementation on GDM prevention

**Fig. 12:**
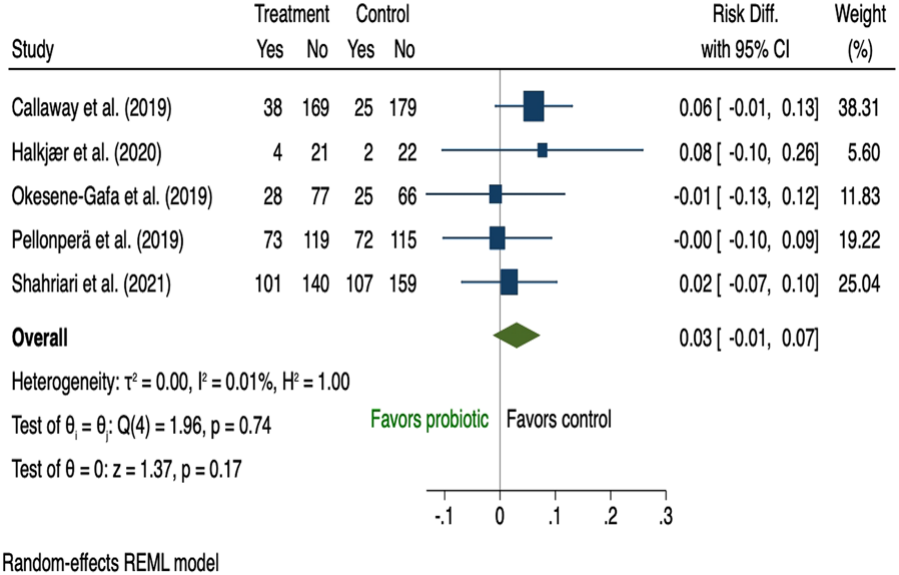
The effects of antenatal probiotics supplementation on GDM prevention

There were 19 studies [28, 36, 38, 39, 42, 44, 52, 60, 68, 73, 77–79, 82, 83, 94, 98, 100, 101] that assessed the effect of dietary supplementation on risk of GDM during pregnancy and all were pooled in the meta-analysis (Additional file 1: Table A6). Nine used inositol [38, 39, 44, 68, 78, 79, 82, 83, 100] and three vitamin D [52, 94, 101]; both of which reduced risk of GDM (risk difference − 0.19, 95% CI 0.33 to − 0.06; I^2^ 92.19 and − 0.16, 95% CI 0.25 to − 0.06; I^2^ 32.27% respectively); however, there was significant heterogeneity among the inositol interventions (Fig. 9, 10). Two studies that tested the use of fibre [42, 73] showed a reduction in GDM (risk difference − 0.13, 95% CI 0.25 to − 0.02; I^2^ 0.01%; Fig. 11). There was no evidence of an effect of probiotic use on the prevention of GDM (risk difference 0.03, 95% CI 0.01 to 0.07; I^2^ 0.01%; Fig. 12). In the subgroup analysis, four studies used inositol interventions where BMI was the only risk factor and demonstrated a reduction in GDM (risk difference − 0.17, 95% CI 0.22 to − 0.11; I^2^ 0.01%; Fig. 8). It was not possible to perform a sub-group analysis due to lack of supplementation studies among women with multiple risk factors.

### Pharmacological intervention

**Fig. 13:**
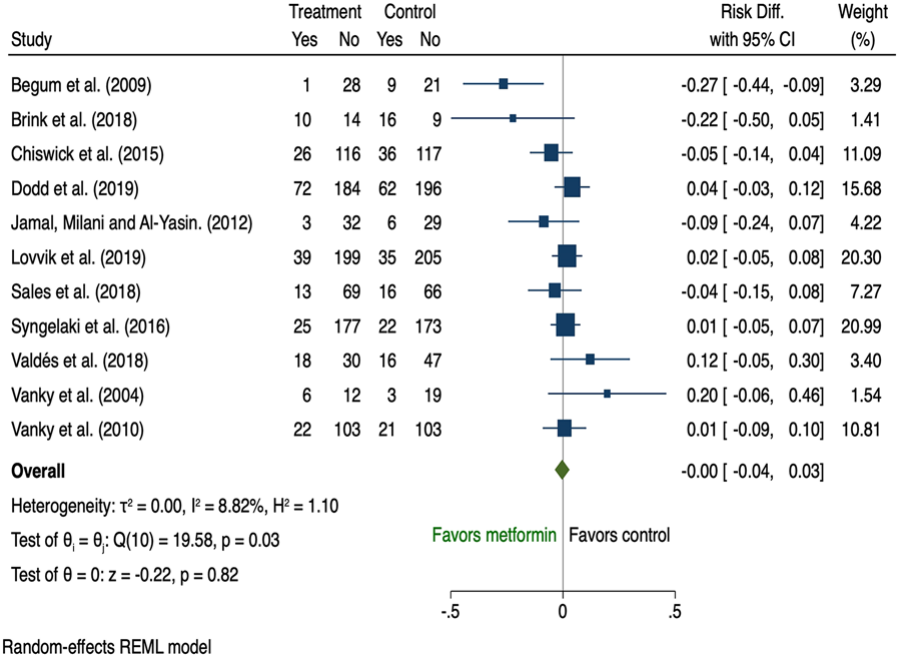
The effects of antenatal metformin intervention on GDM prevention

There were eleven studies [40, 41, 47, 50, 85–90, 108] that evaluated the effect of metformin on GDM and all were included in the meta-analysis (Additional file 1: Table A6). There was no significant effect of metformin in the prevention of GDM either overall (risk difference − 0.00, 95% CI − 0.04 to 0.03; I^2^ 8.82%; Fig. 13) or in the subgroup analyses of women with multiple risk factors (risk difference − 0.03, 95% CI − 0.37 to 0.30; I^2^ 77.04%; Fig. 7) or those with overweight and obesity (risk difference 0.00, 95% CI − 0.04 to 0.04; I^2^ 0.03%; Fig. 8).

### Narrative synthesis

It was not possible to conduct a meta-analysis for one study that used a multidisciplinary approach intervention. Therefore, data has been synthesised narratively. The study reported GDM as a primary outcome in pregnant women with increased BMI (> 25 kg/m^2^) and the intervention resulted in a significant reduction in the incidence of GDM (n = 4, 6% vs. n = 17, 29%, OR 0.17, 95% CI 0.03–0.95, *p* = 0.04) [75].

### Publication bias

There was no evidence of small study effects for any intervention except antenatal combined diet and PA interventions (*p* < 0.05), which may signal publication bias (Additional file 1: Figure A1, Table A7).

## Discussion

This review aimed to evaluate the effectiveness of preconception and pregnancy interventions in reducing GDM among women at increased risk. The findings from the meta-analysis showed that GDM was reduced using combined diet and PA, inositol and vitamin D supplementation in women identified in early pregnancy as higher risk. The effect was greatest with the dietary supplements. In a sub-group analysis, diet and PA interventions were most effective in women with multiple GDM risk factors while in pregnant women living with overweight and obesity, inositol was effective. The results were limited by high levels of heterogeneity between the included studies, while some studies were not sufficiently powered to detect a difference in GDM. Additionally, there was a lack of preconception studies.

Whilst this analysis showed that the use of antenatal combined diet and PA intervention modestly reduced the risk of GDM when higher risk women were recruited, this same approach did not appear effective when higher BMI was considered the sole risk factor. These findings are consistent with a meta-regression examining moderators of intervention effectiveness for preventing GDM; it showed that behavioural interventions in populations with higher risk of GDM demonstrated greater effectiveness, but also highlighted that BMI before or in early pregnancy was not related to the effect size of the intervention [109]. This suggests that BMI stratification alone is not an effective strategy for either risk prediction or response to intervention [109]. Conversely, the current review showed that using a targeted recruitment strategy that includes multiple risk factors for GDM helped maximise the effectiveness of a combined diet and PA intervention. Given the limited number of studies available (n = 3) and the high risk of bias associated with these studies, we still lack certainty and more research is needed to examine targted interventions particularly in women with multiple risk factors.

The current review found that antenatal interventions using inositol in higher-risk women, including those with increased BMI as a sole risk factor, were effective in reducing GDM. This correlates with findings of a recent meta-analysis of inositol interventions in ‌pregnant women living with overweight and obesity [110]. The insulin-mimetic effects of myo-inositol or its isomers are thought to be related to the production of inositol glycan secondary messengers, resulting in improved glycogen synthesis and glucose peripheral tissue uptake [111, 112]. Moreover, a deficit in intracellular d-chiro-inositol (DCI) has been observed in women with PCOS and overweight or obesity, resulting in impaired coupling between insulin action and the release of d-chiro-inositol-containing inositolphosphoglycan (DCI-IPG) that acts as an insulin mediator and sensitizer [113]. Although further studies of pregnant women with overweight and obesity are required to confirm inositol effectiveness, our analysis suggests that inositol supplementation may reduce the incidence of GDM among pregnant women at increased risk, regardless of the factors used in the risk assessment.

The present meta-analysis also suggests a preventative effect of antenatal vitamin D supplementation on GDM risk, and Cochrane reviews have also previously provided evidence for this possible benefit [11]. Several mechanistic pathways elucidating an influence of vitamin D on glucose homeostasis and GDM development have been described which impact upon metabolic markers including blood glucose concentrations, insulin resistance and inflammatory biomarkers [114–116]. Given that only three studies were identified in the current review that used vitamin D interventions, there is a need for further well-designed trials using larger cohorts among pregnant women identified as high-risk.

No effect of probiotics on the development of GDM in higher-risk women was found, consistent with the results of a Cochrane review [11]. However, in contrast, a recent meta-analysis (n = 10) showed a significant reduction of GDM with probiotic supplementation in the general antenatal population [117]. Furthermore, metformin was not found to be effective in preventing GDM in higher risk pregnant women. This is consistent with a previous meta-analysis in which the use of metformin started at conception or before 20 weeks of pregnancy did not reduce GDM when BMI was the only risk factor, or another selective risk assessment strategy such as PCOS and/or PIR was used [39]. A Cochrane systematic review of metformin use to prevent the development of type 2 diabetes mellitus (T2DM) (n = 20 RCTs) in individuals at increased risk supported efficacy in prevention (with or without behavioural interventions) when taken over a minimum of at least a 1-year [118]. This may imply that given the relatively short period of gestation, women at increased risk might benefit from metformin intervention if commenced during the preconception period. Moreover, our analysis highlights the limited evidence for metformin interventions (n = 2) in women with multiple risk factors, and the significant heterogeneity between them. Again, further research is needed as a potential effect of prolonged preconception metformin use in preventing GDM in such a population cannot be discounted.

Several different approaches for the identification of women at higher risk were utilised in the included studies for this review. Notably, only one study used a validated tool to identify risk while five studies intervened in women with ≥ 2 GDM risk factors. Our finding that interventions with women who had multiple risk factors reduced GDM prevalence supports the use of multiple risk factor clustering or validated tools to screen for risk of GDM. One such tool for identifying which women living with obesity are at higher risk of GDM development early in pregnancy has been developed [119] and is currently being validated. Better identification of risk amongst women with obesity, most of whom will not develop GDM, should facilitate interventions to be targeted at those most likely to benefit [119].

This review highlights the paucity of interventions targeting higher risk women in the preconception period, mirroring a lack of interventions globally in individuals preparing for pregnancy [120]. Moreover, we found no evidence of benefit of preconception behavioural interventions on the development of GDM in the two identified studies. The current interest in the importance of improving preconception health has stimulated recent attempts [121]. NiPPeR, a double-blind multicentre RCT in healthy women planning pregnancy, examined the effect of a nutritional formulation containing myo-inositol, probiotics, and multiple micronutrients taken preconception and throughout pregnancy, on gestational glycaemia and preterm delivery [17]. Whilst preterm delivery was reduced, there was no effect on the prevalence of GDM [17]. Additionally, NiPPeR did not demonstrate any benefit of the intervention in women with overweight or obesity or those with documented dysglycaemia; however, the trial was not powered to detect differences between subgroups [17]. Appropriately designed RCTs which encompass behavioural, supplementation and pharmacological interventions in high-risk women contemplating pregnancy are needed to evaluate the role of these interventions at the population level.

## Strengths and limitations

To date, this study represents the largest review on this subject, with the inclusion of recently published studies targeting higher risk antenatal populations. A robust comprehensive search strategy was utilised using well-defined eligibility criteria. The screening, risk of bias assessment and data extraction were performed independently in duplicate. However, meta-analyses were limited by the quality and methodological variability of studies available. There was considerable variation in criteria for risk stratification and the gestational age at which the intervention was introduced across the trials. The inclusion of studies using different GDM diagnostic criteria may also have contributed to heterogeneity between studies. Due to the limited number of studies in women with multiple risk factors, a subgroup analysis comprising other types of intervention (e.g., diet only, PA only, dietary supplements, preconception interventions) could not be performed. Potential publication bias for combined diet and PA intervention effects was found. Therefore, the interpretation of the findings is limited by the possible bias from selective reporting. Moreover, exclusion of non-English studies may contribute to publication bias.

### Recommendations for further research and practice

Further large-scale studies are needed, with higher methodological quality in women with multiple risk factors for GDM to determine if interventions, whether behavioural, dietary supplement or pharmacological, are more effective in reducing GDM and improving other related pregnancy outcomes than unselected population-based approaches or a single risk factor strategy. Future studies in the preconception period should consider risk stratification to identify women who may derive greater benefit. We report here that a variety of strategies were used to identify women at risk of GDM. Validation of groups of risk factors or predictive tools to identify high-risk populations should therefore be considered to harmonise risk assessment and develop effective preventative interventions to improve maternal and infant health in those women who would benefit the most.

## Conclusion

This study suggests that identification of women at high-risk of developing GDM in early pregnancy and targeted intervention using combined diet and physical activity, inositol or vitamin D reduces GDM, indicating that a targeted approach provides a promising strategy. The results should be interpreted with caution due to differences in risk stratification strategies, diagnostic criteria for GDM, gestational age for intervention, and in intervention design. Further RCTs using validated prediction tools or multiple risk factors to target high-risk women for interventions before and during pregnancy are required.

### Supplementary Information

**Additional file 1: Table A1.** Literature search strategy (MEDLINE). **Table A2.** Literature search strategy (EMBASE). **Table A3.** Literature search strategy (Cochrane Library). **Table A4.** Characteristics of the included studies. **Table A5.** Summary of criteria used for GDM risk stratification and intervention characteristic. **Table A6.** Pregnancy outcomes. **Table A7.** Eggers test of publication bias for antenatal interventions. **Figure A1.** Funnel plots of publication bias for antenatal interventions.

## Data Availability

All data generated or analysed during this study are included in this published article and its supplementary information files.

## Abbreviations

BMI: Body mass index
DCI: D-chiro-inositol
DCI-IPG: D-chiro-inositol-containing inositolphosphoglycan
FBG: Fasting blood glucose
GDM: Gestational diabetes mellitus
HbA1c: Haemoglobin a1c
LGA: Large-for-gestational age
OGTT: Oral glucose tolerance test
PA: Physical activity
PCOS: Polycystic ovary syndrome
PIR: Pregestational insulin resistance
PRISMA: Preferred reporting items for systematic reviews and meta-analyses
RCTs: Randomised controlled trials
RoB 2: The Cochrane risk-of-bias tool for randomised trials
SGA: Small for gestational age
T2DM: Type 2 diabetes mellitus
