# Supplementary material for: Interventions in preconception and pregnant women at risk of gestational diabetes; a systematic review and meta-analysis of randomised controlled trials
Source: Diabetol Metab Syndr. 2024 Jan 4;16:8. doi: 10.1186/s13098-023-01217-4 (PMC10765912; doi:10.1186/s13098-023-01217-4)
Supplement: Supplementary file 1 — Additional file 1: Table A1. Literature search strategy (MEDLINE). Table A2. Literature search strategy (EMBASE). Table A3. Literature search strategy (Cochrane Library). Table A4. Characteristics of the included studies. Table A5. Summary of criteria used for GDM risk stratification and intervention characteristic. Table A6. Pregnancy outcomes. Table A7. Eggers test of publication bias for antenatal interventions. Figure A1. Funnel plots of publication bias for antenatal interventions. [file 13098_2023_1217_MOESM1_ESM.docx]

Targeted Intervention in Preconception and Pregnant Women at Risk of Gestational Diabetes; a Systematic Review and Meta-analysis of Randomised Controlled Trials

Additional file (Appendix 1)

Table A1: Literature search strategy (MEDLINE)

Table A2: Literature search strategy (EMBASE)

Table A3: Literature search strategy (Cochrane Library)

Table A4: Characteristics of the included studies

Table A5: Summary of criteria used for GDM risk stratification and intervention characteristic

Table A6: Pregnancy outcomes

Table A7: Eggers test of publication bias for antenatal interventions

Figure A1: Funnel plots of publication bias for antenatal interventions

**Table A1:** Literature search strategy **(**MEDLINE 1946 to February, 2023)

| - - 1. Pregnancy/ |
| --- |
| - - 1. Pregnan*.tw. |
| - - 1. Gravidity/ |
| - - 1. Gravid*.tw. |
| - - 1. Gestation*.tw. |
| - - 1. Pregnant women/ |
| - - 1. Pregnant wom#n.tw. |
| - - 1. Prepregnan*.tw. |
| - - 1. Preconception*.tw. |
| - - 1. (child adj3 bearing).tw. |
| - - 1. Childbearing.tw. |
| - - 1. Matern*.tw. |
| - - 1. or/1-12 |
| - - 1. Weight gain/ph [Physiology] |
| - - 1. Weight gain*.tw. |
| - - 1. Weight loss/ph [Physiology] |
| - - 1. Weight loss*.tw. |
| - - 1. Weight change*.tw. |
| - - 1. Obesity/dh, me, ph, pc, px, th [Diet Therapy, Metabolism, Physiology, Prevention & Control, Psychology, Therapy] |
| - - 1. Obes*.tw. |
| - - 1. Adiposity/ph |
| - - 1. Adipos*.tw. |
| - - 1. Overweight/dh, me, ph, pc, px, th [Diet Therapy, Metabolism, Physiology, Prevention & Control, Psychology, Therapy] |
| - - 1. Overweight*.tw. |
| - - 1. Body mass index/ |
| - - 1. Bmi.tw. |
| - - 1. or/14-26 |
| - - 1. Exp randomized controlled trial/ |
| - - 1. "randomized controlled trial".pt. |
| - - 1. "controlled clinical trial".pt. |
| - - 1. (random$ or placebo$).tw,sh. |
| - - 1. ((singl$ or double$ or triple$ or treble$) and (blind$ or mask$)).tw,sh. |
| - - 1. Single-blind method/ |
| - - 1. Double-blind method/ |
| - - 1. or/28-34 |
| - - 1. 13 and 27 and 35 |
| - - 1. Exp animals/ |
| - - 1. (rat$ or mouse or mice or hamster$ or animal$ or dog$ or cat$ or bovine or sheep or lamb$).af. |
| - - 1. 37 or 38 |
| - - 1. Humans/ |
| - - 1. Human$.tw,ot,kf. |
| - - 1. 40 or 41 |
| - - 1. 39 not 42 |
| - - 1. 36 not 43 |

**Table A2:** Literature search strategy **(**EMBASE 1946 to February, 2023)

| 1. Pregnancy/ |
| --- |
| 1. Pregnan*.tw. |
| 1. Gravidity/ |
| 1. Gravid*.tw. |
| 1. Gestation*.tw. |
| 1. Pregnant women/ |
| 1. Pregnant wom#n.tw. |
| 1. Prepregnan/ |
| 1. Prepregnan*.tw. |
| 1. Preconception/ |
| 1. Preconception*.tw. |
| 1. (child adj3 bearing).tw. |
| 1. Childbearing.tw. |
| 1. Matern*.tw. |
| 1. or/1-14 |
| 1. Weight gain/ |
| 1. Weight gain*.tw. |
| 1. Weight loss/ |
| 1. Weight loss*.tw. |
| 1. Weight change*.tw. |
| 1. Exp obesity/ |
| 1. Obes*.tw. |
| 1. Adipos*.tw. |
| 1. Overweight*.tw. |
| 1. Body mass index/ |
| 1. Bmi.tw. |
| 1. or/16-26 |
| 1. Clinical trial/ |
| 1. Randomized controlled trial/ |
| 1. Controlled clinical trial/ |
| 1. Multicenter study/ |
| 1. Phase 3 clinical trial/ |
| 1. Phase 4 clinical trial/ |
| 1. Exp randomization/ |
| 1. Single blind procedure/ |
| 1. Double blind procedure/ |
| 1. Crossover procedure/ |
| 1. Placebo/ |
| 1. Randomi?ed controlled trial$.tw. |
| 1. RCT.tw. |
| 1. (random$ adj2 allocat$).tw. |
| 1. Single blind$.tw. |
| 1. Double blind$.tw. |
| 1. ((treble or triple) adj blind$).tw. |
| 1. Placebo$.tw. |
| 1. Prospective study/ |
| 1. or/28-46 |
| 1. Exp animal/ not human.sh. |
| 1. 47 not 48 |
| 1. 15 and 27 and 49 |

**Table A3:** Literature search strategy (Cochrane Library to February, 2023)

| #1 Pregnan* |
| --- |
| #2 MeSH descriptor: [Pregnancy] explode all trees |
| #3 Gestation |
| #4 Pregnant women |
| #5 MeSH descriptor: [Pregnant Women] explode all trees |
| #6 MeSH descriptor: [Gravidity] explode all trees |
| #7 Prepregnan* |
| #8 Preconception or periconception |
| #9 Gravidity |
| #10 #1 OR #2 OR #3 OR #4 OR #5 OR #6 OR #7 OR #8 OR #9 |
| #11 Weight gain |
| #12 Body mass index |
| #13 MeSH descriptor: [Body Mass Index] explode all trees |
| #14 BMI |
| #15 Obes* |
| #16 MeSH descriptor: [Obesity] explode all trees |
| #17 Overweight |
| #18 MeSH descriptor: [Overweight] explode all trees |
| #19 #11 OR #12 OR #13 OR #14 OR #15 OR #16 OR #17 OR #18 |
| #20 Diet |
| #21 MeSH descriptor: [Diet] explode all trees |
| #22 Lifestyle |
| #23 MeSH descriptor: [Lifestyle] explode all trees |
| #24 Exercise |
| #25 MeSH descriptor: [Exercise] explode all trees |
| #26 Physical activity |
| #27 Pharmacotherapy |
| #28 Metformin |
| #29 MeSH descriptor: [Metformin] explode all trees |
| #30 Intervention |
| #31 #20 OR #21 OR #22 OR #23 OR #24 OR #25 OR #26 OR #27 OR #28 OR #29 OR #30 |
| #32 MeSH descriptor: [Animals] explode all trees |
| #33 MeSH descriptor: [Humans] explode all trees |
| #34 #32 NOT #33 |
| #35 #10 AND #19 AND #31 |
| #36 #35 NOT 34 with Cochrane Library publication date Between Feb 2022 and Feb 2023, in Trials |

**Table A4.** Characteristics of the included studies

| Reference | Country | Design | Aims | Sample size | Age (years) | BMI (kg/m^2^) | Outcome measures |
| --- | --- | --- | --- | --- | --- | --- | --- |
| Ajmani and Sircar (2020)[1] | India | RCT | To determine the role of vitamin D in preventing GDM in high-risk pregnant women. | N= 178  I= 86  C= 92 | The predominant age was 21**–**25 years in both groups. | **Baseline BMI N (%)**  **≥25.0:**  I= 24 (27.91%)  C= 37 (40.22%) | Primary outcome: GDM. |
| Amaefule *et al.* (2022)[2] | United Kingdom | Multicentre pilot-RCT | To evaluate the feasibility/ acceptability of the intervention and to investigate the impacts of the MI on glycaemic, maternal, perinatal, cost and quality of life outcomes. | N= 198  I= 99  C= 99 | I= 31.4 ± 5.8  C= 31.9 ± 5.6 | **Baseline BMI:**  I= 28.2 ± 6.4  C= 27.9 ± 5.6 | Primary outcomes: feasibility, adherence and follow-up rates.  Secondary outcomes: GDM, intervention acceptability, glycaemia, maternal and perinatal morbidity/mortality, economic and process outcomes. |
| Al Wattar *et al.* (2019)[3] | United Kingdom | Multicentre-RCT | To evaluate the effect of a Mediterranean-style diet in reducing adverse pregnancy outcomes in higher-risk pregnant women. | N= 1252  I= 627  C= 625 | I= 31.4 ± 5.2  C= 30.9 ± 5.2 | **Baseline BMI N (%)**  **Recommended** **(18.5–24.9):**  I= 84 (14.2%)  C= 84 (13.7%)  **Overweight** **(25.0–29.9):**  I= 99 (16.7%)  C= 102 (16.7%)  **Obese** **(30.0–39.9):**  I= 410 (69.1%)  C= 426 (69.6%) | Primary outcomes: composite maternal (GDM or pre-eclampsia) and composite offspring (stillbirth, SGA or NICU admission).  Secondary outcomes: GDM, pre-eclampsia, GWG, admission to HDU or ICU, antepartum haemorrhage, mode of delivery, preterm delivery, anaemia, stillbirth, neonatal death, SGA, NICU admission, birthweight, hypoxic ischaemic encephalopathy. |
| Basu *et al*. (2021)[4] | United States | RCT | To examine the impact of combined dietary supplementation (whole blueberries/ soluble fibre) on cardiometabolic profiles in minority women at greater risk of GDM. | N= 45  I= 22  C= 23 | I= 27 ± 5.3  C= 27 ± 5.0 | **Baseline BMI**  I= 35 ± 4.2  C= 36 ± 4.2 | Primary outcomes: GWG, cardiometabolic profiles.  Secondary outcomes: GDM, dietary intake. |
| Begum *et al*. (2009)[5] | Bangladesh | RCT | To evaluate the effect of metformin throughout pregnancy on reducing the development of GDM in women with PCOS. | N= 59  I= 29  C= 30 | I= 28.14 ± 2.92  C= 26.13 ± 3.62 | **Baseline BMI**  I= 28.21 ± 2.37  C= 27.97 ± 2.49 | Primary outcomes: GDM, abortion rate, Apgar score, birthweight, macrosomia, preterm delivery, birth asphyxia, neonatal death. |
| Bisson *et al*. (2015)[6] | Canada | Preliminary-RCT | To assess whether a 12-week supervised exercise program promotes an active lifestyle throughout pregnancy in women with obesity. | N= 50  I= 25  C= 25 | I= 30.5 ± 3.7  C= 31.0 ± 4.0 | **Pre-pregnancy BMI**  I= 34.6 ± 5.4  C= 33.9 ± 4.5  **Pre-pregnancy BMI N (%)**  **Obesity class I (30.0-34.9)**:  I= 17 (68%)  C= 16 (64%)  **Obesity class II (35.0-39.9):**  I= 3 (12%)  C= 7 (28%)  **Obesity class III (≥ 40.0):**  I= 5 (20%)  C=2 (8%) | Primary outcome: the time spent at moderate and vigorous physical activity.  Other perinatal and neonatal outcomes: GDM, birthweight, PIH, c-section. |
| Brink *et al*. (2018)[7] | Netherlands | RCT | To investigate the impact of metformin in pregnant women with obesity and other risk factors for GDM. | N= 49  I= 24  C= 25 | I= 29.3 ± 5.2  C= 30.7 ± 5.2 | **Baseline BMI**  I= 31.3 ± 5.8  C= 30.0 ± 5.5 | Primary outcome: GDM.  Other outcomes: insulin therapy, pre-eclampsia, c-section, miscarriage, birthweight, preterm delivery, LGA, birth trauma, respiratory distress, NICU admission, neonatal hypoglycaemia, hyperbilirubinemia, requiring phototherapy. |
| Bogaerts *et al*. (2013)[8] | Belgium | Multicentre-RCT | To investigate the impact of a prenatal lifestyle intervention program in reducing GWG and lowering anxiety levels and depressed mood in pregnant women with obesity. | N= 197  Brochure I= 58  Lifestyle I= 76  C= 63 | Brochure I= 29.6 ± 4.9  Lifestyle I= 28.8 ± 4.5  C= 28.7 ± 4.2 | **Pre-pregnancy BMI**  Brochure I= 35.4 ± 5.2  Lifestyle I= 34.4 ± 4.6  C= 34.4 ± 4.1 | Primary outcome: GWG.  Secondary outcomes: anxiety levels and depressed mood.  Other perinatal and neonatal outcomes: GDM, birthweight, PIH, mode of delivery. |
| Bruno *et al.* (2017)[9] | Italy | RCT | To determine the effect of diet and physical activity-based program on the occurrence of GDM in women who are overweight or obese. | N= 191  I= 96  C= 95 | I= 31.5 ± 5  C= 30.8 ± 5.5 | **Pre-pregnancy BMI**  I= 33.3 ± 6.0  C= 33.4 ± 5.5  **Baseline BMI**  I= 33.9 ± 5.7  C= 34.5 ± 6.8  **Baseline BMI N (%)**  **Obese:**  I= 63 (65.6%)  C= 69 (72.6%) | Primary outcome: GDM.  Secondary outcomes: GWG, adoption of healthy nutrition practices, PIH, mode of delivery, preterm delivery, birthweight, neonatal hypoglycaemia, NICU admission, Apgar score, need for resuscitation. |
| Callaway *et al*. (2010)[10] | Australia | Pilot-RCT | To evaluate the feasibility of an individualised, goal-directed exercise intervention in pregnant women with obesity. | N= 50  I= 25  C= 25 | I= 30.4 ± 4.8  C= 30.0 ± 5.9 | **Baseline BMI N (%)**  **≥ 35.0**  I= 9 (36%)  C= 9 (36%) | Primary outcome: energy expenditure.  Secondary outcomes: GDM, fasting insulin, fasting glucose, insulin resistance. |
| Callaway *et al*. (2019)[11] | Australia | RCT | To determine whether probiotics given from the 2^nd^ trimester in women who are overweight and obese prevent GDM as assessed by an OGTT at 28 weeks’ gestation. | N= 411  I= 207  C= 204 | I= 31.3 ± 4.7  C= 31.7 ± 4.8  **Age ≥ 35y N (%)**  I= 54 (26.1%)  C= 57 (27.9%) | **Baseline BMI**  I= 31.9 ± 7.5  C= 31.6 ± 7.2  **Baseline BMI N (%)**  **Overweight (25.0–29.9):**  I= 66 (31.9%)  C= 76 (37.3%)  **Obese (30.0–39.9):**  I= 114 (55.1%)  C= 101 (49.5%)  **Highly morbidly Obese (≥ 40.0):**  I= 27 (13.0%)  C= 27 (13.2%) | Primary outcome: GDM.  Secondary outcomes: GWG, pre-eclampsia, hypertensive disorders of pregnancy, c-section, preterm delivery, NICU admission, jaundice, neonatal hypoglycaemia, birthweight, SGA, LGA, stillbirth, birth injury. |
| Celentano *et al*. (2018)[12] | Italy | RCT | To determine the impact of different inositol stereoisomers on insulin resistance and GDM development in women at high-risk of the condition. | N= 180  MI= 40  DCI= 40  MI/DCI= 40  C= 60 | MI= 33.1 ± 4.9  DCI= 34.4 ± 3.7  MI/DCI= 34.1 ± 4.2  C= 33.9 ± 4.9 | **Pre-pregnancy BMI**  MI= 23.5 ± 3.4  DCI= 24.4 ± 4.9  MI/DCI= 23.5 ± 4.6  C= 24.4 ± 4.1 | Primary outcome: GDM.  Secondary outcomes: maternal BMI, insulin therapy, fetal growth, polyhydramnios, pre-eclampsia, PIH, c-section, preterm delivery, birthweight, neonatal hypoglycaemia, NICU admission. |
| Chan *et al*. (2018)[13] | China | Parallel-RCT | To compare the effect of a lifestyle intervention in early pregnancy with routine antenatal care in reducing the incidence of GDM, the proportion of LGA infants and macrosomia and improving other maternal and birth outcomes in pregnant women at risk of GDM. | N= 229  I= 118  C=111 | I= 33.2 ± 4.4  C= 33.1 ± 4.1 | **Pre-pregnancy BMI**  I= 22.6 ± 3.8  C= 23.4 ± 3.9  **Baseline BMI**  I= 23.1 ± 3.9  C= 24.1 ± 4.0 | Primary outcome: GDM.  Secondary outcomes: LGA, macrosomia.  Tertiary outcomes: pre-eclampsia, PIH, c-section, preterm delivery, Apgar score, birthweight, SGA, LGA, macrosomia, shoulder dystocia. |
| Chiswick *et al.* (2015)[14] | United Kingdom | Multicentre-RCT | To test whether metformin would reduce birthweight in pregnant women with obesity. | N= 449  I= 226  C= 223 | I= 28.7 ± 5.8  C= 28.9 ± 5.1 | **Baseline BMI:**  I= 37.8 ± 4.9  C= 37.7 ± 5.6 | Primary outcome: birthweight.  Secondary outcomes: GDM, insulin resistance, fasting glucose/insulin concentrations, maternal/newborn anthropometrics, birthweight, NICU admission, congenital anomaly. |
| Corcoy *et al*. (2020)[15] | Across seven European countries (United Kingdom, Republic of Ireland, Austria, Poland, Italy, Spain and Belgium) | Multicentre-RCT | To test if vitamin D supplementation could reduce GDM risk (evaluated after FPG, insulin resistance and weight gain) in pregnant women who are overweight/obese. | N= 154  I= 79  C= 75 | I= 32.2 ± 5.2  C= 32.8 ± 5.4 | **Pre-pregnancy BMI**  I= 33.7 ± 4.3  C= 33.3 ± 4.3  **Baseline BMI:**  I= 34.2 ± 4.3  C= 34.2 ± 4.2 | Primary outcomes: FPG, insulin resistance, GWG.  Secondary outcomes: GDM, glucose/insulin measurements, birthweight, SGA. |
| D’Anna *et al*. (2013)[16] | Italy | RCT | To test if MI supplementation can reduce the onset of GDM in pregnant women with a family history of type 2 diabetes. | N= 197  I= 99  C= 98 | I= 31.0 ± 5.3  C= 31.6 ± 5.6 | **Pre-pregnancy BMI**  I= 22.8 ± 3.1  C= 23.6 ± 3.1 | Primary outcome: GDM.  Secondary outcomes: macrosomia, PIH, preterm delivery, c-section, shoulder dystocia, neonatal hypoglycaemia, respiratory distress syndrome. |
| D’Anna *et al.* (2015)[17] | Italy | RCT | To determine the effectiveness of MI supplementation in reducing GDM incidence, insulin resistance and related complications among pregnant women with obesity. | N= 220  I= 110  C= 110 | I= 30.9 (18–44)  C= 31.7 (19–43) | **Pre-Pregnancy BMI**  I= 33.8 (30.0–46.9)  C= 33.8 (30.0–46.0) | Primary outcomes: GDM, insulin resistance.  Secondary outcomes: c-section, hypertensive disorders, preterm delivery, shoulder dystocia, macrosomia, neonatal hypoglycaemia, NICU admission. |
| Daly *et al*. (2017)[18] | Republic of Ireland | RCT | To evaluate whether an intensive medically supervised exercise intervention for pregnant women with obesity (BMI ≥ 30 kg/m^2^) reduces the mean FPG levels by 6.9 mg/dL (0.4 mmol/L) at 24–28 weeks of gestation compared to women receiving routine prenatal care alone. | N= 88  I= 44  C= 44 | I= 30.0 ± 5.1  C= 29.4 ± 4.8 | **Baseline BMI:**  I= 34.7 ± 4.6  C= 34.7 ± 5.1 | Primary outcome: FPG.  Secondary outcomes: GDM, FPG, GWG, birth outcomes, induction of labour, mode of delivery, length of labour, birthweight, gestational age at delivery, preterm delivery, NICU admission, Apgar score. |
| Deng *et al.* (2021)[19] | China | RCT | To determine whether diet and exercise-based interventions improve the incidence of GDM, GWG and pregnancy outcomes in women with high-risk factors. | N= 94  I= 47  C= 47 | I= 29.6 ± 3.9  C= 29.6 ±3.4 | **Pre-pregnancy BMI**  I= 24.6 ± 3.4  C= 25.2 ± 2.9  **Pre-pregnancy BMI N (%)**  **Normal (<24.0):**  I= 20 (42.6%)  C= 16 (34.0%)  **Overweight (24.0-28.0):**  I= 20 (42.6%)  C= 25 (53.2%)  **Obesity (≥28.0):**  I= 7 (14.9%)  C= 6 (12.8%) | Primary outcome: GDM.  Secondary outcomes: GWG, c-section, PIH, preterm delivery, macrosomia. |
| Ding *et al*. (2021)[20] | China | RCT | To assess the effect of a WeChat-based dietary and exercise intervention for preventing GDM. | N= 215  I= 104  C= 111 | I= 30.6 ± 2.8  C= 30.1 ± 2.7 | **Baseline BMI**  I= 27.7 ± 2.6  C= 27.9 ± 2.9  **Baseline BMI N (%)**  **24.0-27.9:**  I= 70 (67.3%)  C= 67 (60.4%)  **≥28.0:**  I= 34 (32.7%)  C= 44 (39.6%) | Primary outcome: GDM.  Secondary outcomes: GWG, c-section, preterm delivery, postpartum haemorrhage, PROM, PIH, pre-eclampsia, macrosomia, birthweight, fetal distress, amniotic fluid contamination. |
| Dodd *et al*. (2019)[21] | Australia | Multicentre-RCT | To evaluate the impact of antenatal metformin given in addition to dietary and lifestyle interventions on maternal and infant outcomes amongst pregnant women who are overweight and obese. | N= 524  I= 261  C= 263 | I= 29.9 ± 5.5  C= 30.2 ± 5.4 | **Baseline BMI:**  I= 32.5 (28.7-37.5)  C= 32.0 (29.1- 36.8)  **Baseline BMI N (%)**  **25.0-29.9:**  I= 83 (32)  C= 84 (33)  **≥ 30.0:**  I= 173 (68)  C= 174 (67) | Primary outcome: LGA.  Secondary outcomes: GDM, GWG, PIH, pre-eclampsia, antepartum haemorrhage, PROM, chorioamnionitis, induction of labour, c-section, postpartum haemorrhage, perineal trauma, wound infection, endometritis, thromboembolic disease, antenatal admission, antibiotic use during delivery, maternal death, maternal quality of life, maternal diet and physical activity, preterm birth, perinatal mortality, birthweight, LGA, SGA, neonatal hypoglycaemia, NICU admission, hyperbilirubinemia, nerve palsy, fracture, birth trauma, shoulder dystocia, newborn anthropometrics. |
| Dodd *et al*. (2014)[22] | Australia | Multicentre-RCT | To determine the impact of antenatal dietary and lifestyle interventions on health outcomes among pregnant women who are overweight and obese. | N= 2122  I= 1118  C= 1104 | I= 29.3 ± 5.4  C= 29.6 ± 5.6 | **Baseline BMI**  I= 31.0 (28.1-35.9)  C= 31.1 (27.7- 35.6)  **Baseline BMI N (%)**  **25.0-29.9:**  I= 458 (41.4%)  C= 468 (42.7%)  **30.0- 34.9:**  I= 326 (29.5%)  C= 318 (29.0%)  **35.0-39.9:**  I= 202 (18.3%)  C= 183 (16.7%)  **≥ 40.0:**  I= 119 (10.8%)  C= 128 (11.7%) | Primary outcome: LGA.  Secondary outcomes: GDM, GWG, PIH, pre-eclampsia, antepartum haemorrhage, PROM, chorioamnionitis, induction of labour, c-section, postpartum haemorrhage, perineal trauma, wound infection, endometritis, thromboembolic disease, antenatal admission, antibiotic use during delivery, maternal death, preterm delivery, perinatal mortality, neonatal death, birthweight, neonatal hypoglycaemia, NICU admission, hyperbilirubinemia, nerve palsy, fracture, birth trauma, shoulder dystocia. |
| Eslami *et al*. (2018)[23] | Iran | RCT | To determine the effect of a lifestyle training program on GWG and GDM rate among pregnant women who are obese or overweight. | N= 140  I= 70  C= 70 | **<20:**  I= 7(10.0) C= 4 (5.7)  **20 – 30:**  I= 46 (65.7) C= 39 (55.7)  **>30:**  I= 17 (24.3) C=27 (38.6) | **Baseline BMI N (%)**  **25.0-30.0:**  I= 51 (72.9%)  C= 51 (72.9%)  **≥ 30.0:**  I= 19 (27.1%)  C= 19 (27.1%) | Primary outcomes: diet and physical activity.  Secondary outcomes: GDM, GWG. |
| Esmaeilzadeh *et al*. (2022)[24] | Iran | RCT | To investigate if a low dosage of MI supplementation might decrease GDM rate in pregnant women who are overweight. | N= 60  I= 30  C= 30 | **Age ≥ 30 years**  I= 11 (40.7)  C= 16 (55.2) | **Pre-pregnancy BMI**  I= 27.3 ± 1.8  C= 26.9 ± 1.9 | Primary outcome: GDM.  Secondary outcomes: GWG, PIH, insulin therapy, insulin resistance, lipid profile, preterm delivery, c-section, macrosomia, NICU admission, shoulder dystocia respiratory distress. |
| Farren *et al*. (2017)[25] | Republic of Ireland | RCT | To investigate if the use of inositol in combination of MI/DCI could prevent GDM in pregnant women with a family history of diabetes. | N= 240  I= 120  C= 120 | I= 31.1 ± 5.1  C= 31.5 ± 5.0 | **Baseline BMI**  I= 26.0 ± 5.3  C= 26.2 ± 5.5 | Primary outcome: GDM.  Secondary outcomes: pre-eclampsia, PIH, induction of labour, mode of delivery, perineal trauma, birthweight, shoulder dystocia, brachial plexus palsy, NICU admission, neonatal hypoglycaemia, respiratory distress syndrome. |
| Ferrara *et al*. (2020)[26] | United States | Multicentre-RCT | To reduce excess GWG using a telehealth behavioural lifestyle intervention adapted from the DPP. | N= 398  I= 200  C= 198 | I= 32.4 ± 4.1  C= 32.6 ± 4.3 | **Pre-pregnancy BMI**  I= 29.3 ± 3.4  C= 29.5 ± 3.8  **Pre-pregnancy BMI N (%)**  **25.0-29.9:**  I= 131 (66%)  C= 124 (64%)  **30.0-40.0:**  I= 68 (34%)  C= 71 (36%) | Primary outcome: GWG.  Secondary outcomes: GDM, GWG, diet and physical activity, metabolic markers, birthweight, macrosomia, pregnancy loss, preterm delivery, c-section, PIH, pre-eclampsia. |
| Gallagher *et al*. (2018)[27] | United States | RCT | To examine the effect of a lifestyle intervention designed to control GWG on fat and fat-free mass in infants. | N= 210  I= 105  C= 105 | I= 33.8 ± 4.0  C= 33.8 ± 4.7 | **Baseline BMI**  I= 30.1 ± 4.1  C= 30.7 ± 5.0  **Baseline BMI N (%)**  **Overweight (25.0–29.9):**  I= 65 (62%)  C= 60 (57%)  **Obese (>30.0):**  I= 40 (38%)  C= 45 (43%) | Maternal outcomes: GDM, GWG, mode of delivery, preterm delivery, diet.  Infant outcomes: body composition, LGA, SGA, birthweight. |
| Guelfi *et al*. (2016)[28] | Australia | RCT | To examine the impact of a 14-week supervised, home-based exercise intervention on the recurrence of GDM. | N= 172  I= 85  C= 87 | I= 33.6 ± 4.1  C= 33.8 ± 3.9 | **Baseline BMI N (%)**  **≤.24.9:**  I= 37 (44%)  C= 48 (55%)  **25.0–29.9**:  I= 30 (35%)  C= 19 (22%)  **≥30.0:**  I= 20 (23%)  C= 18 (21%) | Primary outcome: GDM.  Secondary outcomes: maternal fitness, psychological well-being, mode of delivery, pre-eclampsia, postpartum haemorrhage, sepsis, preterm delivery, Apgar score, birthweight LGA, SGA. |
| Garnæs *et al*. (2016)[29] | Norway | RCT | To investigate if an exercise program can reduce GWG and pregnancy outcomes such as GDM and high blood pressure among pregnant women who are overweight or obese. | N= 91  I= 46  C= 45 | I= 31.3 ± 3.8  C= 31.4 ± 4.7 | **Baseline BMI**  I= 33.9 ± 3.8  C= 35.1 ± 4.6  **Baseline BMI N (%)**  **Overweight (28.0-29.9):**  I= 3 (6.6%)  C= 5 (11.1%)  **Obesity class I (30.0–34.9)**:  I= 28 (62.2%)  C= 19 (42.2%)  **Obesity class II (35.0–39.9):**  I= 11 (24.4%)  C= 15 (33.3%)  **Obesity class III (≥ 40.0):**  I= 3 (6.6%)  C= 6 (13.3%) | Primary outcome: GWG.  Secondary outcomes: GDM, BMI, body composition, physical activity, skinfold thickness, blood pressure, various blood tests, PIH. |
| Gonzalez-Plaza *et al*. (2022)[30] | Spain | RCT | To assess the efficacy of a complex digital health intervention on GWG, physical activity and maternal and perinatal outcomes in pregnant women who are overweight or obese. | N= 150  I= 78  C= 72 | I= 32.4 ± 5.4  C= 33.4 ± 4.7 | **Pre-pregnancy BMI**  I= 33.1 ± 2.9  C= 32.7 ± 3.3  **Baseline BMI N (%)**  **Obesity class I (30.0–34.9)**:  I= 63 (81%)  C= 61 (85%)  **Obesity class II (35.0–39.9):**  I= 13 (17%)  C= 8 (11%)  **Obesity class III (≥ 40.0):**  I= 2 (2%)  C= 3 (4%) | Primary outcomes: GWG, physical activity.  Secondary outcomes: GDM, pre-eclampsia, PIH, miscarriage, mode of delivery, c-section, preterm delivery, NICU admission, birthweight, macrosomia, LGA, SGA. |
| Halkjær *et al*. (2020)[31] | Denmark | RCT | To examine the effect of probiotics on GWG, HbA1c and GDM. | N= 50  I= 25  C= 25 | I= 30.7 ± 4.5  C= 30.7 ± 4.7 | **Pre-pregnancy BMI**  I= 31.7 ± 1.8  C= 32.1 ± 1.3 | Primary outcomes: GDM, GWG, HbA1c levels.  Secondary outcomes: birthweight, maternal gut microbiota diversity. |
| Harrison *et al*. (2013)[32] | Australia | RCT | To optimise lifestyle and GWG in early pregnancy in women at increased risk of developing GDM. | N= 228  I= 121  C= 107 | I= 32.4 ± 4.6  C= 31.7 ± 4.5 | **Baseline BMI**  I= 30.4 ± 5.6  C= 30.3 ± 5.9  **Baseline BMI N (%)**  **≤ 29.9:**  I= 69 (57%)  C= 64 (60%)  **≥ 30.0:**  I= 52 (43%)  C= 43 (40%) | Primary outcome: GWG.  Secondary outcomes: GDM, physical activity, risk perception. |
| Herring *et al*. (2015)[33] | United States | RCT | To assess if a technology-based behavioural intervention can reduce the proportion of African American women with overweight/obesity who had GWG above the IOM recommendations. | N= 66  I= 33  C= 33 | I= 25.9 ± 4.9  C= 25.0 ± 5.7 | **Baseline BMI**  I= 33.5 ± 5.8  C= 32.2 ± 5.4  **Baseline BMI N (%)**  **25.0–29.9:**  I= 13 (39%)  C= 11 (33%)  **30.0– 45.0:**  I= 20 (61%)  C= 22 (67%) | Primary outcome: GWG.  Secondary outcomes: GDM, mode of delivery, birthweight, SGA, LGA. |
| Jamal, Milani and Al-Yasin. (2012)[34] | Iran | RCT | To assess the impact of metformin on the uteroplacental circulation compared with aspirin and determine pregnancy complications in pregnant women with PCOS. | N= 105  Metformin I= 35  Aspirin I= 35  C= 35 | NA. | NA. | Primary outcome: uterine artery pulsatility index.  Secondary outcomes: GDM, pre-eclampsia, preterm delivery, birthweight, IUGR. |
| Kennelly *et al*. (2018)[35] | Republic of Ireland | RCT | To evaluate the impact of an antenatal lifestyle intervention using smartphone application technology on GDM rates in women who are overweight or obese. | N= 565  I= 278  C= 287 | I= 32.8 ± 4.6  C= 32.1 ± 4.2 | **Baseline BMI**  I= 29.4 ± 3.6  C= 29.1 ± 3.3  **Baseline BMI N (%)**  **Overweight (25.0-29.9):**  I= 182 (65.5%)  C= 194 (67.6%)  **Obesity class I (30.0–34.9)**:  I= 68 (24.4%)  C= 67 (23.3%)  **Obesity class II (35.0–39.9):**  I= 28 (10.1%)  C= 26 (9.1%) | Primary outcome: GDM.  Secondary outcomes: GWG, glycaemic index, glycaemic load, exercise levels, birthweight, LGA. |
| Koivusalo *et al.* (2016)[36] | Finland | Multicentre-RCT | To assess the effectiveness of moderate lifestyle intervention in preventing GDM in pregnant women who are at high-risk for the disorder. | N= 293  I= 155  C= 138 | I= 32.3 ± 4.9  C= 32.6 ± 4.5 | **Pre-pregnancy BMI**  I= 31.5 ± 6.0  C= 32.0 ± 5.5  **Baseline BMI**  I= 32.2 ± 5.9  C= 32.3 ± 5.4 | Primary outcome: GDM.  Secondary outcomes: FPG, pre-eclampsia, PIH, GWG, mode of delivery. |
| Korpi-Hyovalti *et al.* (2011)[37] | Finland | Multicentre-RCT | To evaluate the feasibility of an early pregnancy lifestyle intervention aiming to improve glucose tolerance in women at a high-risk for GDM. | N= 54  I= 30  C= 30 | I= 29.1 ± 5.4  C= 29.8 ± 5.4 | **Baseline BMI**  I= 27.3 ± 6.0  C= 25.5 ± 3.4  **BMI N (%)**  **> 25.0:**  I= 18 (60.0%)  C= 17 (56.7%) | Primary outcome: GDM.  Maternal outcomes: GWG, glucose intolerance, insulin therapy.  Infant outcomes: birthweight, macrosomia, gestational age, NICU admission, jaundice, respiratory distress. |
| Kong *et al.* (2014)[38] | United States | Pilot-RCT | To promote moderate-intensity physical activity (walking) among pregnant women who are overweight or obese to assess the impact of the intervention on maternal and birth outcomes. | N= 42  I= 19  C= 23 | **Overweight**  I= 26.2 ± 2.6  C= 27.3 ± 3.6  **Obese**  I= 28.6 ± 5.3  C= 25.7 ± 4.0 | **Pre-pregnancy BMI**  **Overweight:**  I= 26.5 ± 1.2  C= 27.4 ± 1.4  **Obese:**  I= 34.7 ± 4.6  C= 34.2 ± 3.6 | Primary outcome: physical activity.  Secondary outcomes: GDM, GWG, birthweight, macrosomia, Apgar score, preterm delivery, c-section, pre-eclampsia, PIH. |
| LeBlanc *et al*. (2022)[39] | United States | RCT | To determine whether a pre-pregnancy weight loss program reduces GWG and improves pregnancy outcomes. | N= 326  I= 164  C= 162 | I= 31.6 ± 3.5  C= 30.9 ± 3.5 | **Baseline BMI**  I= 34.9 ± 6.0  C= 34.7 ± 5.5  **BMI N (%)**  **27.0– 29.9:**  I= 19 (21.4%)  C= 18 (22.5%)  **30.0–34.9:**  I= 34 (38.2%)  C= 31 (38.8%)  **≥35.0:**  I= 36 (40.5%)  C= 31 (38.8%) | Primary outcome: GWG.  Secondary outcomes: GDM, PIH, pregnancy loss, preterm delivery, c-section, congenital anomalies, birthweight, LGA, SGA, neonatal hypoglycaemia. |
| Lin *et al.* (2020)[40] | China | RCT | To assess the efficacy of diet, physical activity and weight management-based intervention on the incidence of GDM and risk of adverse pregnancy outcomes among Chinese women at high-risk for GDM. | N= 304  I= 152  C= 152 | I= 31.4 ± 4.9  C= 31.8 ± 5.1 | **Pre-pregnancy BMI**  I= 25.4 ± 3.4  C= 25.9 ± 3.7 | Primary outcome: GDM.  Secondary outcomes: excessive GWG, c-section, PIH, PROM, antepartum haemorrhage and postpartum haemorrhage. |
| Liu *et al*. (2021)2[41] | United States | RCT | To assess if an antenatal behavioural lifestyle intervention can reduce GWG and perinatal outcomes. | N= 228  I= 114  C= 114 | I= 30.4 ± 5.1  C= 29.1 ± 4.8 | **Pre-pregnancy BMI**  I= 31.9 ± 5.9  C= 32.7 ± 5.9 | Primary outcome: GWG.  Secondary outcomes: GDM, PIH, c-section, preterm delivery, birthweight, macrosomia, SGA, Apgar score, hospital stay, diet and physical activity. |
| Lovvik *et al.* (2019)[42] | Norway, Sweden and Iceland. | Multicentre-RCT | To determine if metformin can prevent late miscarriage and preterm birth in pregnant women with PCOS. | N= 487  I= 244  C= 243 | I= 29 (24–34)  C= 30 (24–36) | **Baseline BMI**  I= 27.5 (18.2–36.5)  C= 26.7 (18.8–34.6) | Primary outcomes: composite incidence of late miscarriage, preterm birth.  Secondary outcomes: GDM, PIH, pre-eclampsia, NICU admission. |
| Luoto *et al.* (2011)[43] | Finland | Cluster-RCT | To investigate the effect of lifestyle modification in preventing GDM or high birthweight among pregnant women at high-risk of GDM. | N= 442  I= 246  C= 196 | I= 29.5 ± 4.8  C= 30.0 ± 4.7 | **Pre-pregnancy BMI**  I= 26.3 ± 4.9  C= 26.4 ± 4.3 | Primary outcomes: GDM, birthweight.  Secondary outcomes: GWG, insulin therapy. |
| Matarrelli *et al*. (2013)[44] | Italy | Pilot-RCT | To test if dietary MI can improve insulin resistance and reduce GDM rates in women at increased risk of GDM. | N= 75  I= 36  C= 39 | I= 33.0 ± 4.9  C= 33.8 ± 4.7 | **Pre- pregnancy BMI**  I= 23.5 ± 3.4  C= 24.7 ± 4.2 | Primary outcome: GDM.  Secondary outcomes: maternal BMI, insulin therapy, macrosomia, birthweight, neonatal hypoglycaemia, glycaemic values, fetal biometry percentiles, polyhydramnios. |
| McCarthy *et al.* (2016)[45] | Australia | RCT | To determine the impact of self-weighing and dietary advice compared to standard antenatal care on obstetric outcomes. | N= 382  I= 190  C= 192 | I= 31.9 ± 4.6  C= 31.8 ± 4.6 | **Pre- pregnancy/ early pregnancy BMI**  I= 30.5 (28.2-35.0)  C= 30.1 (27.5-35.3)  **Pre- pregnancy/ early pregnancy BMI N (%)**  **25.0-29.9:**  I= 89 (46.8%)  C= 90 (46.9%)  **30.0–34.9**:  I= 52 (27.4%)  C= 51 (26.6%)  **35.0–39.9:**  I= 28 (14.7%)  C= 31 (16.1%)  **≥ 40.0:**  I= 21 (11.1%)  C= 20 (10.4%) | Primary outcome: composite of obstetric complications including GDM, PIH, pre-eclampsia, assisted birth or c-section, shoulder dystocia, severe perineal trauma, postpartum haemorrhage, maternal high dependency care.  Secondary outcomes: GWG, quality of life, leptin, adiponectin, CRP. |
| Mohsenzadeh-Ledari *et al*. (2020)[46] | Iran | RCT | To investigate the impact of a caring intervention on improving maternal complications such as GDM in pregnant women with metabolic syndrome. | N= 120  I= 60  C= 60 | I= 31.0 ± 6.0  C= 31.4 ± 5.2 | **Baseline BMI**  I= 31.3 ± 6.3  C= 28.5 ± 4.4  **Baseline BMI N (%)**  **≤ 25:**  I= 6 (10.0%)  C= 14 (23.3%)  **25.0-29.9:**  I= 25 (41.7%)  C= 28 (46.7%)  **30.0- 34.9:**  I= 15 (25.0%)  C=13 (21.7%)  **35.0-39.9:**  I= 6 (10.0%)  C= 4 (6.7%)  **≥ 40.0:**  I= 8 (13.3%)  C= 1 (1.7%) | Primary outcomes: GWG and 2-hr blood glucose.  Secondary outcomes: GDM, hospitalization due to GDM, nutritional diet for GDM. |
| Motahari-Tabari *et al.* (2021)[47] | Iran | RCT | To determine whether an information-motivation and behavioural skills model-based counselling is effective in preventing GDM in women who are overweight/obese. | N= 137  I= 70  C= 67 | I= 29.0 ± 5.9  C= 27.7 ± 5.7 | **Baseline BMI**  I= 32.4 ± 3.4  C= 33.2 ± 4.6 | Primary outcome: GDM. |
| Okesene-Gafa *et al*. (2019)[48] | New Zealand | 2X2 factorial RCT | To determine the effect of a culturally tailored dietary intervention with or without daily probiotic capsules in pregnant women with obesity on excessive GWG and birthweight. | N= 230  Diet I= 116  Routine advice C= 114  Probiotics I= 115  Placebo C= 115 | Diet I= 29.8± 5.7  Routine advice C= 27.8 ± 5.5  Probiotics I= 28.9 ± 5.7  Placebo C= 28.6 ± 5.7 | **Baseline BMI**  Diet I= 39.2 ± 6.2  Routine advice C= 37.9 ± 5.9  Probiotics I= 38.9 ± 6.5  Placebo C= 38.2 ± 5.7  **Baseline BMI N (%)**  **30.0–34.9**:  Diet I= 35 (30.2%)  Routine advice C= 38 (33.3%)  Probiotics I= 36 (31.3%)  Placebo C= 37 (32.2%)  **>35.0:**  Diet I= 81 (69.8%)  Routine advice C= 76 (66.7%)  Probiotics I= 79 (68.7%)  Placebo C= 78 (67.8%) | Primary outcomes: excessive GWG, birthweight.  Secondary outcomes: GDM, GWG, HbA1c levels, depression, anxiety, mode of delivery, newborn anthropometrics/body composition, LGA, SGA, NICU admission, composite neonatal morbidity. |
| Oostdam *et al*. (2012)[49] | Netherlands | RCT | To investigate the effectiveness of an exercise program for pregnant women at risk of GDM on maternal fasting blood glucose, insulin sensitivity and birthweight. | N= 121  I= 62  C= 59 | I= 30.8 ± 5.2  C= 30.1 ± 4.5 | **Pre-pregnancy BMI**  I= 33.0 ± 3.7  C= 33.9 ± 5.6 | Primary outcome: FPG.  Other outcomes: GDM, c-section, birthweight, LGA, fasting insulin, HbA1c, maternal weight/BMI, physical activity. |
| Parat *et al.* (2019)[50] | France | Multicentre-RCT | To determine the effect of an educational intervention during and after pregnancy on reducing postnatal infancy weight gain. | N= 275  I= 132  C= 136 | I= 30.3 ± 5.1  C= 30.5 ± 5.0 | **Pre-pregnancy BMI**  I= 32.5 ± 5.4  C= 32.5 ± 5.4 | Primary outcome: postnatal infancy weight gain.  Secondary outcomes: GDM, PIH, pre-eclampsia, c-section, need for instrumental delivery. LGA, exclusive breastfeeding, length of exclusive breastfeeding, postpartum BMI, diabetes 2 years after delivery. |
| Peccei *et al.* (2017)[51] | United States | RCT | To evaluate the impact of a culturally appropriate nutritional intervention on GWG and postpartum weight retention among pregnant women who are overweight or obese in a community health setting. | N= 300  I= 200  C= 100 | NA | NA | Primary outcome: GWG.  Secondary outcomes: GDM, hypertensive disorders, c-section, birthweight, LGA, SGA, NICU admission, postpartum weight retention. |
| Pellonperä *et al.* (2019)[52] | Finland | RCT | To evaluate the effect of daily fish oil and/or probiotic supplements on the risk of GDM and glucose metabolism in pregnant women who are overweight or obese. | N= 439  Fish oil + placebo I= 109  Probiotics + placebo I= 110  Fish oil + probiotics I= 109  Placebo + placebo C= 110 | Fish oil + placebo I= 30.4 ± 4.8  Probiotics + placebo I= 30.8 ± 4.8  Fish oil + probiotics I= 30.8 ± 4.6  Placebo + placebo C= 30.4 ± 4.1 | **Pre- pregnancy BMI**  Fish oil + placebo I= 30.0 ± 4.2  Probiotics + placebo I= 29.9 ± 4.7  Fish oil + probiotics I= 29.3 ± 3.9  Placebo + placebo C= 29.7 ± 4.2  **Pre-pregnancy BMI N (%)**  **Overweight:**  Fish oil + placebo I= 62 (56.4%)  Probiotics + placebo I= 70 (64.2%)  Fish oil + probiotics I= 68 (62.4%)  Placebo + placebo C= 66 (60.0%)  **Obese:**  Fish oil + placebo I= 48 (43.6%)  Probiotics + placebo I= 39 (35.8%)  Fish oil + probiotics I= 41 (37.6%)  Placebo + placebo C= 44 (40.0%) | Primary outcomes: GDM and FPG.  Secondary outcomes: insulin, HOMA2-IR, insulin/metformin therapy, hypertensive disorders, mode of delivery, postpartum haemorrhage, birthweight, macrosomia. |
| Petrella *et al. (*2013)[53] | Italy | RCT | To determine whether lifestyle changes in women with increased BMI can prevent GWG and improve adverse maternal-fetal outcomes. | N= 63  I= 33  C= 30 | I= 31.5 ± 4.2  C= 32.4 ± 5.9 | **Baseline BMI**  I= 32.1 ± 5.0  C= 32.9 ± 6.2  **Baseline BMI N (%)**  **25.0-29.9:**  I= 15 (45.5%)  C= 10 (33.3%)  **≥ 30.0:**  I= 18 (54.5%)  C= 20 (66.7%) | Primary outcome: excessive GWG.  Secondary outcomes: GDM, PIH, preterm delivery. |
| Phelan *et al.* (2018)[54] | United States | 2- site RCT | To examine the effect of a behavioural lifestyle intervention with partial meal replacement on lowering GWG in Hispanic and non-Hispanic women with overweight or obesity compared to enhanced usual care. | N= 264  I= 132  C= 132 | I= 30.7 ± 5.3  C= 29.7 ± 5.5 | **Baseline BMI**  I= 32.3 ± 5.2  C= 32.6 ± 5.3  **Baseline BMI N (%)**  **Overweight:**  I= 54 (41.9%)  C= 48 (37.5%)  **Obese:**  I= 75 (58.1%)  C= 80 (62.5%) | Primary outcome: GWG.  Secondary outcomes: GDM, pre-eclampsia, c-section, PIH, birthweight, macrosomia, preterm delivery, cardiovascular disease risk factors, diet and physical activity. |
| Phelan *et al.* (2023)[55] | United States | 2- site RCT | To identify the impacts of a pre-pregnancy weight loss intervention on GDM recurrence in women with overweight/obesity and history of previous GDM. | N= 199  I= 105  C= 94 | I= 32.4 ± 4.2  C= 32.9 ± 5.1 | **Baseline BMI**  I= 33.4 ± 6.7  C= 32.1 ± 5.8  **Baseline BMI N (%)**  **Overweight:**  I= 13 (34.2%)  C= 12 (48.0%)  **Obese:**  I= 25 (65.8%)  C= 13 (52.0%) | Primary outcome: GDM.  Secondary outcomes: GWG, fasting glucose, blood pressure, HbA1c, and 6-week infant weight and length, diet and physical activity. |
| Phillips *et al.* (2019)[56] | United States | RCT | To determine the impact of financial incentives and behavioural weight management on adherence to GWG guidelines in pregnant women who are overweight or obese. | N= 136  I= 65  C= 71 | I= 30.9 ± 3.9  C= 30.1 ± 5.0 | **Baseline BMI**  I= 32.2 ± 5.6  C= 32.4 ± 5.9  **Baseline BMI N (%)**  **Obese:**  I= 36 (61%)  C= 39 (60%) | Primary outcome: GWG.  Secondary outcomes: GDM, hypertensive disorders, mode of delivery, birthweight, NICU admission. |
| Poston *et al.* (2015)[57] | United Kingdom | Multicentre-RCT | To test whether a complex intervention based on diet and physical activity could reduce the incidence of GDM and LGA infants in pregnant women with obesity. | N= 1555  I= 783  C= 772 | I= 30.5 ± 5.5  C= 30.4 ± 5.6 | **Baseline BMI**  I= 36.3 ± 5.0  C= 36.3 ± 4.6 | Primary outcomes: GDM, LGA.  Secondary outcomes: pre-eclampsia, GWG, diet and physical activity, maternal anthropometric/biochemical outcomes, mode of delivery, induction of labour, blood loss at delivery, inpatient nights, referral to diabetic antenatal service after OGTT, maternal need for insulin or metformin therapy, birthweight, neonatal death, days in special care baby unit, total inpatient days, discharge on oxygen, infection, retinopathy of preterm delivery, neonatal hypoglycaemia, intraventricular haemorrhage, need for mechanical ventilation and duration, necrotizing enterocolitis, pulmonary haemorrhage, newborn anthropometrics. |
| Quinlivan, Lam and Fisher. (2011)[58] | Australia | RCT | To assess the effect of a four-step multidisciplinary approach of antenatal care in reducing GDM in women who are overweight or obese. | N= 132  I= 67  C= 65 | I= 28.3 ± 0.63  C= 29.5 ± 0.71 | **Baseline BMI N (%)**  **Overweight:**  I= 26 (42%)  C= 30 (49%)  **Obesity class I**:  I= 17 (27%)  C= 15 (25%)  **Obesity class II:**  I= 11 (18%)  C= 9 (15%)  **Obesity class III:**  I= 8 (13%)  C= 7 (11%) | Primary outcome: GDM.  Secondary outcomes: GWG, birthweight. |
| Rakhshani *et al*. (2012)[59] | India | RCT | To examine the impact of yoga interventions on improving pregnancy outcomes in high-risk pregnant women. | N= 93  I= 46  C= 47 | I= 27.8 ± 5.15  C= 27.2 ± 5.2 | **Baseline BMI**  I= 25.1 ± 4.7  C= 25.3 ± 4.9 | Primary outcome: Feasibility.  Secondary outcomes: GDM, hypertensive disorders, preterm delivery, Apgar score, LGA, SGA, birthweight. |
| Renault *et al*. (2014)[60] | Denmark | RCT | To evaluate the impact of a physical activity intervention with or without dietary intervention on GWG in pregnant women with obesity. | N= 425  Physical activity I= 142  Diet + physical activity I= 142  C= 141 | Physical activity I= 30.9 ± 4.9  Diet + physical activity I= 31.2 ± 4.4  C= 31.3 ± 4.2 | **Pre- pregnancy BMI**  Physical activity I= 34.1 ± 4.4  Diet + physical activity I= 34.4 ± 4.2  C= 33.7 ± 3.5 | Primary outcome: GWG.  Secondary outcomes: GDM, hypertensive disorders, c-section, preterm delivery, LGA, SGA, birthweight. |
| Roeder *et al.* (2019)[61] | United States | RCT | To determine if early treatment of maternal hyperglycaemia improves maternal and neonatal outcomes. | N= 202  I= 99  C= 103 | I= 32.7 ± 5.1  C= 33.0 ± 4.1 | **Baseline BMI**  I= 28.1 ± 8.7  C= 27.1 ± 6.3  **Baseline BMI N (%)**  **Underweight:**  I= 2 (2.4%)  C= 1 (1.3%)  **Recommended:**  I= 28 (34.1%)  C= 32 (42.7%)  **Overweight:**  I= 30 (36.6%)  C= 19 (25.3%)  **Obese:**  I= 22 (26.9%)  C= 23 (30.7%) | Primary outcome: fetal hyperinsulinemia.  Secondary outcomes: GDM, neonatal fat mass, weight-for-length percentile at birth, GWG. |
| Sadiya *et al*. (2022)[62] | United Arab Emirates | RCT | To determine the effect of moderate-intensity lifestyle interventions (diet, physical activity and behaviour modification) on GDM incidence in high-risk pregnant women in the UAE. | N= 63  I= 30  C= 33 | I= 32.8 ± 4.1  C= 30.8 ± 5.2 | **Baseline BMI**  I= 28.2 ± 3.9  C= 27.6 ± 6.1 | Primary outcome: GDM.  Secondary outcomes: GWG, birthweight, mode of delivery. |
| Sales *et al*. (2018)[63] | Brazil | RCT | To evaluate the effectiveness of metformin on GDM incidence in pregnant women with obesity. | N= 164  I= 82  C= 82 | I= 28.8 ± 6.0  C= 29.7 ± 6.3 | **Baseline BMI**  I= 37.5 ± 4.7  C= 37.5 ± 5.0 | Primary outcomes: GDM, BMI. |
| Santamaria *et al*. (2016)[64] | Italy | RCT | To evaluate if MI supplementation might decrease GDM rate in pregnant women who are overweight. | N= 220  I= 110  C= 110 | I= 32.1 ± 4.8  C= 32.7 ± 5.3 | **Pre- pregnancy BMI**  I= 26.9 ± 1.3  C= 27.1 ± 1.3 | Primary outcome: GDM.  Secondary outcomes: GWG, PIH, c-section, preterm delivery, birthweight, macrosomia, shoulder dystocia, neonatal hypoglycaemia, insulin treatment, NICU admission. |
| Sartorelli *et al*. (2022)[65] | Brazil | RCT | To assess the effectiveness of a nutritional counselling intervention based on encouraging physical activities and consuming unprocessed/minimally processed foods to prevent excessive GWG in pregnant women who are overweight. | N= 350  I= 174  C= 176 | I= 27 (23–31)  C= 27 (22–32) | **Baseline BMI**  I= 27.2 (26.2–28.3)  C= 26.9 (25.9–28.4) | Primary outcome: GWG.  Secondary outcomes: GDM, pre-eclampsia, mode of delivery, preterm delivery. |
| Seneviratne *et al.* (2016)[66] | New Zealand | RCT | To evaluate the effect of antenatal exercise in pregnant women (overweight/obese) on maternal and perinatal outcomes. | N= 75  I= 38  C= 37 | I= 31.6 ± 4.6  C= 31.1 ± 5.2 | **Baseline BMI**  I= 32.1 ± 4.4  C= 34.1 ± 5.9 | Primary outcome: birthweight.  Secondary outcomes: GDM, PIH, pre-eclampsia, NICU admission, postpartum haemorrhage, c-section, preterm delivery, physical activity, LGA, SGA, neonatal hypoglycaemia, Apgar score, neonatal death, respiratory distress, birth asphyxia, NICU admission. |
| Shahgheibi, Farhadifar and Pouya. (2016)[67] | Iran | RCT | To examine the impact of vitamin D in early pregnancy on GDM rate in women who are at high risk of GDM. | N= 100  I= 50  C= 50 | I= 31.28 ± 6.38  C= 29.00 ± 6.24 | **Baseline BMI**  I= 28.7 ± 4.63  C= 28.7 ± 5.46 | Primary outcomes: GDM, mode of delivery. |
| Shahriari *et al*. (2021)[68] | Iran | RCT | To examine the role of antenatal probiotic supplementation on the risk of GDM and maternal and neonatal outcomes. | N= 542  I= 271  C= 271 | I= 31.83 ± 5.80  C= 32.20 ± 5.51 | **Baseline BMI**  I= 30.27 ± 6.10  C= 30.21 ± 5.77  **Baseline BMI N (%)**  **Normal:**  I= 50 (20.7%)  C= 45 (16.9%)  **Overweight:**  I= 48 (19.9%)  C= 69 (25.9%)  **Obese:**  I= 143 (59.3%)  C= 152 (57.1%) | Primary outcome: GDM.  Secondary outcomes: pre-eclampsia, mode of delivery, polyhydramnios, birthweight, macrosomia. |
| Simmons *et al*. (2017)[69] | Across nine European countries (United Kingdom, Republic of Ireland, Netherlands, Austria, Poland, Italy (Padua and Pisa), Spain, Denmark (Odense and Copenhagen) and Belgium) | Multicentre-RCT | To compare the effectiveness of three different lifestyle interventions in decreasing GDM risk. | N= 436  Diet I= 113  Physical activity I= 110  Diet + physical activity I= 108  Usual care C= 105 | Diet I= 32.5 ± 5.5  Physical activity I= 31.7 ± 5.1  Diet + physical activity I= 31.9 ± 5.3  Usual care C= 31.8 ± 5.6 | **Pre- pregnancy BMI**  Diet I= 33.7 ± 4.0  Physical activity I= 33.9 ± 4.4  Diet + physical activity I= 33.8 ± 3.9  Usual care C= 33.4 ± 3.5  **Baseline BMI**  Diet I= 34.7 ± 4.2  Physical activity I= 34.4 ± 3.8  Diet + physical activity I= 34.5 ± 4.0  Usual care C= 34.2 ± 3.9 | Primary outcomes: GWG, FPG, insulin resistance.  Secondary outcomes: GDM, birthweight, SGA, LGA, diet and physical activity. |
| Syngelaki *et al.* (2016)[70] | United Kingdom | RCT | To test the hypothesis that metformin is associated with a lower median neonatal birth-weight z score in pregnant women (without DM) with a BMI over 35 kg/m^2^. | N= 450  I= 225  C= 225 | I= 32.9 (27.3–36.2)  C= 30.8 (26.6–34.4) | **Baseline BMI**  I= 38.6 (36.5–41.5)  C= 38.4 (36.3–41.9) | Primary outcome: birthweight.  Secondary outcomes: GDM, GWG, pre-eclampsia, growth restriction, stillbirth, preterm delivery, neonatal hypoglycaemia, NICU admission, Apgar score. |
| Thornton *et al*. (2009)[71] | United States | Multicentre-RCT | To compare the effect of active nutritional/ behavioural intervention versus conventional prenatal management on perinatal outcomes of pregnant women with obesity. | N= 257  I= 124  C= 133 | I= 26.8 (median)  C= 27.3 (median) | **Baseline BMI**  I= 37.41 ± 7.01  C= 38.22 ± 7.48 | Primary outcomes: GDM, pre-eclampsia, ketonuria, PIH, preterm delivery, macrosomia, Apgar score, c-section, postpartum haemorrhage/infection. |
| Valdés *et al*. (2018)[72] | Chile | Multicentre-RCT | To determine the effect of metformin in preventing GDM in women with pregestational insulin resistance. | N= 141  I= 68  C= 73 | I= 31 (26.5–35.5)  C= 31 (27–34) | **Pre- pregnancy BMI**  I= 31.6 (28.9–34.2)  C= 31.1 (27.4–34.8) | Primary outcomes: GDM, pre-eclampsia, birthweight, LGA, SGA, preterm delivery, c-section. |
| Van Horn *et al.* (2018)[73] | United States | RCT | To determine if a technology-enhanced antenatal intervention could improve excess GWG and benefit the mother and child. | N= 281  I= 140  C= 141 | I= 33 ± 4  C= 34 ± 4 | **Pre- pregnancy BMI**  I= 31 ± 4  C= 31 ± 4 | Primary outcome: GWG.  Secondary outcomes: GDM, maternal blood pressure, birthweight, LGA, SGA, metabolics, newborn anthropometrics, diet quality, physical activity, c-section. |
| Vanky *et al*. (2004)[74] | Norway | RCT | To test if metformin can prevent pregnancy complications in women with PCOS. | N= 40  I= 18  C= 22 | I= 28.9 ± 4.8  C= 28.3 ± 3.7 | **Baseline BMI**  I= 32.1 ± 6.1  C= 29.3 ± 8.0 | Primary outcome: Androgen levels.  Secondary outcomes: GDM, pre-eclampsia, preterm delivery, birthweight, Apgar score. |
| Vanky *et al*. (2010)[75] | Norway | Multicentre-RCT | To test if taking metformin from the 1^st^ trimester to delivery could prevent pregnancy complications in women with PCOS. | N= 274  I= 136  C= 138 | I= 29.6 ± 4.4  C= 29.2 ± 4.4 | **Baseline BMI**  I= 29.5 ± 7.0  C= 28.5 ± 7.2 | Primary outcomes: GDM, pre-eclampsia, preterm delivery, composite pregnancy complications.  Secondary outcomes: GWG, mode of delivery, birthweight, Apgar score. |
| Vesco *et al*. (2014)[76] | United States | RCT | To assess whether a group-based weight management intervention for pregnant women with obesity is effective in limiting GWG. | N= 118  I= 58  C= 60 | I= 32.4 ± 5.1  C= 31.2 ± 4.6 | **Baseline BMI**  I= 36.7 ± 5.2  C= 36.8 ± 4.7  **BMI N (%)**  **30.0- 34.9:**  I= 25 (45%)  C= 25 (43%)  **35.0-39.9:**  I= 19 (34%)  C= 21 (36%)  **≥ 40.0:**  I= 12 (21%)  C= 12 (21%) | Primary outcome: GWG.  Secondary outcomes: GDM, hypertensive disorders, mode of delivery, LGA, SGA, macrosomia, preterm delivery, neonatal hypoglycaemia, hyperbilirubinemia, NICU admission, perinatal mortality. |
| Vinter *et al*. (2011)[77] | Denmark | RCT | To test the efficacy of an antenatal lifestyle intervention on GWG and obstetric/neonatal outcomes among women with obesity. | N= 360  I= 180  C= 180 | I= 29 (27–32)  C= 29 (26–31) | **Baseline BMI**  I= 33.4 (31.7–36.5)  C= 33.3 (31.7–36.9)  **BMI N (%)**  **30.0- 34.9:**  I= 95 (63.3%)  C= 102 (66.2%)  **35.0-39.9:**  I= 42 (28%)  C= 45 (29.2%)  **40.0-45.0:**  I= 13 (8.7%)  C= 7 (4.6%) | Primary outcomes: GDM, GWG, pre-eclampsia, PIH, c-section, macrosomia, LGA, NICU admission. |
| Vitale *et al*. (2021)[78] | Italy | RCT | To assess the impact of MI supplementation on GDM rates and body water distribution in women who are overweight. | N= 250  I= 125  C= 125 | I= 27.18 ± 6.03  C= 27.95 ± 4.90 | **Pre- pregnancy BMI**  I= 27.00 ± 1.49  C= 26.68 ± 1.56 | Primary outcomes: GDM and body water distribution.  Secondary outcomes: PIH, preeclampsia, macrosomia, c-section, preterm delivery, lipid metabolism, shoulder dystocia, neonatal hypoglycaemia, NICU admission. |
| Walsh *et al*. (2012)[79] | Ireland | RCT | To undertake a randomised controlled trial of a low-GI diet in women who previously delivered an infant weighing greater than 4000g. | N= 800  I= 394  C= 406 | I= 32.0 ± 4.2  C= 32.0 ± 4.2 | **Baseline BMI**  I= 26.8 ± 5.1  C= 26.8 ± 4.8 | Primary outcome: birthweight.  Secondary outcomes: GDM, GWG. |
| Wang *et al*. (2017)[80] | China | RCT | To examine the efficacy of regular exercise in early gestation to prevent GDM in Chinese women who are overweight/obese. | N= 300  I= 150  C= 150 | I= 32.14 ± 4.57  C= 32.50 ± 4.91 | **Pre-pregnancy BMI**  I= 26.75 ± 2.74  C= 26.82 ± 2.76  **Baseline BMI N (%)**  **≥ 28.0:**  I= 39 (26.0%)  C= 38 (25.3%) | Primary outcome: GDM.  Secondary outcomes: GWG, PIH, pre-eclampsia, mode of delivery, physical activity, biochemical outcomes, preterm delivery, Apgar score, birthweight, SGA, LGA, macrosomia. |
| Wang *et al*. (2015)[81] | China | Pilot cluster-RCT | To investigate the effectiveness of early lifestyle counselling in preventing GDM in high-risk population. | N= 272  I= 134  C= 138 | I= 31.0 ± 3.8  C= 30.3 ± 3.6 | **Pre-pregnancy BMI**  I= 22.9 ± 3.6  C= 23.1 ± 3.6 | Primary outcome: GDM.  Secondary outcome: GWG. |
| Wolff *et al*. (2008)[82] | Denmark | RCT | To test if a 10-hr dietary intervention can limit GWG and alter glucose metabolism in pregnant women with obesity. | N= 50  I= 23  C= 27 | I= 28 ± 4  C= 30 ± 5 | **Baseline BMI**  I= 34.9 ± 4  C= 34.6 ± 3 | Primary outcomes: GWG, glucose metabolism.  Secondary outcomes: GDM, PIH, pre-eclampsia, birthweight, c-section, newborn anthropometrics, diet. |
| Zhang *et al*. (2019)[83] | China | RCT | To test the efficacy of individualised low-GI diet sessions based on a diet GL calculation tool on maternal and fetal insulin resistance and eating behaviours in pregnant women who are overweight/obese. | N= 400  I= 200  C= 200 | I= 28.1 ± 3.6  C= 28.0 ± 3.7 | **Baseline BMI**  I= 28.4 ± 3.0  C= 28.0 ± 3.0 | Primary outcomes: GDM, maternal insulin and C-peptide levels.  Secondary outcomes: GWG, PIH, c-section birthweight, preterm delivery, macrosomia. |
| Zhang *et al*. (2022)[84] | China | RCT | To investigate the impact of soluble fibre supplements on GDM prevention in women who are overweight/obese prior to pregnancy. | N= 104  I= 52  C= 52 | I= 31.13 ± 4.21  C= 29.96 ± 4.07 | **Baseline BMI**  I= 26.05 (24.89–28.05)  C= 25.90 (24.54–28.35) | Primary outcome: GDM.  Secondary outcomes: GWG, PIH, pre-eclampsia, PROM, polyhydramnios, c-section, glucose/lipid parameters, diet, birthweight, preterm delivery, macrosomia, SGA. |

BMI= body mass index; C= control; c-section= caesarean section; DCI= D-chiro-inositol; DHA= docosahexaenoic acid; DPP= Diabetes prevention program; EPA= eicosapentaenoic acid; FPG= fasting plasma glucose; GDM= gestational diabetes mellitus; GI= glycaemic index; GL= glycaemic load; GWG= gestational weight gain; HDU= high dependency unit; IUGR= intrauterine growth restriction; I= intervention; ICU= intensive care unit; IOM= institute of medicine; LGA= large-for-gestational-age; N= number; MI= myo-inositol; MI/DCI= myo-inositol and D-chiro-inositol; NICU= neonatal intensive care unit; OGTT= oral glucose tolerance test; PCOS= polycystic ovary syndrome; PIH= pregnancy induced hypertension; PROM= premature rupture of membranes; RCT= randomised controlled trial; SGA= small-for-gestational age.

**Table A5.** Summary of criteria used for GDM risk stratification and intervention characteristic

| Reference | Risk stratification | Intervention characteristics | Gestational age of intervention delivery |
| --- | --- | --- | --- |
| Ajmani and Sircar (2020)[1] | At least one risk factor: BMI ≥25 kg/m^2^, family history of DM, previous history of GDM or macrosomic infant and glycosuria in morning urine sample. | I= vitamin D (60000 IU per week) in the form of cholecalciferol sachet.  C= no intervention.  *Women in both groups received iron, calcium and vitamins B and C supplements. | Started from 13–24 until 26 weeks’ gestation. |
| Amaefule *et al.* (2022)[2] | At least one risk factor as per NICE criteria: BMI ≥30 kg/m^2^, family history of DM in a first-degree relative, high-risk ethnicity, PCOS, previous history of GDM or macrosomic infant ≥ 4.5 kg. | I= MI plus folic acid (2 g MI and 200 μg of folic acid, twice daily).  C= placebo (identical looking and tasting powder of Xylitol filler with 200 μg/day folic acid). | Started at 12^+0^–15^+6^ weeks’ gestation until delivery. |
| Al Wattar *et al.* (2019)[3] | At least one metabolic risk factors; obesity (BMI ≥ 30 kg/m^2^), elevated serum triglycerides (≥ 1.7 mmol/L) or chronic hypertension (≥140 mmHg systolic BP or ≥90 mmHg diastolic BP). | I= individualised Mediterranean diet (one-on-one session and 2 further group counselling sessions) delivered by the trial dietitian and trained researchers.  Women were asked to set and record individualised goals following the "SMART" goal model to implement dietary changes. The dietary intervention focused on plant-based food, moderate to high consumption of fish and poultry, low to moderate consumption of dairy products and limited intake of red meat, processed meat, sweetened beverages, fast food and foods that contain animal fat. Diet was supplemented with extra-virgin olive oil (0.5 L/week) and mixed nuts (walnuts, hazelnuts and almonds (30 g/day)).  C= routine antenatal care which included dietary advice as per UK national recommendations for antenatal care and weight management in pregnancy. | At 18, 20 and 28 weeks’ gestation. |
| Basu *et al*. (2021)[4] | Women with obesity (BMI ≥30 kg/m^2^) with other risk factors (previous history of GDM and/or family history of DM). | I= 2 cups (280 g) of frozen blueberries and 12 g soluble fiber daily, provided to the women biweekly, along with short sessions with the dietitian and nurse practitioner.  Women were instructed to avoid consuming fruit juice, to consume the blueberries as a snack by itself not in combination with any other food items and to add the fiber to their meals in soups, gravies and shakes.  C= standard prenatal care (biweekly sessions by the nurse and dietitian).  Both groups maintained 24-hr food recalls and received handouts on nutrition education based on the USDA Dietary Guidelines for Americans for pregnant women and recommendations were based on a balance of carbohydrates, fat and protein. | Started at recruitment <20 weeks of gestation and continued intervention for 18 weeks. |
| Begum *et al*. (2009)[5] | PCOS | *All women received metformin from preconception for 6 weeks according to their BMI: 1500 mg/ day for BMI ≤29.0, 2000 mg/ day for BMI 30.0–32.0 and 2500 mg/ day for BMI >32.0.  Those who conceived either:  I= continued metformin until delivery **OR**  C= discontinued at 8 weeks of pregnancy. | Started at early pregnancy and continued until delivery. |
| Bisson *et al*. (2015)[6] | Pre-pregnancy BMI ≥30 kg/m^2^ | I= a supervised exercise program with free membership in a hospital-based conditioning centre (36 sessions over 12 weeks).  Based on the ACSM Guidelines, exercise consisted of 3 weekly 1-hr sessions (individually supervised once a week). Each session included: 5–10 min warm-up, 15–30 min treadmill walks, 20 min muscular work-out and a cool-down period. Duration of the cardiovascular training increased gradually from 15 min during the first week to 30 min by the end of the first month. Exercise intensity was self-monitored with heart rate monitors. On non-training days, participants were advised to stay as active as possible.  C= usual activities without being restrained from doing physical activity.  *Both groups were provided with a pamphlet (from Kino-Québec, an agency promoting physical activity) about the benefits of physical activity and appropriate exercises throughout pregnancy. | Started at 15 weeks’ of gestation. |
| Brink *et al*. (2018)[7] | Women with obesity (BMI ≥30 kg/m^2^) with other risk factors (family history of DM in a first-degree relative, high-risk ethnicity, PCOS, previous history of GDM, unexplained intrauterine fetal death or macrosomic infant ≥ 4.5 kg). | I= metformin initiated at 500 mg/day for the first week and increased to 1000 mg/day from the start of the second week up to delivery.  C= no intervention.  *Women in both groups followed a standard diet (2000 calories/day). | Started at 14 weeks’ gestation until delivery. |
| Bogaerts *et al*. (2013)[8] | Pre-pregnancy BMI ≥29 kg/m^2^ | Brochure group I= a specifically designed brochure provided nutritional advice and physical activity during pregnancy with tips to limit excessive GWG.  Lifestyle group I= the same brochure and an additional motivational lifestyle intervention (4 group sessions, maximum 3 women per session) led by a trained midwife. The sessions aimed to promote balance between energy intake and energy expenditure based on the active and healthy food pyramid for pregnant women.  Diet composition was based on the official National Dietary Recommendations: 50–55% carbohydrates, 30–35% fat and 9–11% protein.  The intervention focused on providing theoretical insights applied to the woman’s own lifestyle and eating habits using their 7-day food diary; training on label-reading and shopping methods were also implemented. Strategies to improve physical activity levels were reviewed. The lifestyle intervention program was based on the stages of the behavioural change model of Prochaska and co-workers, as well as on the concept of motivational interviewing.  C= routine antenatal care. | At <15, 18–22, 24–28, 30–34 weeks’ gestation. |
| Bruno *et al.* (2017)[9] | Pre-pregnancy BMI ≥25 kg/m^2^ | I= specific personalized dietary antenatal lifestyle intervention focusing on low-glycaemic, low-saturated fat diet, delivered by a dietitian.  The dietary plan provided three main meals and three snacks rich in plant foods, cereals, legumes and fish, with olive oil as the main source of fat and moderate to no intake of red wine. Women were advised to avoid food high in GI and saturated fat and increase the intake of vegetables and fruit with a low-GI. No specific recommendation on food quantities, caloric intake, meal composition or meal distribution was provided.  Diet composition: 1500 kcal/day (additional 200 kcal/day for obese and 300 kcal/day for overweight), 55% carbohydrates (80% complex carbohydrates with a low-GI and 20% simple carbohydrates), 20% protein (50% animal and 50% vegetable) and 25%fat (12% mono-unsaturated, 7% polyunsaturated and 6% saturated).  C= Received standard recommendations and a simple nutritional booklet developed according to the Italian Guidelines for a healthy diet and physical activity during pregnancy. Women were asked to attend an initial 1-hr counselling session with a dietitian, who provided general recommendations on diet and physical activity.  *Women in both groups received the same prescription of physical activity. The physical activity component of the intervention focused on developing a more active lifestyle. Recommendations were based on the ACOG and the ACSM for pregnant women. Women were advised to participate in 30 min of moderate-intensity exercise at least three times a week. | Started at 9-12 weeks’ gestation with follow-up planned at 16, 20, 28 and 36 weeks’ gestation. |
| Callaway *et al*. (2010)[10] | Baseline BMI ≥30 kg/m^2^ | I= an individualised exercise program with regular exercise advice and paper-based diaries for self-monitoring (an energy expenditure goal of 900 kcal/week).  For the development of individualised plans, assessment of readiness for change and goal setting, an initial face-to-face interview was offered with a qualified physiotherapist. Women were monitored every 4 weeks by physiotherapists, with phone calls between visits by a research midwife to assess the adherence to the program. Additional face-to-face support was offered to women who were not meeting exercise goals to identify barriers and modify the exercise plan.  C= routine obstetric care. | At 12, 20, 28 and 36 weeks’ gestation. |
| Callaway *et al*. (2019)[11] | Baseline BMI ≥25 kg/m^2^ | I= mixture of probiotics (Lactobacillus rhamnosus and Bifidobacterium animalis subspecies lactis (BB-12)) at a dose of 1 X 10^9^ CFU/per day.  C= matched placebo (microcrystalline cellulose and dextrose anhydrate capsules). | Started at enrolment (<20 weeks’ gestation) until delivery. |
| Celentano *et al*. (2018)[12] | Elevated fasting glucose in the 1^st^ or early 2^nd^ trimester (glycaemia ≥ 5.1 mmol/L or 92 mg/dL and <7.0 mmol/L or 126 mg/dL). | I= the study had three arms in which women received either MI (4000 mg MI plus 400 μg/day folic acid, divided into 2 sachets daily, taken with at least a 6 hr interval), DCI (500 mg DCI plus 400 μg/day folic acid in a single cap), or a combination of MI and DCI from early pregnancy until delivery (1100 mg MI plus 27.6 mg DCI, divided into 2 capsules daily, taken with at least a 6 hr interval).  C= placebo (400 μg/day folic acid).  *All participants received dietary advice according to the ADA recommendations and counselling regarding safe physical activities. | NA. |
| Chan *et al*. (2018)[13] | At least one risk factor: age ≥ 35 years at the estimated date of delivery, pre-pregnant BMI or BMI at the 1^st^ trimester ≥ 25 kg/, family history of DM in a first-degree relative, previous history of GDM or macrosomic infant ≥ 4 kg. | I= dietitian-led lifestyle modification program, including an initial 1-hr, face-to-face session and 20 min follow-up sessions (face-to-face or phone calls, bi-weekly in the first 2 months, then monthly).  Participants received an individualised menu plan and booklets focusing on fruit and vegetable consumption, low-fat and low-GI diet with a moderate intake of carbohydrates. Women were encouraged to have an exercise consultation at least once and advised to do 30 min of light to moderate-intensity low impact aerobic exercise at least three times a week.  C= routine antenatal care plus educational booklet on diet and exercise during pregnancy. | Started at the 1^st^ antenatal booking (≤ 12 weeks’ of gestation) to 24–28 weeks’ gestation. |
| Chiswick *et al.* (2015)[14] | Baseline BMI ≥30 kg/m^2^ | I= metformin initiated at one tablet (500 mg) daily for the first week and increased by one tablet daily each week over 5 weeks, to achieve either the maximum tolerable dose or the maximum permitted dose of 2500 mg taken as two to three divided doses. Participants were reviewed face-to-face or by telephone during pregnancy; around the time of delivery and 3 months postnatally.  C= matched placebo tablets. | Started at 12–16 weeks’ gestation until delivery. |
| Corcoy *et al*. (2020)[15] | Pre-pregnancy BMI ≥29 kg/m^2^ | I= vitamin D3 (1600 IU divided into 4 tablets daily) with or without an additional lifestyle intervention on top of usual multivitamins supplements.  C= matched placebo with or without an additional lifestyle intervention on top of usual multivitamins supplements.  *Lifestyle intervention included healthy eating and physical activity. | Started at ≤ 19^+6^ weeks’ gestation until delivery. |
| D’Anna *et al*. (2013)[16] | Family history (a first-degree relative with T2DM) | I= MI plus folic acid (2 g MI and 200 μg folic acid twice a day).  C= placebo (200 μg folic acid twice a day). | Started at 12–13 weeks’ gestation until delivery. |
| D’Anna *et al.* (2015)[17] | Pre-pregnancy BMI ≥30 kg/m^2^ | I= MI plus folic acid twice a day (2 g MI and 200 μg folic acid).  C= placebo (200 μg folic acid twice a day). | Started at 12–13 weeks’ gestation until delivery. |
| Daly *et al*. (2017)[18] | Baseline BMI ≥30 kg/m^2^ | I= medically supervised exercise sessions by a researcher (3 per week). Women were encouraged to set personal goals using the "SMART" approach. Women received an invitation to a private Facebook group to share healthy lifestyle advice and to improve adherence with the exercise. The program consisted of 50–60 min of exercise with a 10 min warm-up, 15–20 min of resistance or weights, 15–20 min of aerobic exercises and a 10 min cool-down.  C= routine prenatal care. | Started at <17 weeks’ gestation and for up to 6 weeks postpartum. |
| Deng *et al.* (2021)[19] | At least one risk factor: age ≥35 years, pre-pregnancy overweight or obesity (BMI ≥24 kg/m^2^), family history of DM in a first-degree relative, history of PCOS, previous history of GDM or macrosomic infant, elevated fasting glucose in early pregnancy (≥5.1 mmol/L), history of or current abnormal lipid metabolism | I= comprehensive dietary and lifestyle intervention (7 sessions) delivered by researchers.  In the first 1-hr session at the clinic, diet history and exercise habits were determined using simplified Food Frequency Questionnaire prepared by Zhao (2011) and the Danish Physical Activity Scale. Then, researchers developed the diet and exercise program based on the woman’s history.  The diet and exercise program included a one-on-one session (20 min per person) to distribute the educational manuals and to record the diet and exercise diaries (3 days a week). WeChat was used by nurses to remind participants to adhere to the program and was also offered to women who had limited availability. In addition, sample meals and food model samples were presented to the participants.  Researchers adjusted the contents of the intervention based on the diary and biochemical markers and addressed any challenges related to the program.  The diet and exercise program were developed according to the recommendations of the Chinese Nutrition Society.  Diet composition: Energy needs for each woman were individually calculated by nutritionists according to weight and level of activity, 50%–60% carbohydrates, 20–30 % fats and 15–20% protein. Researchers encouraged women to choose high-fiber bread, whole meal products or multigrain rice as a staple food, consume more vegetables and less high-sugar fruits, choose fat-free or low-fat versions of milk and milk products and use moderate amounts of oil.  The exercise program was developed according to the recommendations of the ACOG and designed by exercise experts. Women were recommended to perform 20 –30 min of moderate-intensity exercise (walking, yoga and gestational gymnastics) at least 5 days a week.  C= routine health management provided at the local antenatal clinic. | Started at 14 weeks’ gestation, until the OGTT visit at 24–28 weeks’ gestation. |
| Ding *et al*. (2021)[20] | Baseline BMI ≥24 kg/m^2^ | I= personalized dietary and exercise intervention (3 face-to-face sessions), with the help of WeChat as a monitoring tool to promote adherence to the treatment plan.  Women received detailed pregnancy diet and exercise guidance information and had the opportunity to ask questions to the dietitians. Dietitians provide reference to dietary guidelines for pregnant women (once a week).  The approach was based on ideal body weight and following “the guide of diagnosis and treatment of GDM (2014)”. For BMI of 24.0–27.9, early pregnancy estimated energy requirement was 25–30 kcal/kg/day with an additional 200 kcal for mid-pregnancy. For BMI ≥ 28.0, early pregnancy estimated energy requirement was 20–25 kcal/kg/ day with an additional 200 kcal for mid-pregnancy. Minimum energy intake was not less than 1500 kcal at early pregnancy and 1800 kcal at mid-pregnancy. A general healthy diet was recommended of 50–60% carbohydrates, <30% fat, 1.0-1.3 g/kg/day protein.  Women were advised to complete a daily walking exercise plan of at least 6,000 steps per day.  C= routine nursing group were given a general advice session about pregnancy nutrition and weight management. | Started at 8–12 weeks of gestation. |
| Dodd *et al*. (2019)[21] | Baseline BMI ≥25 kg/m^2^ | I= metformin initiated at one tablet (500 mg/day) for the first week and increased to two tablets twice per day each week over 4 weeks as tolerated, to achieve the maximum dose of 2000 mg per day. Participants were evaluated face-to-face or by telephone during pregnancy; around the time of delivery and 3 months postnatally.  C= identical placebo. | Started 10–20 weeks’ gestation until delivery. |
| Dodd *et al*., (2014)[22] | Baseline BMI ≥25 kg/m^2^ | I= comprehensive dietary and lifestyle intervention including dietary, exercise and behavioural strategies (6 sessions; 3 face-to-face and 3 by telephone) delivered by a research dietitian and trained research assistants.  The dietary advice was in line with Australian standards to maintain a balanced diet and to limit the consumption of foods high in refined carbohydrates and saturated fats, with increase in the daily intake of fibre, fruit (2 servings), vegetables (5 servings) and dairy products (3 servings). The program included individualised information such as meal plans, easy healthy recipes along with guidelines for food preparation, simple food substitutions (e.g., limit sugary drinks, added sugar and foods high in refined carbohydrates and low-fat alternatives) and healthy snacking choices. Women were advised to set achievable goals for lifestyle changes and were asked to self-monitor their progress using a logbook. Women were also encouraged to identify any potential barriers to be assisted in problem solving and achieve the goals. Tailored implementation interventions were guided by stage theories of health decision making.  The physical activity program focused on advising women to increase the amount of walking and incidental activity.  C= standard care according to state-wide perinatal practice and local hospital guidelines. | At 10–20, 22.24, 28,32 and 36 weeks’ gestation. |
| Eslami *et al*. (2018)[23] | Baseline BMI ≥25 kg/m^2^ | I= lifestyle training package consists of 60- to 90 min group lecture session followed by 30 min question and answer session (2 sessions) delivered by trained interventionist in prenatal health.  The women were provided with an educational booklet developed based on the recommendations of WHO and the Iranian Ministry of Health. Participants had the opportunity to ask questions and received educational text messages throughout the trial.  C= routine antenatal care. | Started at 16–20 weeks’ gestation. |
| Esmaeilzadeh *et al*. (2022)[24] | Pre-pregnancy BMI ≥25 and <30 kg/m^2^ | I= MI plus folic acid (2000 mg MI and 400 μg folic acid twice a day).  C= placebo (400 μg/day folic acid). | Started at 12–14 weeks’ gestation until 24 weeks’ gestation. |
| Farren *et al*. (2017)[25] | Family history (a first-degree relative with type 1 or 2 DM) | I= a combination of MI, DCI and folic acid (1100 mg MI, 27.6 mg DCI and 400 μg folic acid).  C= placebo (400 μg/day folic acid). | Started at 10–16 weeks’ gestation (1^st^ antenatal visit). |
| Ferrara *et al*. (2020)[26] | Pre-pregnancy BMI between 25-40 kg/m^2^ | I= a telehealth lifestyle weekly individual intervention (13 sessions, 2 face-to-face and 11 by telephone) adapted from the DPP delivered by a dietitian.  Based on social cognitive theory and the transtheoretical model, the dietitian used motivational interviewing techniques and a step-wise, phased method to encourage changes. The lifestyle advice focused on a balanced diet, high in vegetables, fruits, lean protein and fiber foods with low saturated and trans fats and added sugars. Physical activity recommendations focused on being active and performing safe activities such as brisk walking (150 min per week of moderate-intensity to vigorous-intensity physical activity).  The intervention focused also on stress management and women were advised of their GWG goal (based on IOM recommendations) and were provided with a workbook, a scale for self-weighing and a personalized graph to track their weight. Maintenance sessions by telephone until 38 weeks’ gestation were also offered.  C= standard antenatal care in addition to 4 study newsletters focused on maternal health and safety without addressing GWG. | Started at 8–15 weeks’ gestation. |
| Gallagher *et al*. (2018)[27] | Baseline BMI ≥25 kg/m^2^ | I= dietary and lifestyle intervention with behavioural and social support strategies (20 sessions) delivered by an experienced nutritionist.  The intervention was developed by the Diabetes Prevention Program and Look AHEAD study and followed the 2009 IOM guidelines for GWG. Women received a 60 min introductory session followed by individual sessions. At the 1^st^ session, women were provided with individualised meal plans and materials to help estimating portion sizes and caloric counting. Weight was monitored along with food/exercise logs. The program consisted of 20 modules covered face-to-face or by email/post and reinforced through telephone/email contact. Group sessions were offered once every eight weeks during pregnancy. Women were offered one physical activity session taught by a certified prenatal exercise specialist.  C= usual care including a single introductory session (20 -30 min) about basic nutrition for pregnancy based on MyPlate and American Academy of Nutrition guidelines. Additionally, once every 8 weeks women were offered group meetings related to health during pregnancy with less focus on calorie counting. | Started at 12–15^+6^ weeks’ gestation until delivery. |
| Guelfi *et al*. (2016)[28] | Previous history of GDM | I= 14-week stationary cycling program (3 sessions/ week) supervised by an exercise physiologist who monitor the duration and intensity of the exercise.  As part of the trial, each woman received an upright cycle ergometer. Each session started with a 5 min warmup then 5 min periods of continuous moderate-intensity cycling alternating with 5 min periods of interval cycling and concluding with 5 min cool down followed by light stretching. The duration of each session started at 20–30 min and gradually increased by 5 min increments every 2–3 weeks to a maximum of 60 min, taking into account each woman’s baseline fitness level and pregnancy.  C= standard care. | Started at 14 weeks’ gestation. |
| Garnæs *et al*. (2016)[29] | Pre-pregnancy BMI ≥28 kg/m^2^ | I= weekly face-to-face exercise program (3 sessions each last for 60 min) supervised by a physical therapist.  The program was developed in line with the ACOG recommendations. Sessions consisted of 35 min endurance training (such as treadmill walking/jogging) and 25 min of resistance training (such as squats, push-ups, diagonal lifts). The endurance intensity was set to ~80% of maximal capacity. The program was adjusted depending on each woman’s strength level. Additionally, participants were advised to complete a 50 min home exercise program at least once weekly (35 min endurance training and 15 min strength exercises) and to perform pelvic floor muscle exercises daily.  To monitor GWG, a curve of recommended weight gain throughout pregnancy in accordance with 2009 IOM guidelines was given to the women. In addition, women were offered a one motivational interview (individually or group) session.  C= standard maternity care by midwife, general practitioner and/or obstetrician based on the Norwegian national guidelines which included information about healthy lifestyle. | Started at baseline 12–18 weeks’ gestation until delivery. |
| Gonzalez-Plaza *et al*. (2022)[30] | Pre-pregnancy BMI of ≥30 kg/m^2^ | I= a complex digital intervention, based on social cognitive theory delivered by a midwife. Women were provided with personalised information (twice a week via SMS or videos using Hangouts App, Google LLC) to promote physical activity and healthy eating and were monitored through the app and motivated or reinforced monthly by the research team.  The intervention included behaviour-changing method of self-control, self-efficacy and improvement of outcome expectations along with addressing barriers to the use of a smartband. The messages included information regarding pregnancy, physiological changes in the mother and fetus, healthy eating habits, weight gain, physical activity, labour and postpartum. Women had the opportunity to ask questions and get an immediate response from the midwife (<1 hr).  Women were recommended to perform 10,000 steps a day (30 min/day of weekly moderate physical activity in ≥5 days) according to the ACOG recommendations. The smartband vibrates during extended periods of inactivity or send awards when goals were met.  C= standard prenatal care based on the Pregnancy Monitoring Protocol in Catalonia included health education about physical activity, GWG and eating behaviours (oral information and written material). At least 30 min/day of weekly moderate physical activity (≥5 days) was recommended according to the ACOG recommendations. A balanced Mediterranean diet of 1800 kcal was prescribed in order to meet the IOM recommendation (GWG between 5–9 kg). | Started at 12–18 weeks’ gestation. |
| Halkjær *et al*. (2020)[31] | Baseline BMI ≥30 and <35 kg/m^2^ | I= probiotic mixture Vivomixx® (4 vegetable capsules per day; a total of 450 billion CFU/day).  Vivomixx® contains the following strains: Streptococcus thermophilus DSM 24,731, bifidobacteria (Bifidobacterium breve DSM 24,732, Bifidobacterium longum DSM 24,736, Bifidobacterium infantis DSM 24,737) and lactobacilli (Lactobacillus acidophilus DSM 24,735, Lactobacillus plantarum DSM 24,730, Lactobacillus paracasei DSM 24,733, Lactobacillus delbrueckii subsp. bulgaricus DSM 24,734).  C= placebo capsules contained microcrystalline cellulose, magnesium stearate and silicon dioxide. | Started at 14–20 weeks’ gestation until delivery. |
| Harrison *et al*. (2013)[32] | Validated risk prediction tool (advanced maternal age, increased BMI, family history of DM, previous history of GDM, history of poor obstetric outcomes and Asian descent). | I= individualised antenatal lifestyle intervention (4 sessions) delivered by a health coach based on Social Cognitive Theory.  Women received pregnancy-specific dietary advice and simple healthy eating and physical activity tips. Women were asked to set simple short-term goals and increase self-efficacy and self-monitoring as part of the behavioural change strategies. Self-monitoring strategies included pedometers and the use of weight gain charts according to the IOM recommendations. Women were provided with the same written resources as controls in addition to information to promote optimal health, GWG and lifestyle.  C= standard antenatal care which involved a single general education session based on the Australian Dietary and Physical Activity Guidelines. Written materials of these guidelines were also provided. | Scheduled around antenatal appointments at 14–16, 20, 24 and 28 weeks’ gestation. |
| Herring *et al*. (2015)[33] | Baseline BMI (1^st^ trimester) between 25-45 kg/m^2^ | I= Technology-based behavioural intervention delivered by a health coach (15–20 min weekly for the first two weeks and twice monthly thereafter) based on Social Cognitive Theory and Social Ecological Model.  The intervention included lifestyle, physical activity and GWG coaching using Facebook, text-messaging (3–4 weekly) and/or a website, booklets and phone-calls. The dietary goals focused on diet quality and quantity. Women were encouraged to limit sugar-sweetened beverages, junk and high-fat foods and choose low calorie beverages and nutrient-rich foods. The exercise goals were developed according to the ACOG (5,000 steps daily). Women were supplied with pedometers and a walking DVD and were asked to weigh themselves and follow the 2009 IOM guidelines for weight gain goals.  C= standard obstetric care with weight gain recommendations (ACOG). | Started at 12^+4^ weeks’ gestation on average until delivery. |
| Jamal, Milani and Al-Yasin. (2012)[34] | PCOS | Metformin I= two tablets twice (2000 mg/day).  Aspirin I= one tablet (80 mg/ day).  C= no intervention.  *All women received one ferrous sulfate, folate and one multivitamin tablet daily. | Started at 6–12 weeks’ gestation until delivery. |
| Kennelly *et al*. (2018)[35] | Baseline BMI between 25-39.9 kg/m^2^ | I= healthy Lifestyle intervention (1 face-to-face session) conducted individually or in pairs.  The intervention focused on promoting healthy lifestyle and safe physical activities during pregnancy. The dietary and exercise goals were individualised and based on the "SMART" goals principle. The recommended diet was eucaloric with healthy carbohydrates portions. Women were advised to change high-GI foods for low-GI options. The exercise advice was based on the ACOG guidance and women were asked to perform 30 min of moderate exercise 5–7 days per week.  The received information was reinforced through delivery channels including: a smartphone application, emails every 2 weeks and 2 follow-up hospital visits.  C= standard antenatal care. | At 10–15, 28, 34 weeks’ gestation. |
| Koivusalo *et al.* (2016)[36] | Previous history of GDM and/or a pre-pregnancy BMI of ≥ 30 kg/m^2^. | I= individualised intervention on diet, physical activity and weight control (3 sessions) provided by trained study nurses plus one group meeting with a dietitian.  The dietary advice was based on contemporary Nordic Nutrition Recommendations and focused on increasing the intake of vegetables, fruits and berries, whole grain products rich in fibre, low-fat dairy products, vegetable fats high in unsaturated fatty acids, fish and low-fat meat products, while reducing the consumption of sugar-rich foods. A minimum of 150 min of weekly moderate-intensity exercise was recommended. Women had free access to public swimming pools and/or weekly guided exercise groups provided by the municipalities.  C= standard antenatal care involved general advice on diet and physical activity. | At 13.3, 23.1 and 35.1 weeks’ gestation. |
| Korpi-Hyovalti *et al.* (2011)[37] | One or more risk factors: age >40 years, BMI >25 kg/m^2^, family history of DM in a first- or second-degree relatives, previous history of GDM or macrosomic infant >4.5 kg or venous plasma glucose concentration after 12 hr fasting 4.8-5.5 mmol/l and 2-hr OGTT plasma glucose <7.8 mmol/l | I= diet and exercise counselling according to the dietary recommendations of the Diabetes and Nutrition Study Group of the European Association for the Study of Diabetes and the Finnish Diabetes Prevention Study (6 sessions with a nutritionist and 6 with a physiotherapist).  Diet composition: 50–55% carbohydrates, 15–20% protein, 30 % fat, <10% saturated fat and 15 g/1000 kcal fibre. Energy intake: 30 kcal/kg/day for recommended weight women and 25 kcal/kg/day for overweight women. Women were advised to perform 30 min of daily physical activity if they previously exercised <2.5 hr per week and 45 min activity if the women already engaged in ≥2.5 hr per week of physical activity. Women were offered aerobic/aquafit weekly classes.  C (Close follow-up group) = standard antenatal care in addition to a single session which involved general advice on diet and physical activity. | Started at 8–12 weeks’ gestation until 28 weeks’ gestation. |
| Kong *et al.* (2014)[38] | Pre-pregnancy BMI ≥25 kg/m^2^ | I= unsupervised walking program (1 training session).  Guidance on the safety of using a treadmill was provided. The 2008 US physical activity guidelines were discussed verbally (at least 150 min of moderate exercise during pregnancy divided throughout the week). Women were advised to start walking for 50 min in week 1, followed by 100 min in week 2 and 150 min in week 3. Participants were also allowed to walk in shorter bouts (at least 10 min/ bout), Treadmills were provided to the women to promote intervention adherence. Participants were given logs to monitor their walks.  C= women were not provided with any exercise recommendations, but there was no restriction to perform any activities during pregnancy. Additionally, logs were given to controls, to report any leisure-time and physical activity during pregnancy. | Started at 12–15 weeks’ gestation until week 35. |
| LeBlanc *et al*. (2022)[39] | Pre-pregnancy BMI ≥27 kg/m^2^ | I= a behavioural weight loss lifestyle intervention (mean 42 telephone sessions; 20–30 min) guided by a health coach, a trained behavioural interventionist.  Women in the intervention group attended an introductory session to review the study goals and the intervention website and were encouraged to lose weight prior to pregnancy by following the DASH diet (without sodium restriction). An individualised energy goal was used based on the Harris-Benedict equation with modifications of energy target and diet plan if pregnancy occurred.  Participant were advised to perform 60 min per day of moderate-intensity physical activity and walk at least 10,000 steps.  C= routine prenatal care included given general information on having a healthy pregnancy. | Before conception (women planning for pregnancy within 2 years received weekly sessions for 6 months and then monthly for 18 months or until end of pregnancy). |
| Lin *et al.* (2020)[40] | At least one risk factor: age ≥35 years, pre-pregnancy BMI ≥25 kg/m^2^, family history of DM, history of PCOS, previous history of GDM | I= structured educational intervention on diet, physical activity and weight control (1 face-to-face session) with an interventionist and continuous educational messages using WeChat public account twice/week.  The dietary advice was based on the China diagnosis and therapy guideline for pregnancy with DM. Women were encouraged to consume vegetables, fruits, high-fiber whole grain products, low-fat dairy products and to avoid foods rich in sugar and saturated fatty acids. Approximately 30 min of moderate-intensity exercise 3-4 times per week was recommended. Advice on weight gain during pregnancy was based on the recommendation of the National Academy of Medicine.  C= usual prenatal care. | Started at <8 weeks’ gestation. |
| Liu *et al*. (2021)[41] | Pre-pregnancy BMI ≥25 kg/m^2^ | I= behavioural lifestyle intervention (1 in-depth counselling session followed by 10 weekly individual phone counselling calls and podcasts) delivered by the interventionist.  The intervention was guided by the Social Cognitive Theory and followed the GWG 2009 IOM and diet and physical activity guidelines for pregnant women. Women were advised to complete 150 min/week of moderate-intensity exercise and consume balanced diet rich in fruits, vegetables and whole grains and low in saturated and trans fats. The “MyPlate Daily Checklist for Moms” approach was used and customised calorie targets were provided. Women also received educational handouts a pedometer and a bathroom scale. The program included plotting of weight on a graph provided during the session, setting lifestyle goals and problem-solving discussion. Additionally, women were invited to join a private Facebook group.  C= standard antenatal care with 6 monthly mailings and 10 weekly podcasts focused on a healthy pregnancy, with no information provided on weight, exercise or diet. | Started at ≤18 weeks’ gestation. |
| Lovvik *et al.* (2019)[42] | PCOS | I= metformin initiated at 1000 mg/day for the first week and increased to 2000 mg/day from the start of the second week up to delivery.  C= identical placebo.  *Women in both groups received diet and lifestyle advice and were asked to take a multivitamin. | Started at the 1^st^ trimester as soon as possible (around 6–12^+6^ weeks’ gestation) to delivery. |
| Luoto *et al.* (2011)[43] | At least one risk factor: age ≥40 years, pre-pregnancy BMI ≥ 25 kg/m^2^, first-degree relative with type 1 or 2 DM, history of GDM or any signs of glucose intolerance or macrosomic infant (≥4.5 kg) in a previous pregnancy. | I= individualised lifestyle intervention on weight management (1 session), physical activity (5 sessions) and diet (4 sessions) delivered by nurses.  Both the physical activity and dietary sessions were based on the model of Laitakari and Asikainen which integrate two central behavioural models, PRECEDE-PROCEED and Stages of Change. Diet composition was based on Finnish dietary recommendations: 25–30% total fat, ≤ 10% saturated fat, 5–10% polyunsaturated fat, 25–35 g/day fibre and <10% saccharose of total energy intake. Women received physical activity counselling which aimed to maintain or increase leisure time physical activity. The minimum recommended amount of leisure time physical activity per week was 800 MET min including light-intensity physical activity. The participants were offered an opportunity to participate in monthly thematic meetings on physical activity including group exercise.  C= routine antenatal care with brief counselling on physical activity. | At 8–12, 16–18, 22–24, 32–34 and 36–37 weeks’ gestation. |
| Matarrelli *et al*. (2013)[44] | Elevated fasting glucose in the 1^st^ or early 2^nd^ trimester (glycaemia ≥ 5.1 mmol/L or 92 mg/dL and ≤ 7.0 mmol/L or 126 mg/dL) | I= MI plus folic acid from trial entry until delivery (4000 mg MI and 400 μg of folic acid, divided into 2 sachets daily, taken with at least a 6 hr interval).  C= placebo (400 μg/day folic acid).  *All participants received dietary advice according to the ADA recommendations and counselling regarding safe physical activities. | NA. |
| McCarthy *et al.* (2016)[45] | Pre-pregnancy or early pregnancy BMI ≥25 kg/m^2^ | I= serial self-weighing and simple dietary intervention delivered by a research midwife (1 session, 30 min). Women were provided with a card listing the booking BMI, the target GWG based on IOM guidelines in addition to weight management advice based on The Australian Guide to Healthy Eating. Women were asked to plot serial weights on the card.  C= standard care included a trial-specific card with the women booking BMI and their target GWG. | Started at <20 weeks’ gestation. |
| Mohsenzadeh-Ledari *et al*. (2020)[46] | At least three metabolic syndrome indices: abdominal obesity or waist circumference ≥ 80 cm for the first half of pregnancy, hypertriglyceridemia ≥ 150mg for the first half of pregnancy, low HDL: <50 mg, high blood pressure >130 on 85 mmHg, high fasting blood glucose >100 mg/dL. | I= nutrition counselling (2 sessions) with a nutritionist along with a motivational interview session for 120 min.  Recommendations were based on the Nutrition Guidelines of the Ministry of Health for Pregnant Women including five groups’ fruits, vegetables, grains, meat, dairy and water per day based on the Pregnant Mothers’ Food Pyramid: 15–20 kcal/kg/day for BMI > 30, 25 kcal/kg/day for BMI 25–30 and 30 kcal/kg/day for BMI between 20-25. The diet contained 20% protein, 30% fat and 50% carbohydrates. Women were offered 3 counselling sessions regarding physical activity including pelvic floor muscle training, bodybuilding, muscle strengthening, stretching, relaxation and walking. Additionally, a pamphlet, an educational booklet and educational CDs were provided along with follow-up phone calls every 10 days to two weeks.  C= routine prenatal care. | At 15–20 and 20–24 weeks’ gestation. |
| Motahari-Tabari *et al.* (2021)[47] | Baseline BMI ≥25 kg/m^2^ | I= Information-Motivation and Behavioural skills model-based counselling (4 face-to-face sessions, ranging from 60-90 min) by a researcher.  Women were given a booklet about appropriate exercise during pregnancy. PowerPoint/image presentations and question and answer methods were applied during the sessions. The counselling sessions focused on: obesity; nutrition during pregnancy; effects of obesity on GDM; performing physical activities during pregnancy and its benefits; diabetes self-care, managing stress in pregnancy and its effect on GDM and the effect of motivation on mental health and continuity of chronic illnesses control.  C= antenatal usual care based on the national antenatal guidelines of “The Iranian national program on safe motherhood, integrated care on mother’s health”. | Started at 12^+1^–16^+6^ weeks’ gestation (the 1^st^ antenatal visit). |
| Okesene-Gafa *et al*. (2019)[48] | Baseline BMI ≥30 kg/m^2^ | Diet I= home-based tailored educational intervention (4 sessions/ messages 3 times weekly) by a community trained health worker with background in pregnancy nutrition.  Women were provided with a HUMBA handbook which included information about healthy nutritious foods, recipes, unhealthy drinks, ways to control cravings and to be physically active. Behaviour change techniques were used including setting "SMARTER" goals, using charts to plot the woman’s weight, providing feedback and positive reinforcement.  Diet C= routine dietary advice with the New Zealand Ministry of Health pamphlets “Eating for healthy pregnant women” and “Healthy weight-gain in pregnancy” without any dietary contribution from the health practitioners.  Probiotics I= probiotics capsules with Lactobacillus rhamnosus GG and Bifidobacterium lactis BB12 (minimum dose 6.5 x10 ^9^ CFU).  Placebo C= identical placebo capsules (1/day) with microcrystalline cellulose and dextrose anhydrate.  *All groups received routine antenatal care. | Started at ≤ 28 weeks’ gestation until delivery. |
| Oostdam *et al*. (2012)[49] | Previous history of GDM or macrosomic infant or family history of DM in a first-degree relative | I= exercise training program (2 exercise sessions/week) during the remaining duration of the pregnancy under the guidance and supervision of a trained physiotherapist.  The exercise intervention consisted of aerobic and strength sessions starting with an introductory session. Women received instructions on how to use the equipments. Each session started with 5-10 min warming-up light-intensity activity, then an individualised program of 40 min, included 1 or 2 aerobic exercises and 4 to 6 strength exercises.  C= normal care from obstetricians and/or midwives. | Started at 15 weeks’ gestation until 12 weeks postpartum. |
| Parat *et al.* (2019)[50] | Pre-pregnancy BMI >25 kg/m^2^ | I= individually tailored intervention (6 sessions; 2 individual face-to-face dietary sessions and 4 collective group education sessions) by a physician (paediatrician or endocrinologist), dietitian, or a midwife.  The program started with an interview (60–90 min) in the first session to establish an education plan. The interview focused on an educational diagnosis of individual knowledge, fears, beliefs and lifestyle. Minimal information was provided on the effect of obesity on pregnancy and fetus development. Quality of life, physical and mental health were assessed. The first session aimed to improve subjects’ knowledge about healthy diet and physical activity benefits. Women were given the national booklet about nutrition during pregnancy. Collective sessions started with the analysis of behaviours, successes and challenges. Then the individually tailored plan was adapted according to their challenges and achieved goals. In the remaining sessions (from 28 weeks’ gestation until 2 months postpartum), the following topics were discussed: the psychosocial approach to pregnancy, weight gain, maternal diet and physical activity, baby’s needs and feeding, baby’s health/weaning and nutrition, postpartum physical activity and relaxation. The two dietary sessions at 26 and 30 weeks’ gestation focused on healthy diet (without any weight goals) and motivation.  C= routine care including at least one face-to-face dietary session at 26 weeks’ gestion included general oral and written information about diet and exercise. Women were provided with the same national booklet. An additional dietitian session was available upon request, or if GDM was diagnosed. | At 21, 26, 28, 30 and 35 weeks’ gestation until 2 months postpartum. |
| Peccei *et al.* (2017)[51] | Baseline BMI between 25-40 kg/m^2^ | I= an intensive individualised nutrition intervention (face-to-face or phone sessions twice/month throughout pregnancy; 10–30 min), with the study registered dietitian.  The program involved individualised meal plans considering cultural food preferences, financial limitations and education. Weights were tracked using a graph and additional counselling topics included label reading, healthy shopping on a budget, nutrients (fibre/vitamins/minerals) recommendations and benefits of breastfeeding.  Diet composition: Energy needs (based on actual weight) were calculated using the Mifflin-St Jeor equation (45% carbohydrates, 25% protein, 30% fat).  C= standard prenatal and postpartum care in addition to the initial study visit.  *Both groups received an initial study visit (60–90 min), discussing the following: pregnancy-related risks of being overweight or obese, IOM GWG recommendation, basic nutritional needs, healthy eating and safe exercise during pregnancy. All women were given a brochure “Gaining Weight During Pregnancy” and a booklet entitled “Thumbs Up for Healthy Food Choices”. Women were advised to increase the intake of fruits, vegetables and whole grains while selecting lean protein, low-fat dairy and healthy fat products. They were encouraged to swap high-calorie sugary drinks with water or other low-calorie drinks and limit “discretionary” calories such as unhealthy desserts. Food safety and fish intake recommendations were discussed. 30 min walking was recommended on most days and tracking steps using the provided pedometer. | Started at <16 weeks’ gestation. |
| Pellonperä *et al.* (2019)[52] | Pre-pregnancy BMI ≥25 kg/m^2^ | Fish oil + placebo I= two fish oil capsules daily with a total of 2.4 g of omega-3 fatty acids (1.9 g DHA, 0.22 g of EPA and the remaining from other omega-3 fatty acids including docosapentaenoic acid) plus the “fish oil/placebo”.  Probiotics + placebo I= one probiotic capsule with Lactobacillus rhamnosus and Bifidobacterium animalis subspecies lactis 420, 1 X 10^10^ CFU/per day plus the “probiotics/placebo”.  Fish oil + probiotics I= two fish oil capsules and one probiotic capsule (as described above).  Placebo + placebo C= identical fish oil/placebo capsules with an equal amount of medium-chain fatty acids (capric acid C8 and caprylic acid C10) and identical probiotics/placebo capsules contained microcrystalline cellulose.  *All women attended two study visits for the assessment and capsule supplies.  *All women were asked not to consume other probiotics and omega-3 fatty acids products during the study. | Started at randomisation (<18 weeks’ gestation) until 6 months postpartum. |
| Petrella *et al. (*2013)[53] | Pre-pregnancy BMI ≥25 kg/m^2^ | I= therapeutic lifestyle changes diet (1 counselling session; 1-hr) with the supervision of both a gynaecologist and a dietitian.  Diet composition: 1700 kcal/day (obese) or 1800 kcal/day (overweight), 55% carbohydrates (at least 225 g/day of which 80% complex with low-glycaemic Index and 20% simplex), 20% protein (50% animal and 50% vegetable), 25% fat (12% monounsaturated, 7% polyunsaturated and 6% saturated).  Women were advised on the ideal GWG in order to prevent adverse outcomes.  The intervention aimed at decreasing high-GI foods consumption and swapping them with healthier substitutes and redistribution of meals throughout the day. Small frequent meals (3 main meals and 3 snacks) were recommended to avoid ketonuria and acidosis.  Women were asked to follow a more active lifestyle based on the recommendations for the general population and were instructed to engage in 30 min of moderate-intensity exercise at least 3 days /week. To monitor the exercise, women were provided with a pedometer and were advised to consider using the ‘‘talk test’’.  C= no intervention. Only received a general lifestyle booklet in accordance with Italian Guidelines for a healthy diet during pregnancy. | Started at randomisation; during 12 weeks’ gestation. |
| Phelan *et al.* (2018)[54] | Baseline BMI ≥25 kg/m^2^ | I= a behavioural lifestyle intervention with a structured partial meal replacement plan (individual face-to-face sessions every 2 weeks until 20 weeks’ gestation and then monthly visits until delivery) provided by the study interventionist including bilingual registered dietitians or counsellors with specific health degrees.  The intervention was established according to social learning theory and hypothesised pregnancy as a “teachable moment” for behaviour modification. Women were required to follow the 2009 IOM guidelines. Additional visits with the interventionist (2 visits/month) were offered for some women to support a return to the recommended GWG. An individually tailored meal plan was provided to meet each woman's self-reported dietary needs.  Diet composition: ∼18 kcal/kg (baseline body weight), 50–55% carbohydrates, 30% fat, 15–20% protein.  Women were offered free meal replacement products and advised to replace 2 meals with the provided shakes or bars and to consume ≥1 meal of regular foods and 2–4 healthy snacks/day. The meal replacement options were chosen according to the current micronutrient and macronutrient recommendations for pregnant women. Options such as organic and lactose-free drinks and bars were available.  On most days of the week, 30 min of exercise was recommended. A pedometer was given, and women were asked to gradually increase the number of steps per day until achieving a goal of 10,000 steps/day.  Additionally, a personalized graph of GWG was provided with feedback. The intervention included behavioural strategies such as diet/exercise records, stimulus control techniques, problem-solving skills, goal setting, self-reinforcement and self-monitoring of weight using a provided scale.  C= enhanced usual care adopted from the Women, Infants and Children’s Special Supplemental Nutrition Program. Controls attended a welcome visit with a study interventionist (∼20 min), who provided general information about healthy diet, exercise and the IOM recommendations for total GWG. At 2 months, women received trial newsletters with simple information about pregnancy-related health (e.g., vitamins, smoking, breastfeeding, maternity clothes). | Started at 13 weeks’ gestation on average. |
| Phelan *et al.* (2023)[55] | Baseline BMI ≥25 kg/m^2^ and previous GDM | I= a lifestyle modification program (face-to-face/phone /video 16 weekly sessions then sessions every 2 weeks until conception; 30 min) delivered by a lifestyle interventionist.  The program aimed for a 10% weight loss then maintain weight loss until conception. The intervention contacts were ceased after conception. Women were advised to consume a standard calorie-restricted, nutritionally balanced diet and increase their physical activity to at least 150 min/week.  Diet composition: 45% carbohydrates, 35 % fat, and 20% protein.  C= standard care in addition to general education about preconception health. | Before conception (women planning for pregnancy within 1-3 years received 16-week pre-pregnancy intervention ongoing until conception). |
| Phillips *et al.* (2019)[56] | Baseline BMI 25-29.9 kg/m^2^ or obese BMI ≥30 kg/m^2^ | I= a combined financial incentive and individual weight management intervention every 2 weeks throughout the pregnancy (12 sessions; 30 min) with the support of a behaviourally trained nutritionist.  Participants were instructed to follow US Dietary Guidelines and the US Department of Agriculture My Plate Program. daily calorie goal was set based on BMI and exercise level. During the program, counselling on GWG based on the IOM recommendations was provided along with individualised teaching in line with principles of behavioural weight management (self-monitoring, stimulus control, problem solving, motivation, feedback and social support).  Physical activity was encouraged but was not a specified component of the intervention. Successful women were offered up to $550 as incentives.  C= standard obstetric care with lifestyle recommendations according to the ACOG. | Started at ≤16 weeks’ gestation. |
| Poston *et al.* (2015)[57] | Baseline BMI ≥30 kg/m^2^ | I= individualised behavioural intervention (face-to-face/telephone/email group or individual 8 sessions; 1-hr) delivered by a health trainer.  The intervention was informed by control theory and elements of social cognitive theory and started with an individual interview. Each session addressed strategies to achieve the "SMART" goals and review the previous goals. The intervention covered the following: self-monitoring, problem-solving; enlisting social support; and providing opportunities for social comparison. Women were provided with a trial specific handbook, a DVD, a pedometer and a logbook.  The dietary component of the intervention focused on promoting a healthy eating without restricting energy intake. The advice was tailored based on the woman’s habitual diet and cultural preference. Women were recommended to swap carbohydrate-rich foods with a medium-to-high-GI foods and restrict the consumption of saturated fat.  The physical activity component aimed to promote incremental increases in walking at a moderate intensity tailored to pre-existing activities. Additional exercise options were provided for women who are already physically active.  C= standard antenatal care based on local practice which included general information regarding healthy eating and the benefits of physical activity. | Started at 15–18^+6^ weeks’ gestation (within 1 week of randomisation). |
| Quinlivan, Lam and Fisher. (2011)[58] | Baseline BMI ≥25 kg/m^2^ | I= four-step multidisciplinary antenatal care in a trial-specific antenatal clinic.  The program included the following:  1-continuity of care by a single maternity care provider.  2- assessment of weight gain  3- a brief dietary intervention (5 min) by a food technologist.  4- evaluation by a clinical psychologist and individualised solution-focused therapy plan where appropriate.  C= routine public antenatal care. | Started at 1^st^ antenatal visit. |
| Rakhshani *et al*. (2012)[59] | At least one risk factor: age <18 or ≥35 years, baseline BMI ≥30 kg/m^2^, twin pregnancies, family history of poor obstetrical outcomes, maternal history of poor obstetrical outcomes in previous pregnancies (GDM, PIH, pre-eclampsia, PROM, SGA, LGA, IUGR, low Apgar score, preterm deliveries) | I= yoga interventions directed by well-trained certified yoga therapists (28 sessions, one hr/ 3 times a week),  The program focused on three interventions:1) breathing exercises, 2) yogic postures and 3) meditative trainings.  The meditative exercises consisted of meditation, guided imagery and sound resonance methods. Women were requested to imagine the fetus in the uterus and the blood passing from their bodies across the placenta to provide nutrients to the growing fetus.  C= standard antenatal care plus walking (half an hour mornings and evenings).  *Women in both groups received: 1) pamphlets about diet and maternal nutrition; 2) routine check-ups 3) biweekly follow-ups by the research team. | Started at 13 until 28 weeks’ gestation. |
| Renault *et al*. (2014)[60] | Pre-pregnancy BMI ≥30 kg/m^2^ | Physical activity I= individual physical activity intervention delivered by the dietitian. The goal was 11,000 steps/day as monitored by a validated pedometer.  Diet + physical activity I= The same physical activity intervention plus a dietary intervention with an experienced dietitian every 2 weeks (face-to-face or phone 11-13 sessions).  C= usual hospital standard care for pregnant women with obesity  *Women in the three groups were seen by a dietitian at the initial ultrasound nuchal scan. An individual consultation was offered (only oral advice without a meal plan) which included a recommendation of a hypocaloric low-fat Mediterranean-style diet (1200-1675 kcal) in line with the Danish national recommendations for healthy eating. | Started at 11–14 weeks’ gestation. |
| Roeder *et al.* (2019)[61] | HbA1c 5.7 - 6.4% and/or FPG 92 - 125 mg/dL | I (1^st^-trimester group)= nutrition and exercise counselling (1 session), glucose monitoring and medication as needed by a certified diabetes educator. Women were seen every 2 to 4 weeks by their prenatal provider. Carbohydrates intake goal: 15-g breakfast, 30- to 45-g lunch/dinner and 15-g snacks between meals and at bedtime.  C (3^rd^-trimester group)= routine prenatal care until 28 weeks’ gestation.  *Women in both arms received the same intervention and treatment, but at a different gestational age at the initiation of the intervention. | Started at ≤ 15^+0^ weeks’ gestation. |
| Sadiya *et al*. (2022)[62] | At least two risk factors: pre-pregnancy BMI ≥30 kg/m^2^, first-degree relative with DM, (high-risk ethnicity (Middle Eastern, Southern Asian), history of PCOS, previous history of GDM or macrosomic infant > 4.5 kg. | I= a 12-week moderate-intensity lifestyle intervention (2 face-to-face sessions; ≈ 30–45 min, with telephone follow-ups conducted between the sessions) led by a licensed dietitian.  The intervention was provided based on the ADA and followed the GWG 2009 IOM and women were trained on "SMART" goals approach, problem-solving skills and self-monitoring. The dietary component focused on increase the intake of whole grains, vegetables, fruits and portion while lowering the intake of ultra-processed food and simple sugars.  Diet composition: 50–55% carbohydrates, 25–30 % fat, and 20% protein.  The women were encouraged to perform 150 min per week of moderate-intensity physical activity or to monitor a minimum of 10,000 steps/day (1 hr and 40 min/ day).  C= standard antenatal care with general advice regarding lifestyle changes. | Started at the 1^st^ trimester (around 6–12 weeks’ gestation). |
| Sales *et al*. (2018)[63] | Baseline BMI ≥30 kg/m^2^ | I= metformin dose of 1000 mg twice daily (500 mg at breakfast and 500 mg at dinnertime).  C= prenatal care according to the primary protocol recommended by the Brazilian Ministry.  *Women in both groups received nutritional care. Dietary guidance included a lower caloric intake of 24 kcal/kg/day and 5-6 daily meals. Diet composition: 40–50% fiber-rich complex carbohydrates, 20% protein, 30–40% of unsaturated fats. Caloric distributions: 10–20% at breakfast, 20–30% at lunch, 20–30% at dinner and up to 30% as snacks. Recommendations for exercise were provided by physical therapy which included a regular walking program of 20 min/day. | Started at ≤ 20 weeks’ gestation. |
| Santamaria *et al*. (2016)[64] | Baseline BMI between 25-30 kg/m^2^ | I= MI plus folic acid (2 g MI and 200 μg folic acid twice a day).  C= placebo (200 μg folic acid twice a day). | Started at the 1^st^ trimester (around 12–13 weeks’ gestation) to delivery. |
| Sartorelli *et al*. (2022)[65] | Pre-pregnancy BMI between 25.0–29.9 kg/m^2^ | I= individualised nutritional counselling (3 face-to-face sessions; ≈ 30 min) conducted by trained nutritionists.  Women were educated about the following goals of the nutritional approach:   1. Appropriate weight gain, 2. Intake of unprocessed and minimally processed foods such as fruits and vegetables. 3. Regular exercise (150 min per week). 4. Avoiding the consumption of ultra-processed products.   The intervention was provided based on the recommendations of the Institute of Medicine, Food Guide for the Brazilian Population and ACOG. Women were provided with educational material (3 folders include key messages and illustrative images).  C= usual prenatal care which includes one counselling section with the hospital nurse to evaluate the nutritional status, focus on healthy weight gain, detect dietary inadequacies and clarify misconceptions. | Started at ≤ 16 weeks of gestation |
| Seneviratne *et al.* (2016)[66] | Baseline BMI ≥25 kg/m^2^ | I= a 16-week structured home-based moderate-intensity antenatal exercise program (67 prescribed sessions) led by an exercise physiologist.  Women were required to use magnetic stationary bicycles and were given written materials with the frequency (3-5 sessions/week) and duration (15-30 min/session) of weekly exercises. A home visit by the physiologist was scheduled for each woman to solve any exercise-related problems. To maintain moderate-intensity exercises and achieve target heart rates, heart rate monitors were provided.  C= no exercise intervention with routine antenatal and delivery care.  *Women in both groups were allowed to remain following their routine physical activity and diet without restriction. | Started at ≤ 20 until 35 weeks’ gestation. |
| Shahgheibi, Farhadifar and Pouya. (2016)[67] | At least one risk factor: age ≥35 years, pre-pregnancy BMI ≥25 kg/m^2^, family history of DM/GDM, previous history of GDM or macrosomic infant and glycosuria. | I= vitamin D (5000 IU per week).  C= placebo. | Started at the 1^st^ /2^nd^ trimester until 26 weeks’ gestation. |
| Shahriari *et al*. (2021)[68] | At least one risk factor: age >35 years, insulin resistance disorders such as PCOS, previous history of GDM or macrosomic infant/ fetal anomaly, recurrent abortion and intrauterine fetal death. | I= mixture of probiotics (1 capsule of Lactobacillus acidophilus LA1 (> 7.5 X 109 CFU), Bifidobacterium longum sp54 cs (1.5 X 109 CFU) and Bifidobacterium bifidum sp9 cs (> 6 X 109 CFU)) taken with a glass of water or milk.  C= identical placebo (one capsule with 500 mg starch and maltodextrins) taken with a glass of water or milk.  *Women in both groups received weekly text and phone calls about the importance of probiotic and were asked not to change their lifestyle during the trial or consume probiotic-containing products. | Started from the first half of the 2^nd^ trimester (14 up to 24 weeks’ gestation). |
| Simmons *et al*. (2017)[69] | Pre-pregnancy BMI ≥29 kg/m^2^ | *All the interventions were delivered by a lifestyle coach (5 face-to-face sessions; ≈ 30–45 min, with 20 min telephone calls or email contacts) with restriction in GWG to 5 kg using motivational interviewing and cognitive behavioural techniques.  Diet I= the coaching involved a discussion of 7 diet messages. Dietary guidance included lower intake of calories, fat and simple and complex carbohydrates. Women were encouraged to consume more fibre and protein with a focus on portion size.  Physical activity I= the coaching involved a discussion of 5 physical activity messages and recommended aerobic and resistance exercise based on the ACOG.  Diet + physical activity I= combined diet and physical activity programs.  C= usual care. | Started at 8 –19 ^+6^ until 35 weeks’ gestation. |
| Syngelaki *et al.* (2016) [70] | Baseline BMI ≥35 kg/m^2^ | I= metformin taken with meals, initiated at (1.0 g) daily for the first week and increased by 0.5 g per week to achieve the maximum tolerable dose of 3 g over five weeks period.  C= matched placebo tablets taken with meals.  *Women in both groups received standardised personal advice on healthy diet, with a focus on low-GI foods and were instructed to exercise for 30 min/day. | Started at 12–18 weeks’ gestation until delivery. |
| Thornton *et al*. (2009)[71] | Baseline BMI ≥30 kg/m^2^ | I= active nutritional and behavioural intervention.  The intervention was based on the GDM dietary guidelines. Women were asked to record dietary intake to be reviewed by the physician at each antenatal visit. To avoid any influence on diet intake, women were requested not to weigh themselves at home.  Diet composition:18–24 kcal/kg energy (>2000 kcal/day), 40% carbohydrates, 30% protein and 30% fat.  C= conventional dietary prenatal management.  *Women in both groups were informed about conventional prenatal nutrition guidelines by a registered dietitian (at least once) and were encouraged to walk for 30 min/day. | Started at 12–28 weeks’ gestation. |
| Valdés *et al*. (2018)[72] | PIR (based on abnormal fasting insulinemia test (≥15 uUI/mL) and/or homeostatic model assessment–insulin resistance (>2.6) with at least one of PIR-suggestive clinical signs, such as acrochordons, acanthosis nigricans or the diagnosis of PCOS) | I= metformin (1700 mg/day).  C= placebo. | Started at 12^+0^–15^+6^ until 36 weeks’ gestation. |
| Van Horn *et al.* (2018)[73] | Pre-pregnancy BMI between 25–40 kg/m^2^ | I= Technology-enhanced DASH diet and lifestyle intervention (6 group sessions; 30 min) guided by a registered dietitian nutritionist.  The intervention focused on three aspects: diet, physical activity and sleep.  The DASH diet focused on increasing the consumption of low-fat dairy products, lean meat and plant-based protein, unsaturated fats, whole grains, fruits, vegetables and legumes while reducing the intake of sugar-sweetened beverages and non-nutrient-dense snacks. A minor modification to restrict total GWG was introduced to the diet “MAMA-DASH”. This includes avoidance of high mercury fish and encouragement of calcium-rich, vitamin D−enriched dairy, or calcium-fortified non-dairy foods. During the sessions, the following topics were covered: enriching meals with fruit and vegetables, food labels, eating out, whole grain options, fish selection, cooking methods and breastfeeding. Weekly progress reports (based on the woman’s intake) were reviewed and new/revised goals were set. Educational resources about MAMA-DASH lifestyle (e.g., recipes, fitness tips) were e-mailed weekly. Sessions records were available for those who were unable to attend.  Women were encouraged to participate in >30 min of activity or walking (>10,000 steps/day) and were provided with pedometers to monitor activity. A daily sleep duration of 7–9 hr was recommended.  C= Usual care. Women were provided with access to the MOMFIT website, including the Department of Agriculture’s U.S. Dietary Guidelines and pregnancy recommendations from the ACOG. In addition, the controls received biweekly electronic newsletters about maternal and infant care.  *Women in both groups received the 2008 Physical Activity Guidelines for Americans and recommendations from the ACOG. | Started at 16 weeks’ gestation. |
| Vanky *et al*. (2004)[74] | PCOS | I= metformin initiated at 850 mg/day for the first week and increased to 1700 mg/day.  C= identical placebo.  *Women in both groups received written and verbal counselling on diet and lifestyle and were asked to take a multivitamin. | Started at 5–12 weeks’ gestation. |
| Vanky *et al*. (2010)[75] | PCOS | I= metformin initiated at 1000 mg/day for the first week and increased to 2000 mg/day from the start of the second week up to delivery.  C= identical placebo.  *Women in both groups received written and verbal counselling on diet and lifestyle and were asked to take a multivitamin. | Started at 1^st^ trimester until delivery. |
| Vesco *et al*. (2014)[76] | Baseline BMI ≥30 kg/m^2^ | I= a combination of dietary and exercise recommendations (16 group sessions/ 90 min) with the use of behavioural self-management techniques led by a dietitian and intervention leaders.  The intervention included two initial individual counselling visits designed to help tailor the diet and physical activity program to the woman’s requirements. The diet was an energy-reduced version of the DASH pattern without sodium restriction. A specific formula was used for calorie goals [(pre-pregnant weight in kg) (30 kcal/kg/day) (0.70)]1[(10 kcal) (gestational age in weeks)].  At least 30 min/day of moderate physical activity was recommended based on the ACOG. Women were offered a pedometer and encouraged to keep activity records. Both food and physical activity diaries were monitored weekly.  Behavioural self-management principles were used, this include setting reasonable short-term goals, creating action plans and identifying sources of reinforcement and social support. Women were encouraged to discuss challenges in behavioural changes, ask questions and solve the problems/ barriers in group discussions.  C= routine medical care in addition to a one-time advice session delivered by the dietitian which involved general information about healthy maternal diet, without particular emphasis on the DASH diet or weight management. Women received feedback about their food diaries and were asked to follow their antenatal care providers’ guidance. | As early as possible ranging from 7–21 weeks’ gestation. |
| Vinter *et al*. (2011)[77] | Baseline BMI between 30-45 kg/m^2^ | I= lifestyle intervention consisted of dietary counselling performed by dietitians (4 sessions) and training classes with physiotherapists (1 hr/week).  The dietary advice was based on the official Danish recommendations. Energy requirements were individually estimated based on weight and exercise level.  Participants were instructed to engage in 30-60 min/day of moderate-intensity exercise and were equipped with a pedometer to improve performance. Women were also offered a free gym membership for 6 months. Training included: aerobic (low step), light weights, elastic bands and balance exercises. Coaching-inspired methods were used (4–6 group sessions) to improve the physical activity during pregnancy.  C= women received the same initial information about the aim and content of the trial and had access to a website with general advice about maternal diet and exercise without additional intervention. | At 15, 20, 28 and 34–36 weeks’ gestation. |
| Vitale *et al*. (2021)[78] | Pre-pregnancy BMI between 25 and <30 kg/m^2^ | I= MI plus folic acid (2 g MI and 200 mg folic acid twice a day).  C= placebo (200 mg folic acid twice a day).  *All women followed the same diet based on the ADA recommendations. | Started around 12–13 weeks’ gestation until three weeks after delivery. |
| Walsh *et al*. (2012)[79] | Previous history of macrosomic infant >4 kg. | I= a low-GI diet intervention in a group of 2–6 participants (1 session) and 2 follow-up sessions with the study dietitian.  Women were advised to choose as many low-GI foods as possible and to exchange high-GI carbohydrates for low-GI alternatives. Women received written resources about low-GI foods. The recommended diet was eucaloric and women were not advised to reduce their total energy intake. The follow-up sessions focused on reinforcing of the low-GI diet and answering any dietary questions.  C= routine antenatal care. | At 12–16, 28 and 34 weeks’ gestation. |
| Wang *et al*. (2017)[80] | Pre-pregnancy BMI ≥24 kg/m^2^ | I= a supervised cycling program at least (3 sessions/week) by a supervisor.  The exercise protocol for each session included stationary cycling for 30 min (progressively increased to 45-60 min), starting with a 5 min warm-up at low intensity, then 5 min of continuous moderate-intensity cycling to achieve target heart rates. The next step was multiple periods of interval cycling consisting of 30 sec of rapid pedalling (sprints, higher intensity efforts) followed by 5 min of continuous cycling at low-to-moderate intensity.  C= standard prenatal care and were continued with their usual daily exercises.  * Women in both groups received general advice about the health benefits of physical activity during pregnancy without providing any dietary recommendations. | Started within 3 days of randomisation (≈10 weeks’ gestation) and continued to the end of the 3^rd^ trimester |
| Wang *et al*. (2015)[81] | At least one risk factor: age ≥35 years, pre-pregnancy BMI ≥25 kg/m^2^, family history of DM, history of PCOS, previous history of GDM or macrosomic infant. | I= standardised two-step lifestyle intervention based on the dietary pagoda of pregnant women in China. The intervention included 3 group courses (delivered in 2 sessions) instructed by one physician.  The intervention focused on various diet and physical activity topics including: “What is a balanced diet”, “Proper physical activity is beneficial during pregnancy” and “Standard weight-gain during pregnancy”. For the physical activity component, women were advised to walk for 30 min at least once a day.  C= routine antenatal care. | At 6–8 and 12–13 weeks’ gestation. |
| Wolff *et al*. (2008)[82] | Baseline BMI ≥30 kg/m^2^ | I= dietary intervention (10 sessions, 1hr) delivered by a trained dietitian.  Individualised healthy eating plans were designed according to the woman’s food records.  Diet composition was based on the official Danish dietary recommendations: 50–55% carbohydrates, 30% fat and 15–20% protein. Energy requirements were determined individually considering the woman’s needs and estimated energetic cost of fetal growth (energy requirement= basal metabolic rate x1.4 (physical activity level factor of 1.2 +0.2)).  C= routine care without any dietary consultations or restrictions on energy intake or GWG.  *All women received dietary supplements | Started at 15^+3^ weeks’ gestation. |
| Zhang *et al*. (2019)[83] | Baseline BMI ≥24 kg/m^2^ | I= a standard nutrition and physical activity consultation (3 sessions) with additional low-GI and GL recommendations by a dietitian.  A mobile phone app ‘DietGI’ was used to help in selecting food quantity and quality and identification of food GI and GL. Women were advised to replace high-GI foods with low-GI varieties and were educated about food combinations and cooking techniques to reduce GI.  C= routine antenatal care with a standard nutrition and physical activity consultation (3 sessions) by a dietitian.  *Women in both arms received a standard dietary consultation included individualised assessment, followed by a meal plan based on the national recommendations of the Chinese Nutrition Society and were advised regarding GWG in accordance with 2009 IOM guidelines. Follow-up calls were provided at least once a month. | Started at ≤16 weeks’ gestation (1^st^ antenatal visit). |
| Zhang *et al*. (2022)[84] | Pre-pregnancy BMI ≥24 kg/m^2^ | I= 1 bag (12 g) of soluble fiber powder twice daily which contained 51.93 of kcal energy, 3.31 g of carbohydrates and 9.78 g of dietary fiber.  C= standard prenatal care.  Both groups maintained 24-hr food recalls and received nutrition education and dietary advice by nutritionists based on the Chinese Dietary Guidelines for Pregnant Women. | Started from 20 until 24^+6^ weeks of gestation. |

ADA= American Diabetes Association; ACOG= American College of Obstetricians and Gynecologists; ACSM=American College of Sports Medicine; BMI= body mass index; BP= blood pressure; CFU= colony forming units; C= control; DASH= dietary approaches to stop hypertension; DCI= D-chiro-inositol; DM= diabetes mellitus; DPP= Diabetes prevention program; FPG= fasting plasma glucose; GDM= gestational diabetes mellitus; GI= glycaemic index; GWG= gestational weight gain; HbA1c= haemoglobin A1c; hr= hour; IUGR= intrauterine growth restriction; I= intervention; IOM= institute of medicine; LGA= large-for-gestational-age; MET= metabolic equivalent minutes; MI= myo-inositol; NA= not applicable; OGTT= oral glucose tolerance test; PCOS= polycystic ovary syndrome; PIH= pregnancy induced hypertension; PIR= pregestational insulin resistance; PROM= premature rupture of membranes; SGA= small-for-gestational age; T2DM= type 2 diabetes mellitus; USDA= United States Department of Agriculture.

**Table A6.** Pregnancy outcomes

| Reference | GDM outcome | Additional significant results |
| --- | --- | --- |
| Ajmani and Sircar (2020)[1] | The incidence of GDM in the intervention group was lower than in the control group (n=12, 13.95% vs. n=30, 32.61%; OR 0.34, 95% CI 0.158–0.709, *p*<0.001) (**IADPSG/ADA**). | NA. |
| Amaefule *et al.* (2022)[2] | No difference in GDM. | There was a reduction in HOMA-IR in the intervention group (MD−0.6; 95% Cl 1.2 −0.0, *p=*0.05). |
| Al Wattar *et al.* (2019)[3] | The odds of GDM reduced by 35% the intervention group (n=84, 17.6% vs. n=124 24.9%; aOR 0.65, 95% CI 0.47–0.91, *p*=0.01) (**IADPSG**). | The intervention group gained less weight during pregnancy (MD−1.2 kg, 95% CI 2.2−0.2, *p*=0.03).  There was an increase in the consumption of nuts, olive oil, fish and pulses and a reduction in the intake of red meat, butter, margarine and cream in the intervention group (all *p*<0.05). |
| Basu *et al*. (2021)[4] | No difference in GDM. | Maternal weight gain was lower in the intervention group at the end of the trial (6.8 ± 3.2 kg vs. 12.0 ± 4.1 kg, *p=*0.001).  C-reactive protein was also lower in the intervention group (baseline, midpoint, end, *p=*0.002).  Blood glucose based on GCT was lower in the intervention group (100 ± 33 mg/dL vs. 131 ± 40 mg/dL, *p*<0.05).  Caesarean delivery rates were lower in the intervention group (n=3 vs. n=10, *p=*0.03).  Intake of carbohydrates was lower while intake of protein was higher in the intervention group (all *p*<0.05). |
| Begum *et al*. (2009)[5] | A nine-fold reduction in GDM rate was seen in the intervention group compared to controls (n= 1, 3.44% vs. n= 9, 30%, OR 12, 95% CI 6.20–18.08). | Birthweight was lower in the intervention group compared with the control group (*p*=0.016).  Apgar score was lower in the control group (*p*< 0.05). |
| Bisson *et al*. (2015)[6] | No difference in GDM. | The exercise group had higher physical activity levels following the intervention (*p=*0.020) and at 36 weeks of gestation (*p=*0.034) and gained less weight than controls during the intervention period despite similar nutritional intakes (MD− -0.1 kg/week, 95% CI -0.2–-0.02, *p=*0.016) and improved cardiorespiratory fitness (*p=*0.041). |
| Brink *et al*. (2018)[7] | No difference in GDM. | NA. |
| Bogaerts *et al*. (2013)[8] | No difference in GDM. | Women in the intervention groups (brochure and lifestyle intervention) had less GWG compared to the controls (brochure 9.5 ± 6.8 kg; lifestyle group 10.6 ± 7 kg vs. controls 13.5 ± 7.3 kg, *p*=0.007).  Levels of anxiety significantly decreased in the lifestyle intervention group and increased in the control group during pregnancy (*p*=0.02). |
| Bruno *et al.* (2017)[9] | The occurrence of GDM was lower in the intervention group compared to controls (n= 13, 18.8% vs. n= 23, 37.1%, *p=*0.019) (**IADPSG**). | The rates of PIH, preterm delivery, induction of labour, LGA babies and birthweight>4000 g were lower in the intervention group (all *p*<0.05).  The diet adherence score was higher in the intervention group (*p=*0.028). |
| Callaway *et al*. (2010)[10] | No difference in GDM. | NA. |
| Callaway *et al*. (2019)[11] | No difference in GDM. | Fasting glucose at OGTT was higher in the probiotics group compared with placebo (79.3 mg/dL vs 77.5 mg/dL, *p=*0.049). Rates of SGA were higher in the placebo group (2.4% vs. 6.5%, *p=*0.042). |
| Celentano *et al*. (2018)[12] | The intervention groups showed a reduction in GDM (RR 0.083, 0.559 and 0.621 for MI, DCI and MI/DCI groups respectively, *p*<0.05) (**IADPSG**). | Fasting, 1-hr and 2-hr glucose values were lower in the intervention groups (*p*<0.001,0.011 and 0.037).  Women who received MI alone had a lower incidence of abnormal OGTT (n=2, 5.1% vs. n=32, 61.5% in C, n=11, 34.4% in DCI and n=13, 38.2% in MI/DCI, *p*<0.001).  Birthweight was lower in the MI and MI/DCI groups compared with the control group (3238 ± 371g in MI, 3223 ± 583g in MI/DCI vs. 3361 ± 521g in C, *p*=0.047). |
| Chan *et al*. (2018)[13] | No difference in GDM. | Total energy intake was reduced in the intervention group (*p*=0.001). |
| Chiswick *et al.* (2015)[14] | No difference in GDM. | NA. |
| Corcoy *et al*. (2020)[15] | No difference in GDM. | NA. |
| D’Anna *et al*. (2013)[16] | GDM was reduced by 65% in the MI group compared with the control group (6% vs 15.3%: OR 0.35, 95% CI 0.13–0.96, *p*=0.04) (**IADPSG**). | The MI group had a lower incidence of abnormal OGTT; both FBG (*p*=0.001) and 1-hr glucose values (*p*=0.02), compared with the control group.  Infants’ birthweight was lower in the MI group (3111 ± 447g vs. 3273 ± 504 g, *p*=0.018). |
| D’Anna *et al.* (2015)[17] | The intervention group showed a reduction in GDM (n= 15, 14% vs. n=36. 33.6%, *p*=0.001) (**IADPSG**). | Women in the intervention group had lower Insulin resistance (as assessed by HOMA-IR) compared to the control group (*p*=0.048). |
| Daly *et al*. (2017)[18] | No difference in GDM. | Excessive GWG at 36 weeks of gestation was lower in the intervention group (23.5% vs 45.2% *p*<0.05). |
| Deng *et al.* (2021)[19] | The incidence of GDM in the intervention group was lower than in the control group (23.90% vs. 51.10%, χ2 = 7.301, *p=*0.007) (**ADA, 2020**). | The incidence of c-section in the intervention group was higher compared to the control group (58.5% vs. 27.9%, χ2 = 8.04, *p*=0.005). |
| Ding *et al*. (2021)[20] | GDM was reduced in the intervention group (n= 25, 24.0% vs. n= 42, 37.8%, χ2 = 4.77, *p*=0.029) (**IADPSG**). | The intervention group had lower GWG during the whole gestation period (11.2 ± 4.9 kg vs. 13.4 ± 5.0 kg, *p*<0.001). |
| Dodd *et al*. (2019)[21] | No difference in GDM. | Women in the intervention group had lower average weekly GWG (adjusted MD –0.08 kg, 95% CI -0.14 – -0.02, *p*=0.007). |
| Dodd *et al*. (2014)[22] | No difference in GDM. | The intervention group were less likely to have LGA infants (>4000 g) (n=164, 15% vs. n=201, 19%, *p*=0.04). |
| Eslami *et al*. (2018)[23] | No difference in GDM. | There was an increase in the weakly consumption of vegetable and fruit and a reduction in the intake of fats, oils and confections in the intervention group (all *p*<0.05). |
| Esmaeilzadeh *et al*. (2022)[24] | The incidence of GDM in the intervention group was lower than in the control group (11.1% vs. 37.9%, RR 0.29, 95% CI 0.09–0.94, *p=*0.038) (**ADA, 2011**). | Women in the intervention group had experienced less inappropriate GWG (RR 0.57, 95% CI 0.35–0.95, *p*=0.032). |
| Farren *et al*. (2017)[25] | No difference in GDM. | NA. |
| Ferrara *et al*. (2020)[26] | No difference in GDM. | Women in the intervention group had lower average weekly GWG (MD –0.07 kg, 95% CI -0.09 – -0.04, *p*<0.0001). |
| Gallagher *et al*. (2018)[27] | No difference in GDM. | Women in the intervention group had less GWG (7.89 ± 4.07 kg vs. 9.67 ± 4.17 kg, *p*=0.003).  Infants in the intervention group had greater fat-free mass (*p*=0.03) and lean mass (*p*=0.006). |
| Guelfi *et al*. (2016)[28] | No difference in GDM. | Women in the intervention group have improved maternal fitness (*p*<0.01) and psychological distress (*p*=0.02). |
| Garnæs *et al*. (2016)[29] | GDM was reduced by 39% (n=2, 6.1% vs. n=9, 27.3%; OR 0.1, 95% CI 0.02–0.95, *p*=0.04) (**WHO, 2009**). | Systolic blood pressure was lower in the exercise group (MD−7.73 mm Hg, 95% CI −13.23-−2.22; *p=*0.006). |
| Gonzalez-Plaza *et al*. (2022)[30] | No difference in GDM. | The median GWG was lower in the intervention group (7.0 kg, IQR 4 −11 vs. 9.3 kg, IQR 5.9 − 13.3, *p*=0.04).  Women in the intervention group increased their physical activity (*p*=0.01). |
| Halkjær *et al*. (2020)[31] | No difference in GDM. | There was an increase in fecal microbiota α-diversity over time in the probiotic group (*p=*0.016). |
| Harrison *et al*. (2013)[32] | No difference in GDM. | GWG was lower in the intervention group compared to the controls (6.9 ± 3.3 kg vs. 6.0 ± 2.8 kg, *p*<0.05).  Overweight participants in the control group gained more weight in comparison to overweight participants in the intervention group (7.8 ± 3.4 kg vs. 6.0 ± 2.2 kg, *p*<0.05).  The intervention group had a 20% higher step count compared to controls (*p*<0.05). |
| Herring *et al*. (2015)[33] | No difference in GDM. | GWG was lower in the intervention group compared to the controls (8.7 vs. 12.3 kg, MD− 23.1 kg, 95% CI 26.2–20.1). |
| Jamal, Milani and Al-Yasin. (2012)[34] | No difference in GDM. | NA. |
| Kennelly *et al*. (2018)[35] | No difference in GDM. | The intervention group had lower dietary glycaemic index and glycaemic load and increased exercise levels (all *p*=0.02). |
| Koivusalo *et al.* (2016)[36] | GDM was reduced by 39% (n=20, 13.9% vs. n=27, 21.6%; RR 0.64, 95% CI 0.40–0.98, *p*=0.044) (**ADA, 2008**). | GWG from baseline to the 2^nd^ trimester was lower in the intervention group compared to the control group (MD−2.5 kg, 95% CI 2.1–3.0 vs. 3.1 kg, 95% CI 2.7–3.5, *p*=0.037).  Women in the intervention group improved their diet quality (*p*=0.037) and increased their leisure-time physical activity (*p*=0.029). |
| Korpi-Hyovalti *et al.* (2011)[37] | No difference in GDM. | Mean birthweight was higher in the lifestyle intervention arm compared with the close follow-up group (3871 ± 567g vs. 3491 ± 573g, *p*=0.047).  There was an increase in polyunsaturated fatty acid intake in the intervention group (*p*=0.008), while the intake of saturated fat exceeded the recommendations in both groups. |
| Kong *et al.* (2014)[38] | No difference in GDM. | Women in the intervention group increased their moderate-intensity physical activity and meaningful walks (*p*<0.05). |
| LeBlanc *et al*. (2022)[39] | No difference in GDM. | Women in the intervention group had more GWG compared to controls (13.2 ± 8.20 kg vs. 10.3 ± 7.41 kg, *p*=0.03). |
| Lin *et al.* (2020)[40] | The intervention group showed a reduction in GDM (n=20, 14.4% vs. n=35, 24.6%; aOR 0.45, 95% CI 0.22–0.86, *p*<0.01) (**ADA, 2017**). | Women in the intervention group had less GWG compared to controls (OR.0.42, 95% CI: 0.28–0.69, *p*<0.01).  The incidences of adverse maternal outcomes, including caesarean delivery (35.3% vs. 47.9%), PIH, PROM, antepartum haemorrhage and postpartum haemorrhage were lower in the intervention group compared to the control group (all *p*<0.05). |
| Liu *et al*. (2021)[41] | No difference in GDM. | Women in the intervention group had fewer low birthweight infants and lower PIH rates. |
| Lovvik *et al.* (2019)[42] | No difference in GDM. | NA. |
| Luoto *et al.* (2011)[43] | No difference in GDM. | The proportion of LGA infants was lower in the intervention group compared with the control group (n= 26, 12.1% vs. n= 34, 19.7%, *p*=0.042).  Birthweight was lower in the intervention group compared with the control group (absolute effect size -133g, 95% CI -231 –-35, *p*=0.008).  There was a reduction in saturated fat and saccharose consumption and an increase in polyunsaturated fatty acid and dietary fibre intake in the intervention group (all *p*<0.05). |
| Matarrelli *et al*. (2013)[44] | The MI group showed a reduction in GDM (n= 2, 6% vs. n= 27, 71%; RR 0.127, 95% CI 0.032–0.502, *p*=0.001) (**IADPSG**). | Infants’ birthweight was lower in the MI group when expressed as percentiles (42.8 ± 20.4 vs. 56.6 ± 25.9, 95% CI 10.807–30.116, *p*=0.001).  The MI group had fewer events of neonatal hypoglycaemia (n=0, 0% vs. n=10, 26%; RR 0.052, 95% CI 0.003–0.849, *p*=0.038). |
| McCarthy *et al.* (2016)[45] | No difference in GDM. | NA. |
| Mohsenzadeh-Ledari *et al*. (2020)[46] | The intervention group showed a reduction in GDM (n= 7, 13% vs. n=17. 34.5%; aOR 3.59, 95% CI 1.23–10.45, *p*=0.01) (**criteria not stated**). | Women in the intervention group had less GWG compared to the controls (9.07 ± 4.85 kg vs. 11.83 ± 5.50 kg, *p*=0.007).  The 2-hr glucose values in the 7th month of pregnancy were lower in the intervention group (107.88 ± 27.48 mg/dL vs. 126.14 ± 30.87 mg/dL, *p*=0.007).  Less women in the intervention group required hospitalisation (aOR3.31, 95% CI 0.89–12.27, *p*=0.07) and diet (aOR 3.59, 95% CI 1.23–10. 45, *p*=0.01) due to GDM. |
| Motahari-Tabari *et al.* (2021)[47] | The prevalence of GDM was reduced in the intervention group (n= 7, 10% vs. n=20. 29%; RR 0.33, 95% CI 0.15–0.74, *p*=0.004) (**WHO, 2013**). | 1-hr and 2-hr glucose values were lower in the intervention group (*p*<0.01). |
| Okesene-Gafa *et al*. (2019)[48] | No difference in GDM. | Women who received the dietary intervention group had less GWG compared with controls (9.7 ± 6.6 kg vs. 11.4 ± 6.3 kg, *p*=0.05). |
| Oostdam *et al*. (2012)[49] | No difference in GDM. | NA. |
| Parat *et al.* (2019)[50] | No difference in GDM. | NA. |
| Peccei *et al.* (2017)[51] | No difference in GDM. | Among women with obesity, the intervention was associated with fewer LGA infants (7% vs.17%; OR 0.3, 95% CI 0.1–0.99). |
| Pellonperä *et al.* (2019)[52] | No difference in GDM. | NA. |
| Petrella *et al. (*2013)[53] | The intervention group showed a reduction in GDM (n= 7, 23.3% vs. n=16. 57.1%, *p*=0.009) **(ADA, 2011)**. | GWG was lower in women with obesity in the intervention group (6.7 ± 4.3 vs. 10.1 ± 5.6 kg; *p=*0.047).  The rate of PIH (n= 1, 3% vs. n=7. 25%, *p*=0.011) and preterm delivery (n= 0, 0% vs. n=10. 35.7%, *p*=0.0009) were lower in the intervention group.  The intervention group showed positive changes in eating habits; higher intake of snacks, fruits and vegetables while decreased intake of sugar. |
| Phelan *et al.* (2018)[54] | No difference in GDM. | The intervention resulted in less GWG (9.4 ± 6.9 vs. 11.2 ± 7.0 kg; *p=*0.03).  The intervention improved weight-control strategies including diet and physical activity (*p*<0.0001). |
| Phelan *et al.* (2023)[55] | No difference in GDM. | Independent of group allocation, a 5% weight loss before conception reduced the odds of GDM recurrence by 82% (OR 0.03, 95%, CI 0.04–0.88, *p*=0.04). |
| Phillips *et al.* (2019)[56] | No difference in GDM. | NA. |
| Poston *et al.* (2015)[57] | No difference in GDM. | Women in the intervention group had less GWG (MD−0.55 kg, 95% CI −1.08 –−0.02; *p*=0.041).  The intervention group showed improvement in dietary glycaemic load, physical activity and maternal sum-of-skinfold thicknesses (all *p*<0.05). |
| Quinlivan, Lam and Fisher. (2011)[58] | There was a reduction in the incidence of GDM in the intervention group (n= 4, 6% vs. n=17, 29%; OR 0.17, 95%, CI 0.03–0.95, *p=*0.04) **(WHO, 2009)**. | The intervention group gained less GWG (7.0 ± 0.65 kg vs. 13.8 ± 0.67 kg, *p*<0.0001). |
| Rakhshani *et al*. (2012)[59] | The intervention group showed a reduction in GDM (n= 3, 10.3% vs. n=11, 36.7%, *p=*0.049) **(ADA, 2004)**. | The intervention group had fewer cases of PIH, pre-eclampsia, SGA and IUGR (*p*=0.018, 0.042, 0.033, 0.05 respectively). |
| Renault *et al*. (2014)[60] | No difference in GDM. | Women in the diet + physical activity group had lower rate of c-section compared to the control group (n= 32, 25% vs. n=50, 37%, *p=*0.016). |
| Roeder *et al.* (2019)[61] | No difference in GDM. | NA. |
| Sadiya *et al*. (2022)[62] | The incidence of GDM in the intervention group was lower than in the control group (n=10, 33.3% vs. n=19, 57.5%; aOR 0.26, 95% CI 0.07–0.92, *p=*0.037) (**IADPSG**). | Women in the intervention group reduced their daily energy, carbohydrates and fat consumption and increased the physical activity time (all *p*≤0.05). |
| Sales *et al*. (2018)[63] | No difference in GDM. | NA. |
| Santamaria *et al*. (2016)[64] | The incidence of GDM was reduced by 67% in the intervention group (n= 11, 11.6% vs. n=28, 27.4%, OR 0.33, 95%, CI 0.15–0.70, *p=*0.004) **(IADPSG)**. | NA. |
| Sartorelli *et al*. (2022)[65] | No difference in GDM. | Women in the intervention group had a lower chance of having excessive GWG (OR 0.56, 95% CI 0.32 –0.98, *p* = 0.04). |
| Seneviratne *et al.* (2016)[66] | No difference in GDM. | Women in the intervention group had improved their aerobic fitness compared with controls (48.0-second improvement in test time to target heart rate; *p=*0.019). |
| Shahgheibi, Farhadifar and Pouya. (2016)[67] | The intervention group showed a reduction in GDM incidence (n=5, 11.4% vs. n=15, 34.8%; *p*<0.01) **(ADA classification of DM)**. | NA. |
| Shahriari *et al*. (2021)[68] | No difference in GDM. | NA. |
| Simmons *et al*. (2017)[69] | No difference in GDM. | The combined diet + physical activity intervention was associated with lower GWG compared to usual care.  Both diet only and diet + physical activity interventions were associated with improvements in healthy eating while the physical activity intervention was associated with greater moderate-to-vigorous exercise. |
| Syngelaki *et al.* (2016) [70] | No difference in GDM. | The median GWG was lower in the intervention group (4.6 kg, IQR 1.3 −7.2 vs. 6.3 kg, IQR 2.9 − 9.2, *p*<0.001) as was the incidence of pre-eclampsia (3.0% vs. 11.3%; OR 0.24, 95% CI 0.10 −0.61; *p*=0.001). |
| Thornton *et al*. (2009)[71] | No difference in GDM. | Women in the intervention group were less likely to have PIH compared to controls (n=3, 3% vs. n=10, 9%; *p=*0.046). |
| Valdés et al. (2018)[72] | No difference in GDM. | NA. |
| Van Horn *et al.* (2018)[73] | No difference in GDM. | Women who received the intervention had less GWG compared with controls (10 ± 6 kg vs. 12 ± 6 kg, *p*=0.02).  The rate of c-sections was higher in the intervention group than in the control group (55, 40% vs. 37,27%, *p*=0.03).  The intervention group showed adherence to the DASH diet guidelines and a higher Healthy Eating Index scores with an increase in the consumption of whole grains and dairy products (all *p*<0.05). |
| Vanky *et al*. (2004)[74] | No difference in GDM. | NA. |
| Vanky *et al*. (2010)[75] | No difference in GDM. | Women in the intervention group had less GWG compared with controls (MD−2.2 kg, *p*=0.001). |
| Vesco *et al*. (2014)[76] | No difference in GDM. | Women in the intervention group had less GWG compared with controls (MD−3.4 kg, 95% CI −5.1−1.8; *p*<0.001).  The intervention led to a lower prevalence of LGA infants (n=5, 9% vs. n=15, 26%; *p*=0.02). |
| Vinter *et al*. (2011)[77] | No difference in GDM. | The median GWG was lower in the intervention group (7.0 kg, IQR 4.7 −10.6 vs. 8.6 kg, IQR 5.7 − 11.5, *p*<0.01). |
| Vitale *et al*. (2021)[78] | The incidence of GDM was reduced in the intervention group (n= 9, 8.2% vs. n=24, 21.2%; OR 3.74, 95%, CI 1.67–8.39, *p*=0.0014) **(IADPSG).** | Women in the intervention group were less likely to have PIH compared to controls (n=8, 7.3% vs. n=21.2, 9%; *p*=0.0434).  There was an increase in total body water values in the intervention group (*p*<0.0001). |
| Walsh *et al*. (2012)[79] | No difference in GDM. | Women in the intervention group had less GWG compared to the control group (MD−1.3 kg, 95% CI −2.4 −−0.2; *p*=0.01).  The proportion of glucose intolerance was higher in the control group (28% vs. 21%, *p*=0.02).  The intervention group had a lower glycaemic index/glycaemic load and energy intake in the 2^nd^ and 3^rd^ trimesters and higher fibre intake in the 3^rd^ trimester (all *p*<0.01). |
| Wang *et al*. (2017)[80] | Women in the intervention group had a 45.8% lower incidence of GDM (n= 29, 22% vs. n=54, 40.6%; OR 0.412, 95%, CI 0.240–0.705, *p=*0.001) **(WHO,2013).** | Women allocated to the intervention group had less GWG (8.38 ± 3.65 kg vs. 10.47 ± 3.33 kg; *p*<0.001) and a lower birthweight compared to the control group (3345.27 ± 397.07 vs. 3457.46 ± 446.00 g, *p*=0.049). |
| Wang *et al*. (2015)[81] | No difference in GDM. | NA. |
| Wolff *et al*. (2008)[82] | No difference in GDM. | Women in the intervention group limited their energy intake (*p*=0.001) and had less GWG compared to the control group (MD−6.7 kg, 95% CI −2.6 −10.8; *p*=0.002).  Both serum insulin and leptin were reduced in the intervention group (*p*<0.05). |
| Zhang *et al*. (2019)[83] | No difference in GDM. | There was an increase in dietary fibre intake in the intervention group (*p*=0.006). |
| Zhang *et al*. (2022)[84] | The incidence of GDM was reduced in the intervention group (n= 4, 8.3% vs. n=12, 24.0%; χ2 = 4.40, *p=*0.036) **(IADPSG).** | The intervention group had lower GWG (1.99 ± 1.09 kg vs. 2.53 ± 1.20 kg, *p=*0.022).  The intervention group had a lower FBG values at the OGTT compared with the control group (*p*< 0.01). |

ADA= American Diabetes Association; aOR= adjusted odd ratio; CI= confidence interval; C= control; DCI= D-chiro-inositol; DM= diabetes mellitus; FBG= fasting blood glucose; GCT= glucose challenge test; GDM= gestational diabetes mellitus; GWG= gestational weight gain; hr= hour; IADPSG= The International Association of Diabetes and Pregnancy Study Group; IUGR= intrauterine growth restriction; LGA= large-for-gestational-age; MD= mean difference; MI= myo-inositol; MI/DCI= myo-inositol and D-chiro-inositol; NA= not applicable; n= number; OGTT= oral glucose tolerance test; OR= odd ratio; PIH= pregnancy induced hypertension; RR= relative risk; SGA= small-for-gestational age; vs.= versus

#

# **Table A7.** Eggers test of publication bias for antenatal interventions

| Intervention | **Parameter** | **Coefficient** | **Standard error** | **t value** | ***p* value** | **95% Confidence interval** | |
| --- | --- | --- | --- | --- | --- | --- | --- |
| Diet and physical activity | slope | 0.0608737 | 0.0161125 | 3.78 | 0.001 | 0.0280536 | 0.0936937 |
|  | bias | -2.099599 | 0.4082775 | -5.14 | <0.05 | -2.931233 | -1.267965 |
|  | Test of H0: no small-study effects *p*<0.05, Number of studies = 34, Root MSE = 1.149 | | | | | | |
| Physical activity only | slope | -0.0445892 | 0.039539 | 1.13 | 0.286 | -0.1326876 | 0.0435091 |
|  | bias | 0.1145722 | 0.7178684 | 0.16 | 0.876 | -1.484938 | 1.714083 |
|  | Test of H0: no small-study effects *p*= 0.876, Number of studies = 12, Root MSE = 1.28 | | | | | | |
| Metformin | slope | 0.0472607 | 0.0509467 | 0.93 | 0.378 | 0.0679888 | 0.1625102 |
|  | bias | -1.060098 | 0.9974916 | -1.06 | 0.316 | -3.316581 | 1.196385 |
|  | Test of H0: no small-study effects *p*= 0.316, Number of studies = 11, Root MSE = 1.39 | | | | | | |

Egger's test for small-study effects: Regression of the standard normal deviate of intervention effect estimate against its standard error. Interventions with significant evidence of publication bias are highlighted in red.

**Figure A1.** Funnel plots of publication bias for antenatal interventions

| 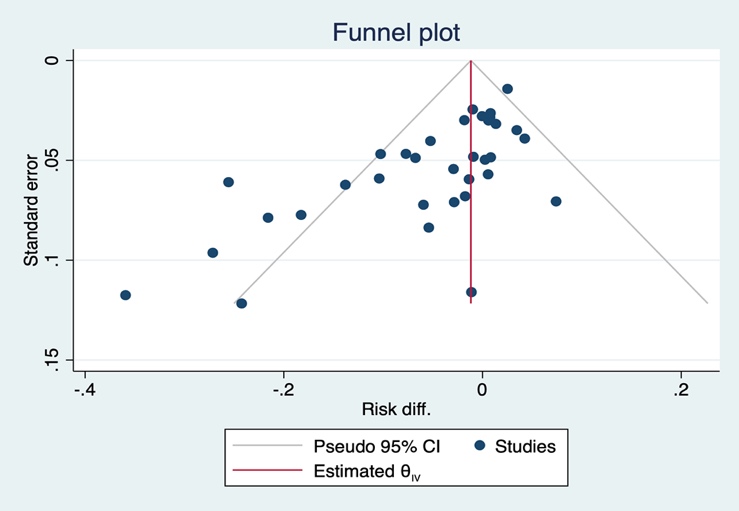A)  **Diet and physical activity** | 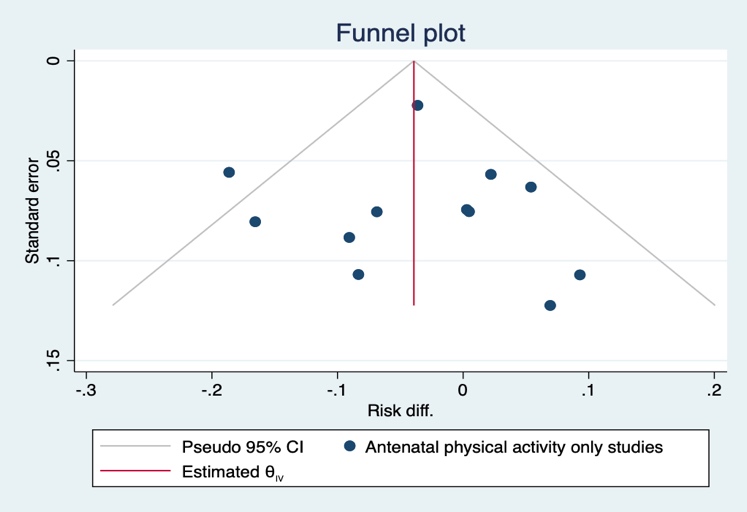B) P**hysical activity only** | C) **Metformin**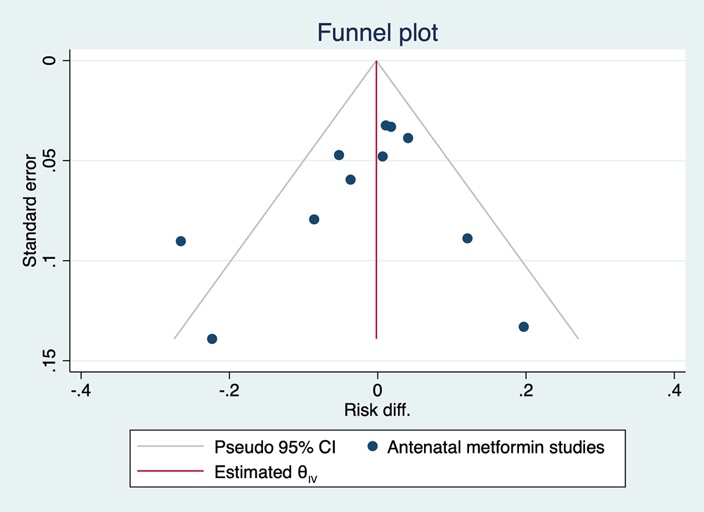 |
| --- | --- | --- |

**References**

1. Ajmani SN, Simantini S. Role of vitamin D supplementation in preventing development of gestational diabetes mellitus . An Int J Obstet Gynaecol [Internet]. 2020 [cited 2022 Jun 6];10. Available from: https://www.iog.org.in/index.php/iog/article/view/610

2. Amaefule CE, Drymoussi Z, Gonzalez Carreras FJ, Pardo Llorente MDC, Lanz D, Dodds J, et al. Myo-inositol nutritional supplement for prevention of gestational diabetes (EMmY): a randomised, placebo-controlled, double-blind pilot trial with nested qualitative study. BMJ Open [Internet]. British Medical Journal Publishing Group; 2022 [cited 2022 Apr 15];12:e050110. Available from: https://bmjopen.bmj.com/content/12/3/e050110

3. H. Al Wattar B, Dodds J, Placzek A, Beresford L, Spyreli E, Moore A, et al. Mediterranean-style diet in pregnant women with metabolic risk factors (ESTEEM): A pragmatic multicentre randomised trial. Persson LÅ, editor. PLOS Med. Public Library of Science; 2019;16:e1002857.

4. Basu A, Feng D, Planinic P, Ebersole JL, Lyons TJ, Alexander JM. Dietary Blueberry and Soluble Fiber Supplementation Reduces Risk of Gestational Diabetes in Women with Obesity in a Randomized Controlled Trial. J Nutr [Internet]. Oxford University Press (OUP); 2021 [cited 2021 May 7]; Available from: https://pubmed.ncbi.nlm.nih.gov/33693835/

5. Begum MR, Khanam NN, Quadir E, Ferdous J, Begum MS, Khan F, et al. Prevention of gestational diabetes mellitus by continuing metformin therapy throughout pregnancy in women with polycystic ovary syndrome. J Obstet Gynaecol Res [Internet]. J Obstet Gynaecol Res; 2009 [cited 2022 Oct 27];35:282–6. Available from: https://pubmed.ncbi.nlm.nih.gov/19708174/

6. Bisson M, Alméras N, Dufresne SS, Robitaille J, Rhéaume C, Bujold E, et al. A 12-Week Exercise Program for Pregnant Women with Obesity to Improve Physical Activity Levels: An Open Randomised Preliminary Study. PLoS One [Internet]. PLoS One; 2015 [cited 2022 Apr 18];10. Available from: https://pubmed.ncbi.nlm.nih.gov/26375471/

7. Brink HS, Alkemade M, van der Lely AJ, van der Linden J. Metformin in women at high risk of gestational diabetes mellitus. Diabetes Metab [Internet]. Diabetes Metab; 2018 [cited 2022 Jun 8];44:300–2. Available from: https://pubmed.ncbi.nlm.nih.gov/29422358/

8. Bogaerts AFL, Devlieger R, Nuyts E, Witters I, Gyselaers W, Van Den Bergh BRH. Effects of lifestyle intervention in obese pregnant women on gestational weight gain and mental health: a randomized controlled trial. Int J Obes (Lond) [Internet]. Int J Obes (Lond); 2013 [cited 2022 Apr 18];37:814–21. Available from: https://pubmed.ncbi.nlm.nih.gov/23032404/

9. Bruno R, Petrella E, Bertarini V, Pedrielli G, Neri I, Facchinetti F. Adherence to a lifestyle programme in overweight/obese pregnant women and effect on gestational diabetes mellitus: a randomized controlled trial. Matern Child Nutr [Internet]. John Wiley & Sons, Ltd; 2017 [cited 2022 Apr 19];13:e12333. Available from: https://onlinelibrary.wiley.com/doi/full/10.1111/mcn.12333

10. Callaway LK, Colditz PB, Byrne NM, Lingwood BE, Rowlands IJ, Foxcroft K, et al. Prevention of Gestational DiabetesFeasibility issues for an exercise intervention in obese pregnant women. Diabetes Care [Internet]. American Diabetes Association; 2010 [cited 2022 Apr 19];33:1457–9. Available from: https://diabetesjournals.org/care/article/33/7/1457/39270/Prevention-of-Gestational-DiabetesFeasibility

11. Callaway LK, McIntyre HD, Barrett HL, Foxcroft K, Tremellen A, Lingwood BE, et al. Probiotics for the Prevention of Gestational Diabetes Mellitus in Overweight and Obese Women: Findings From the SPRING Double-Blind Randomized Controlled Trial. Diabetes Care [Internet]. Diabetes Care; 2019 [cited 2022 Apr 20];42:364–71. Available from: https://pubmed.ncbi.nlm.nih.gov/30659070/

12. Celentano C, Matarrelli B, Pavone G, Vitacolonna E, Mattei PA, Berghella V, et al. The influence of different inositol stereoisomers supplementation in pregnancy on maternal gestational diabetes mellitus and fetal outcomes in high-risk patients: a randomized controlled trial. J Matern Neonatal Med. Taylor and Francis Ltd; 2018;33:743–51.

13. Chan RSM, Tam WH, Ho ICH, Kwan MWC, Li LS, Sea MMM, et al. Randomized trial examining effectiveness of lifestyle intervention in reducing gestational diabetes in high risk Chinese pregnant women in Hong Kong. Sci Rep. Nature Publishing Group; 2018;8.

14. Chiswick C, Reynolds RM, Denison F, Drake AJ, Forbes S, Newby DE, et al. Effect of metformin on maternal and fetal outcomes in obese pregnant women (EMPOWaR): A randomised, double-blind, placebo-controlled trial. Lancet Diabetes Endocrinol. Lancet Publishing Group; 2015;3:778–86.

15. Corcoy R, Mendoza LC, Simmons D, Desoye G, Adelantado JM, Chico A, et al. The DALI vitamin D randomized controlled trial for gestational diabetes mellitus prevention: No major benefit shown besides vitamin D sufficiency. Clin Nutr [Internet]. Churchill Livingstone; 2020 [cited 2022 Apr 20];39:976–84. Available from: https://research.vumc.nl/en/publications/the-dali-vitamin-d-randomized-controlled-trial-for-gestational-di

16. D’Anna R, Scilipoti A, Giordano D, Caruso C, Cannata ML, Interdonato ML, et al. Myo-inositol supplementation and onset of gestational diabetes mellitus in pregnant women with a family history of type 2 diabetes: A prospective, randomized, placebo-controlled study. Diabetes Care. Diabetes Care; 2013;36:854–7.

17. D’Anna R, Di Benedetto A, Scilipoti A, Santamaria A, Interdonato ML, Petrella E, et al. Myo-inositol Supplementation for Prevention of Gestational Diabetes in Obese Pregnant Women: A Randomized Controlled Trial. Obstet Gynecol [Internet]. Obstet Gynecol; 2015 [cited 2022 Aug 11];126:310–5. Available from: https://pubmed.ncbi.nlm.nih.gov/26241420/

18. Daly N, Farren M, McKeating A, O’Kelly R, Stapleton M, Turner MJ. A Medically Supervised Pregnancy Exercise Intervention in Obese Women: A Randomized Controlled Trial. Obstet Gynecol [Internet]. Obstet Gynecol; 2017 [cited 2022 Apr 20];130:1001–10. Available from: https://pubmed.ncbi.nlm.nih.gov/29016485/

19. Deng Y, Hou Y, Wu L, Liu Y, Ma L, Yao A. Effects of Diet and Exercise Interventions to Prevent Gestational Diabetes Mellitus in Pregnant Women With High-Risk Factors in China: A Randomized Controlled Study. Clin Nurs Res [Internet]. SAGE PublicationsSage CA: Los Angeles, CA; 2021 [cited 2022 Apr 21];10547738211055576. Available from: http://www.ncbi.nlm.nih.gov/pubmed/34775875

20. Ding B, Gou B, Guan H, Wang J, Bi Y, Hong Z. WeChat-assisted dietary and exercise intervention for prevention of gestational diabetes mellitus in overweight/obese pregnant women: a two-arm randomized clinical trial. Arch Gynecol Obstet [Internet]. Springer Science and Business Media Deutschland GmbH; 2021 [cited 2022 Apr 22];304:609–18. Available from: https://link.springer.com/article/10.1007/s00404-021-05984-1

21. Dodd JM, Louise J, Deussen AR, Grivell RM, Dekker G, McPhee AJ, et al. Effect of metformin in addition to dietary and lifestyle advice for pregnant women who are overweight or obese: the GRoW randomised, double-blind, placebo-controlled trial. Lancet Diabetes Endocrinol. Lancet Publishing Group; 2019;7:15–24.

22. Dodd JM, Cramp C, Sui Z, Yelland LN, Deussen AR, Grivell RM, et al. The effects of antenatal dietary and lifestyle advice for women who are overweight or obese on maternal diet and physical activity: The LIMIT randomised trial. BMC Med [Internet]. BioMed Central Ltd.; 2014 [cited 2021 Nov 14];12:1–19. Available from: https://link.springer.com/articles/10.1186/s12916-014-0161-y

23. Eslami E, Mohammad Alizadeh Charandabi S, Khalili AF, Jafarabadi MA, Mirghafourvand M. The effect of a lifestyle-based training package on weight gain and frequency of gestational diabetes in obese and overweight pregnant females. Iran Red Crescent Med J. Kowsar Medical Publishing Company; 2018;20.

24. Esmaeilzadeh S, Ghadimi R, Mashayekh-Amiri S, Delavar MA, Basirat Z. The effect of myo-inositol supplementation on the prevention of gestational diabetes in overweight pregnant women: a randomized, double-blind, controlled trial. Minerva Obstet Gynecol [Internet]. Minerva Obstet Gynecol; 2022 [cited 2023 Feb 14]; Available from: https://pubmed.ncbi.nlm.nih.gov/35686634/

25. Farren M, Daly N, McKeating A, Kinsley B, Turner MJ, Daly S. The prevention of gestational diabetes mellitus with antenatal oral inositol supplementation: A randomized controlled trial. Diabetes Care. American Diabetes Association Inc.; 2017;40:759–63.

26. Ferrara A, Hedderson MM, Brown SD, Ehrlich SF, Tsai AL, Feng J, et al. A telehealth lifestyle intervention to reduce excess gestational weight gain in pregnant women with overweight or obesity (GLOW): a randomised, parallel-group, controlled trial. Lancet Diabetes Endocrinol [Internet]. Elsevier; 2020 [cited 2022 May 12];8:490–500. Available from: http://www.thelancet.com/article/S2213858720301078/fulltext

27. Gallagher D, Rosenn B, Toro-Ramos T, Paley C, Gidwani S, Horowitz M, et al. Greater neonatal fat-free mass and similar fat mass following a randomized trial to control excess gestational weight gain. Obesity (Silver Spring) [Internet]. NIH Public Access; 2018 [cited 2022 Jun 8];26:578. Available from: /pmc/articles/PMC5824435/

28. Guelfi KJ, Ong MJ, Crisp NA, Fournier PA, Wallman KE, Grove JR, et al. Regular Exercise to Prevent the Recurrence of Gestational Diabetes Mellitus: A Randomized Controlled Trial. Obstet Gynecol [Internet]. Lippincott Williams and Wilkins; 2016 [cited 2022 Jun 8];128:819–27. Available from: https://europepmc.org/article/med/27607876

29. Garnæs KK, Mørkved S, Salvesen Ø, Moholdt T. Exercise Training and Weight Gain in Obese Pregnant Women: A Randomized Controlled Trial (ETIP Trial). PLOS Med [Internet]. Public Library of Science; 2016 [cited 2022 May 12];13:e1002079. Available from: https://journals.plos.org/plosmedicine/article?id=10.1371/journal.pmed.1002079

30. Gonzalez-Plaza E, Bellart J, Arranz Á, Luján-Barroso L, Mirasol EC, Seguranyes G. Effectiveness of a Step Counter Smartband and Midwife Counseling Intervention on Gestational Weight Gain and Physical Activity in Pregnant Women With Obesity (Pas and Pes Study): Randomized Controlled Trial. JMIR mHealth uHealth [Internet]. JMIR Mhealth Uhealth; 2022 [cited 2023 Feb 19];10. Available from: https://pubmed.ncbi.nlm.nih.gov/35166684/

31. Halkjær SI, De Knegt VE, Lo B, Nilas L, Cortes D, Pedersen AE, et al. Multistrain Probiotic Increases the Gut Microbiota Diversity in Obese Pregnant Women: Results from a Randomized, Double-Blind Placebo-Controlled Study. Curr Dev Nutr [Internet]. American Society for Nutrition; 2020 [cited 2022 May 16];4. Available from: /pmc/articles/PMC7319727/

32. Harrison CL, Lombard CB, Strauss BJ, Teede HJ. Optimizing healthy gestational weight gain in women at high risk of gestational diabetes: A randomized controlled trial. Obesity. Blackwell Publishing Inc.; 2013;21:904–9.

33. Herring SJ, Cruice JF, Bennett GG, Rose MZ, Davey A, Foster GD. Preventing excessive gestational weight gain among African American women: A randomized clinical trial. Obesity (Silver Spring) [Internet]. Obesity (Silver Spring); 2015 [cited 2022 Oct 31];24:30–6. Available from: https://pubmed.ncbi.nlm.nih.gov/26592857/

34. Jamal A, Milani F, Al-Yasin A. Evaluation of the effect of metformin and aspirin on utero placental circulation of pregnant women with PCOS. Iran J Reprod Med [Internet]. Shahid Sadoughi University of Medical Sciences and Health Services; 2012 [cited 2022 Oct 27];10:265. Available from: /pmc/articles/PMC4165971/

35. Kennelly MAA, Kate, Lindsay KL, O’Sullivan E, Gibney ER, McCarthy M, et al. Pregnancy Exercise and Nutrition With Smartphone Application Support: A Randomized Controlled Trial. Obstet Gynecol [Internet]. Obstet Gynecol; 2018 [cited 2022 May 16];131:818–26. Available from: https://pubmed.ncbi.nlm.nih.gov/29630009/

36. Koivusalo SB, Rönö K, Klemetti MM, Roine RP, Lindström J, Erkkola M, et al. Gestational Diabetes Mellitus Can Be Prevented by Lifestyle Intervention: The Finnish Gestational Diabetes Prevention Study (RADIEL): A Randomized Controlled Trial. Diabetes Care. American Diabetes Association Inc.; 2016;39:24–30.

37. Korpi-Hyövälti EAL, Laaksonen DE, Schwab US, Vanhapiha TH, Vihla KR, Heinonen ST, et al. Feasibility of a lifestyle intervention in early pregnancy to prevent deterioration of glucose tolerance. BMC Public Health. BioMed Central; 2011;11:179.

38. Kong KL, Campbell CG, Foster RC, Peterson AD, Lanningham-Foster L. A pilot walking program promotes moderate-intensity physical activity during pregnancy. Med Sci Sports Exerc [Internet]. Med Sci Sports Exerc; 2014 [cited 2022 May 16];46:462–71. Available from: https://pubmed.ncbi.nlm.nih.gov/24002348/

39. LeBlanc ES, Smith NX, Vesco KK, Paul IM, Stevens VJ. Weight loss prior to pregnancy and subsequent gestational weight gain: Prepare, a randomized clinical trial. Am J Obstet Gynecol [Internet]. Am J Obstet Gynecol; 2021 [cited 2023 Feb 11];224:99.e1-99.e14. Available from: https://pubmed.ncbi.nlm.nih.gov/32687819/

40. Lin X, Yang T, Zhang X, Wei W. Lifestyle intervention to prevent gestational diabetes mellitus and adverse maternal outcomes among pregnant women at high risk for gestational diabetes mellitus. J Int Med Res [Internet]. SAGE Publications Ltd; 2020 [cited 2021 May 7];48:1–10. Available from: https://us.sagepub.com/en-us/nam/open-access-at-sage

41. Liu J, Wilcox S, Wingard E, Turner-McGrievy G, Hutto B, Burgis J. A Behavioral Lifestyle Intervention to Limit Gestational Weight Gain in Pregnant Women with Overweight and Obesity. Obesity (Silver Spring) [Internet]. Obesity (Silver Spring); 2021 [cited 2022 May 17];29:672–80. Available from: https://pubmed.ncbi.nlm.nih.gov/33619910/

42. Løvvik TS, Carlsen SM, Salvesen Ø, Steffensen B, Bixo M, Gómez-Real F, et al. Use of metformin to treat pregnant women with polycystic ovary syndrome (PregMet2): a randomised, double-blind, placebo-controlled trial. lancet Diabetes Endocrinol [Internet]. Lancet Diabetes Endocrinol; 2019 [cited 2022 Oct 24];7:256–66. Available from: https://pubmed.ncbi.nlm.nih.gov/30792154/

43. Luoto R, Kinnunen TI, Aittasalo M, Kolu P, Raitanen J, Ojala K, et al. Primary Prevention of Gestational Diabetes Mellitus and Large-for-Gestational-Age Newborns by Lifestyle Counseling: A Cluster-Randomized Controlled Trial. PLoS Med. PLoS Med; 2011;8.

44. Matarrelli B, Vitacolonna E, D’Angelo M, Pavone G, Mattei PA, Liberati M, et al. Effect of dietary myo-inositol supplementation in pregnancy on the incidence of maternal gestational diabetes mellitus and fetal outcomes: A randomized controlled trial. J Matern Neonatal Med. J Matern Fetal Neonatal Med; 2013;26:967–72.

45. McCarthy EA, Walker SP, Ugoni A, Lappas M, Leong O, Shub A. Self-weighing and simple dietary advice for overweight and obese pregnant women to reduce obstetric complications without impact on quality of life: a randomised controlled trial. BJOG [Internet]. BJOG; 2016 [cited 2022 May 17];123:965–73. Available from: https://pubmed.ncbi.nlm.nih.gov/26875586/

46. Mohsenzadeh-ledari F, Taghizadeh Z, Keramat A, Moosazadeh M, Yazdani S, Najafi A, et al. The effect of caring intervention (physical activity, diet and counseling) on gestational diabetes for pregnant women with metabolic syndrome. J Matern Neonatal Med [Internet]. Taylor and Francis Ltd.; 2020 [cited 2021 May 7]; Available from: https://pubmed.ncbi.nlm.nih.gov/33243041/

47. Motahari-Tabari NS, Nasiri-Amiri F, Faramarzi M, Shirvani MA, Bakhtiari A, Omidvar S. The Effectiveness of Information-Motivation-Behavioral Skills Model on Self-Care Practices in Early Pregnancy to Prevent Gestational Diabetes Mellitus in Iranian Overweight and Obese Women: A Randomized Controlled Trial. Int Q Community Health Educ [Internet]. Int Q Community Health Educ; 2021 [cited 2022 May 18]; Available from: https://pubmed.ncbi.nlm.nih.gov/34056987/

48. Okesene-Gafa KAM, Li M, McKinlay CJD, Taylor RS, Rush EC, Wall CR, et al. Effect of antenatal dietary interventions in maternal obesity on pregnancy weight-gain and birthweight: Healthy Mums and Babies (HUMBA) randomized trial. Am J Obstet Gynecol [Internet]. Am J Obstet Gynecol; 2019 [cited 2022 May 18];221:152.e1-152.e13. Available from: https://pubmed.ncbi.nlm.nih.gov/30878323/

49. Oostdam N, Van Poppel MNM, Wouters MGAJ, Eekhoff EMW, Bekedam DJ, Kuchenbecker WKH, et al. No effect of the FitFor2 exercise programme on blood glucose, insulin sensitivity, and birthweight in pregnant women who were overweight and at risk for gestational diabetes: results of a randomised controlled trial. BJOG [Internet]. BJOG; 2012 [cited 2022 May 18];119:1098–107. Available from: https://pubmed.ncbi.nlm.nih.gov/22616913/

50. Parat S, Nègre V, Baptiste A, Valensi P, Bertrand AM, Chollet C, et al. Prenatal education of overweight or obese pregnant women to prevent childhood overweight (the ETOIG study): an open-label, randomized controlled trial. Int J Obes (Lond) [Internet]. Int J Obes (Lond); 2019 [cited 2022 May 18];43:362–73. Available from: https://pubmed.ncbi.nlm.nih.gov/30242235/

51. Peccei A, Blake-Lamb T, Rahilly D, Hatoum I, Bryant A. Intensive Prenatal Nutrition Counseling in a Community Health Setting: A Randomized Controlled Trial. Obstet Gynecol [Internet]. Obstet Gynecol; 2017 [cited 2022 May 22];130:423–32. Available from: https://pubmed.ncbi.nlm.nih.gov/28697099/

52. Pellonperä O, Mokkala K, Houttu N, Vahlberg T, Koivuniemi E, Tertti K, et al. Efficacy of Fish Oil and/or Probiotic Intervention on the Incidence of Gestational Diabetes Mellitus in an At-Risk Group of Overweight and Obese Women: A Randomized, Placebo-Controlled, Double-Blind Clinical Trial. Diabetes Care [Internet]. American Diabetes Association; 2019 [cited 2022 May 24];42:1009–17. Available from: https://diabetesjournals.org/care/article/42/6/1009/36025/Efficacy-of-Fish-Oil-and-or-Probiotic-Intervention

53. Petrella E, Malavolti M, Bertarini V, Pignatti L, Neri I, Battistini NC, et al. Gestational weight gain in overweight and obese women enrolled in a healthy lifestyle and eating habits program. J Matern Fetal Neonatal Med [Internet]. J Matern Fetal Neonatal Med; 2013 [cited 2022 May 24];27:1348–52. Available from: https://pubmed.ncbi.nlm.nih.gov/24175912/

54. Phelan S, Wing RR, Brannen A, McHugh A, Hagobian TA, Schaffner A, et al. Randomized controlled clinical trial of behavioral lifestyle intervention with partial meal replacement to reduce excessive gestational weight gain. Am J Clin Nutr [Internet]. American Society for Nutrition; 2018 [cited 2022 May 24];107:183. Available from: /pmc/articles/PMC6455030/

55. Phelan S, Jelalian E, Coustan D, Caughey AB, Castorino K, Hagobian T, et al. Randomized controlled trial of prepregnancy lifestyle intervention to reduce recurrence of gestational diabetes mellitus. Am J Obstet Gynecol [Internet]. Am J Obstet Gynecol; 2023 [cited 2023 Mar 14]; Available from: https://pubmed.ncbi.nlm.nih.gov/36758710/

56. Phillips JK, Skelly JM, Roberts LM, Bernstein IM, Higgins ST. Combined financial incentives and behavioral weight management to enhance adherence with gestational weight gain guidelines: a randomized controlled trial. Am J Obstet Gynecol MFM [Internet]. Am J Obstet Gynecol MFM; 2019 [cited 2022 May 24];1:42–9. Available from: https://pubmed.ncbi.nlm.nih.gov/33319756/

57. Poston L, Bell R, Croker H, Flynn AC, Godfrey KM, Goff L, et al. Effect of a behavioural intervention in obese pregnant women (the UPBEAT study): A multicentre, randomised controlled trial. Lancet Diabetes Endocrinol. Lancet Publishing Group; 2015;3:767–77.

58. Quinlivan JA, Lam LT, Fisher J. A randomised trial of a four-step multidisciplinary approach to the antenatal care of obese pregnant women. Aust N Z J Obstet Gynaecol [Internet]. Aust N Z J Obstet Gynaecol; 2011 [cited 2022 May 28];51:141–6. Available from: https://pubmed.ncbi.nlm.nih.gov/21466516/

59. Rakhshani A, Nagarathna R, Mhaskar R, Mhaskar A, Thomas A, Gunasheela S. The effects of yoga in prevention of pregnancy complications in high-risk pregnancies: a randomized controlled trial. Prev Med (Baltim) [Internet]. Prev Med; 2012 [cited 2022 May 28];55:333–40. Available from: https://pubmed.ncbi.nlm.nih.gov/22884667/

60. Renault KM, Nørgaard K, Nilas L, Carlsen EM, Cortes D, Pryds O, et al. The Treatment of Obese Pregnant Women (TOP) study: a randomized controlled trial of the effect of physical activity intervention assessed by pedometer with or without dietary intervention in obese pregnant women. Am J Obstet Gynecol [Internet]. Am J Obstet Gynecol; 2014 [cited 2022 May 30];210:134.e1-134.e9. Available from: https://pubmed.ncbi.nlm.nih.gov/24060449/

61. Roeder HA, Moore TR, Wolfson MT, Gamst AC, Ramos GA. Treating hyperglycemia in early pregnancy: a randomized controlled trial. Am J Obstet Gynecol MFM [Internet]. NLM (Medline); 2019 [cited 2021 May 6];1:33–41. Available from: https://pubmed.ncbi.nlm.nih.gov/33319755/

62. Sadiya A, Jakapure V, Shaar G, Adnan R, Tesfa Y. Lifestyle intervention in early pregnancy can prevent gestational diabetes in high-risk pregnant women in the UAE: a randomized controlled trial. BMC Pregnancy Childbirth [Internet]. BioMed Central Ltd; 2022 [cited 2023 Feb 10];22:1–8. Available from: https://bmcpregnancychildbirth.biomedcentral.com/articles/10.1186/s12884-022-04972-w

63. Sales WB, Do Nascimento IB, Dienstmann G, De Souza MLR, Da Silva GD, Silva JC. Effectiveness of Metformin in the Prevention of Gestational Diabetes Mellitus in Obese Pregnant Women. Rev Bras Ginecol Obstet [Internet]. Rev Bras Ginecol Obstet; 2018 [cited 2022 Jun 5];40:180–7. Available from: https://pubmed.ncbi.nlm.nih.gov/29702716/

64. Santamaria A, Di Benedetto A, Petrella E, Pintaudi B, Corrado F, D’Anna R, et al. Myo-inositol may prevent gestational diabetes onset in overweight women: a randomized, controlled trial. J Matern Fetal Neonatal Med [Internet]. J Matern Fetal Neonatal Med; 2016 [cited 2022 Jun 5];29:3234–7. Available from: https://pubmed.ncbi.nlm.nih.gov/26698911/

65. Sartorelli DS, Crivellenti LC, Baroni NF, de Andrade Miranda DEG, da Silva Santos I, Carvalho MR, et al. Effectiveness of a minimally processed food-based nutritional counselling intervention on weight gain in overweight pregnant women: a randomized controlled trial. Eur J Nutr [Internet]. Springer Science and Business Media Deutschland GmbH; 2022 [cited 2023 Feb 19];62:443–54. Available from: https://link.springer.com/article/10.1007/s00394-022-02995-9

66. Seneviratne SN, Jiang Y, Derraik JGB, McCowan LME, Parry GK, Biggs JB, et al. Effects of antenatal exercise in overweight and obese pregnant women on maternal and perinatal outcomes: a randomised controlled trial. BJOG [Internet]. BJOG; 2016 [cited 2022 Jun 5];123:588–97. Available from: https://pubmed.ncbi.nlm.nih.gov/26542419/

67. Shahgheibi S, Farhadifar F, Pouya B. The effect of vitamin D supplementation on gestational diabetes in high-risk women: Results from a randomized placebo-controlled trial. J Res Med Sci [Internet]. J Res Med Sci; 2016 [cited 2022 Jun 5];21. Available from: https://pubmed.ncbi.nlm.nih.gov/27904548/

68. Shahriari A, Karimi E, Shahriari M, Aslani N, khooshideh M, Arab A. The effect of probiotic supplementation on the risk of gestational diabetes mellitus among high-risk pregnant women: A parallel double-blind, randomized, placebo-controlled clinical trial. Biomed Pharmacother. Elsevier Masson; 2021;141:111915.

69. Simmons D, Devlieger R, Van Assche A, Jans G, Galjaard S, Corcoy R, et al. Effect of Physical Activity and/or Healthy Eating on GDM Risk: The DALI Lifestyle Study. J Clin Endocrinol Metab [Internet]. J Clin Endocrinol Metab; 2017 [cited 2022 Oct 28];102:903–13. Available from: https://pubmed.ncbi.nlm.nih.gov/27935767/

70. Syngelaki A, Nicolaides KH, Balani J, Hyer S, Akolekar R, Kotecha R, et al. Metformin versus placebo in obese pregnant women without diabetes mellitus. N Engl J Med. Massachussetts Medical Society; 2016;374:434–43.

71. Thornton YS, Smarkola C, Kopacz SM, Ishoof SB. Perinatal Outcomes in Nutritionally Monitored Obese Pregnant Women: A Randomized Clinical Trial. J Natl Med Assoc. Elsevier; 2009;101:569–77.

72. Valdés E, Sepúlveda-Martínez A, Candia P, Abusada N, Orellana R, Manukian B, et al. Metformin as a prophylactic treatment of gestational diabetes in pregnant patients with pregestational insulin resistance: A randomized study. J Obstet Gynaecol Res [Internet]. J Obstet Gynaecol Res; 2018 [cited 2022 Oct 24];44:81–6. Available from: https://pubmed.ncbi.nlm.nih.gov/29094444/

73. Van Horn L, Peaceman A, Kwasny M, Vincent E, Fought A, Josefson J, et al. Dietary Approaches to Stop Hypertension Diet and Activity to Limit Gestational Weight: Maternal Offspring Metabolics Family Intervention Trial, a Technology Enhanced Randomized Trial. Am J Prev Med [Internet]. Am J Prev Med; 2018 [cited 2022 Jun 7];55:603–14. Available from: https://pubmed.ncbi.nlm.nih.gov/30262148/

74. Vanky E, Salvesen KÅ, Heimstad R, Fougner KJ, Romundstad P, Carlsen SM. Metformin reduces pregnancy complications without affecting androgen levels in pregnant polycystic ovary syndrome women: results of a randomized study. Hum Reprod [Internet]. Hum Reprod; 2004 [cited 2022 Oct 23];19:1734–40. Available from: https://pubmed.ncbi.nlm.nih.gov/15178665/

75. Vanky E, Stridsklev S, Heimstad R, Romundstad P, Skogøy K, Kleggetveit O, et al. Metformin Versus Placebo from First Trimester to Delivery in Polycystic Ovary Syndrome: A Randomized, Controlled Multicenter Study. J Clin Endocrinol Metab [Internet]. Oxford Academic; 2010 [cited 2022 Oct 23];95:E448–55. Available from: https://academic.oup.com/jcem/article/95/12/E448/2835402

76. Vesco KK, Karanja N, King JC, Gillman MW, Leo MC, Perrin N, et al. Efficacy of a group-based dietary intervention for limiting gestational weight gain among obese women: a randomized trial. Obesity (Silver Spring) [Internet]. Obesity (Silver Spring); 2014 [cited 2022 Jun 7];22:1989–96. Available from: https://pubmed.ncbi.nlm.nih.gov/25164259/

77. Vinter CA, Jensen DM, Ovesen P, Beck-Nielsen H, Jørgensen JS. The LiP (Lifestyle in Pregnancy) study: a randomized controlled trial of lifestyle intervention in 360 obese pregnant women. Diabetes Care [Internet]. Diabetes Care; 2011 [cited 2022 Oct 23];34:2502–7. Available from: https://pubmed.ncbi.nlm.nih.gov/21972411/

78. Vitale SG, Corrado F, Caruso S, Di Benedetto A, Giunta L, Cianci A, et al. Myo-inositol supplementation to prevent gestational diabetes in overweight non-obese women: bioelectrical impedance analysis, metabolic aspects, obstetric and neonatal outcomes - a randomized and open-label, placebo-controlled clinical trial. Int J Food Sci Nutr [Internet]. Int J Food Sci Nutr; 2021 [cited 2022 Jun 7];72:670–9. Available from: https://pubmed.ncbi.nlm.nih.gov/33238798/

79. Walsh JM, McGowan CA, Mahony R, Foley ME, McAuliffe FM. Low glycaemic index diet in pregnancy to prevent macrosomia (ROLO study): Randomised control trial. BMJ. British Medical Journal Publishing Group; 2012;345.

80. Wang C, Wei Y, Zhang X, Zhang Y, Xu Q, Sun Y, et al. A randomized clinical trial of exercise during pregnancy to prevent gestational diabetes mellitus and improve pregnancy outcome in overweight and obese pregnant women. Am J Obstet Gynecol [Internet]. Elsevier; 2017 [cited 2022 Jun 8];216:340–51. Available from: http://www.ajog.org/article/S0002937817301722/fulltext

81. Wang S, Ma J-M, Yang H-X. Lifestyle intervention for gestational diabetes mellitus prevention: A cluster-randomized controlled study. Chronic Dis Transl Med. Elsevier BV; 2015;1:169–74.

82. Wolff S, Legarth J, Vangsgaard K, Toubro S, Astrup A. A randomized trial of the effects of dietary counseling on gestational weight gain and glucose metabolism in obese pregnant women. Int J Obes (Lond) [Internet]. Int J Obes (Lond); 2008 [cited 2022 Jun 8];32:495–501. Available from: https://pubmed.ncbi.nlm.nih.gov/18227847/

83. Zhang Y, Wang L, Yang W, Niu D, Li C, Wang L, et al. Effectiveness of Low Glycemic Index Diet Consultations Through a Diet Glycemic Assessment App Tool on Maternal and Neonatal Insulin Resistance: A Randomized Controlled Trial. JMIR mHealth uHealth [Internet]. JMIR Mhealth Uhealth; 2019 [cited 2022 Jun 8];7. Available from: https://pubmed.ncbi.nlm.nih.gov/30998227/

84. Zhang DY, Cheng DC, Cao YN, Su Y, Chen L, Liu WY, et al. The effect of dietary fiber supplement on prevention of gestational diabetes mellitus in women with pre-pregnancy overweight/obesity: A randomized controlled trial. Front Pharmacol. Frontiers Media S.A.; 2022;13:3409.
